# POMC AMPKα1 deletion drives male-specific BAT thermogenesis and obesity resistance

**DOI:** 10.1038/s44319-026-00915-0

**Published:** 2026-09-01

**Authors:** Óscar Freire-Agulleiro, Marcos Rios Garcia, Vitor Ferreira, Patricia Seoane-Collazo, Juan Roa, Ánxela Estévez-Salguero, Alicia Otero-Rey, Jordi Capellades, Francisco Ruiz-Pino, Pablo Garrido-Gil, Vincent Simon, Jean-Charles Nicolas, Juan A López, Cristina Contreras, Laura Liñares-Pose, Francisco Gonzalez, José L Labandeira-García, Jesús Vázquez, Carlos Diéguez, Rubén Nogueiras, Daniela Cota, Oscar Yanes, Guadalupe Sabio, Manuel Tena-Sempere, Carmelo Quarta, Ismael González-García, Miguel López

**Affiliations:** 1 Department of Physiology, CiMUS, University of Santiago de Compostela, Santiago de Compostela, 15782 Spain; 2 CIBER Fisiopatología de la Obesidad y Nutrición (CIBEROBN), Santiago de Compostela, 15706 Spain; 3 Department of Cell Biology, Physiology and Immunology, University of Córdoba; Instituto Maimónides de Investigación Biomédica (IMIBIC)/Hospital Universitario Reina Sofía, Córdoba, 14004 Spain; 4 CIBER of Diabetes and Associated Metabolic Diseases (CIBERDEM), Instituto de Salud Carlos III (ISCIII), 28029 Madrid, Spain; 5 Institute of Health Research Pere Virgili (IISPV), Tarragona, 43003 Spain; 6 Department of Morphological Sciences, CiMUS, University of Santiago de Compostela, 15782 Santiago de Compostela, Spain; 7 CIBER Enfermedades Neurodegenerativas (CIBERNED), 15782 Santiago de Compostela, Spain; 8 University of Bordeaux, INSERM, Neurocentre Magendie, U1215, Bordeaux, F-33000 France; 9 Centro Nacional de Investigaciones Cardiovasculares (CNIC), Madrid, 28029 Spain; 10 CIBER de Enfermedades Cardiovasculares (CIBERCV), Instituto de Salud Carlos III (ISCIII), 28029 Madrid, Spain; 11 Service of Ophthalmology, Complejo Hospitalario Universitario de Santiago de Compostela and CiMUS, University of Santiago de Compostela, Santiago de Compostela, 15782 Spain; 12 Department of Electronic Engineering, Universitat Rovira i Virgili, IISPV, Tarragona, 43007 Spain; 13 Organ Crosstalk in Metabolic Diseases Group, Molecular Oncology Program, Spanish National Cancer Centre (CNIO), Madrid, 28029 Spain

**Keywords:** Metabolism, Neuroscience, Signal Transduction

## Abstract

Proopiomelanocortin (POMC) neurons in the arcuate nucleus of the hypothalamus (ARC) play a critical role in energy homeostasis. 5’-adenosine monophosphate-activated protein kinase (AMPK) is a serine/threonine kinase, which acts as the main energy sensor in the cell. The heterotrimeric AMPK results from the combination of a catalytic α subunit (α1, α2) with two regulatory subunits, β (β1, β2) and γ (γ1, γ2 and γ3). Hypothalamic AMPK plays a key role in the control of energy balance, but current evidence shows that the precise combination of its heterotrimeric components determines its physiological action. Our findings show that AMPKα1 ablation in POMC neurons has a sex-dependent protective effect against diet-induced obesity because of increased thermogenesis in brown adipose tissue (BAT) in males, but not in females. At the molecular level, deletion of AMPKα1 in POMC neurons disrupts the ARC phosphoproteome and is associated with ameliorated endoplasmic reticulum (ER) stress and reduced ceramide content. These findings highlight the complex function of the different AMPK subunits in the hypothalamic regulation of energy balance.

## Synopsis

Deletion of AMPK1 in POMC neurons protects male mice against diet-induced obesity by increasing brown adipose tissue thermogenesis and energy expenditure, without affecting food intake. This effect is associated with reduced hypothalamic ER stress and ceramide content, highlighting a sex-specific and subunit-specific role of hypothalamic AMPK in energy balance.

- AMPK1 ablation in POMC neurons protects male mice from diet-induced obesity.
- The metabolic phenotype is driven by increased brown adipose tissue thermogenesis and energy expenditure, not by reduced food intake.
- POMC-specific AMPK1 loss is associated with reduced hypothalamic ER stress and ceramide levels
- The effect is sexually dimorphic, as female mice do not show the same protective phenotype.

## Abstract (web summary)

Deletion of AMPK1 in POMC neurons protects male mice against diet-induced obesity by increasing brown adipose tissue thermogenesis and energy expenditure, without affecting food intake. This effect is associated with reduced hypothalamic ER stress and ceramide content, highlighting a sex-specific and subunit-specific role of hypothalamic AMPK in energy balance.

## Graphical abstract

**Figure:**
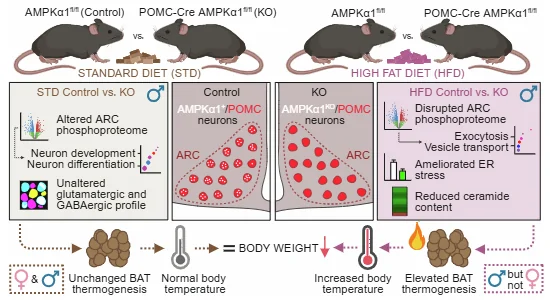

## Introduction

The arcuate nucleus of the hypothalamus (ARC), anatomically located adjacent to the semipermeable blood–brain barrier (BBB) of the median eminence (ME) and surrounding the third ventricle (3 V), is a fundamental area to sense, integrate and respond to circulating hormonal and metabolic signals (Quarta et al, 2021; Quarta et al, 2020; Schneeberger et al, 2014). Within the ARC, two neuronal populations play critical but opposing roles in energy homeostasis. Agouti-related peptide (AgRP) neurons are activated by a negative energy balance and stimulate food intake and reduce energy expenditure by antagonizing melanocortin 4 receptor (MC4R) signaling in second-order neurons (Quarta et al, 2021; Quarta et al, 2020; Schneeberger et al, 2014). In contrast, proopiomelanocortin (POMC) neurons are activated by conditions of high energy availability and release the MC4R agonist α-melanocyte-stimulating hormone (α-MSH) onto second-order neurons to which they send projections (Leon et al, 2024; Quarta et al, 2021; Quarta et al, 2020; Schneeberger et al, 2014). Consequently, POMC neurons induce an anorectic effect and increase energy expenditure to maintain energy homeostasis. Accordingly, both genetic defects and metabolic challenges to the melanocortin system cause impairments, which can lead to the development of obesity (Ramadori et al, 2010; Sweeney et al, 2023; Yeo et al, 2021). Leptin resistance and impaired serotonin responses in POMC and/or AgRP neurons are considered a paradigmatic event in diet-induced obesity (Guo et al, 2019; Ramadori et al, 2010; Sweeney et al, 2023; Yeo et al, 2021). However, the molecular underpinnings causing the malfunction of this neuronal system in obesity are still under investigation.

5’ adenosine monophosphate-activated protein kinase (AMPK) is a serine/threonine kinase that acts as a major energy sensor in the hypothalamus (Lopez, 2022; López et al, 2016; Schneeberger and Claret, 2012), acting as a molecular mediator of several hormones (e.g., leptin, ghrelin, thyroid hormones, estradiol, and BMP8b), and drugs (e.g., nicotine, liraglutide, olanzapine) (Lopez, 2022; López et al, 2016; Schneeberger and Claret, 2012). Composed of a heterotrimeric complex, AMPK results from the combination of a catalytic α (α1 and α2) subunit, with a serine/threonine kinase domain, and two regulatory subunits β (β1 and β2) and γ (γ1, γ2, and γ3), with each subunit encoded by different genes (Lopez, 2022; López et al, 2016; Schneeberger and Claret, 2012). The possible combinations of the heterotrimeric αβγ AMPK complex display a particular pattern of expression depending on the tissue and cellular population considered (Lopez, 2022; López et al, 2016; Schneeberger and Claret, 2012).

The role of AMPK subunits in different neuronal populations has revealed specific regulatory activities and functions (Lopez, 2022; López et al, 2016; Schneeberger and Claret, 2012). Genetically modified mouse lines have helped to precisely dissect those roles. Deletion of AMPKα1 in steroidogenic factor‑1 (SF1) neurons of the ventromedial hypothalamic nucleus (VMH) increases brown adipose tissue (BAT) thermogenesis and energy expenditure, resulting in a lean phenotype resistant to diet‑induced obesity (Martinez-Sanchez et al, 2017b; Seoane-Collazo et al, 2018). Consistent with this rationale, exosome‑mediated targeting of AMPKα1 in SF1 neurons also represents an effective anti‑obesity approach (Milbank et al, 2023; Milbank et al, 2021; Mukherjee et al, 2023). Conversely, loss of AMPKγ2 in SF1 neurons causes obesity associated with reduced BAT thermogenesis (Freire-Agulleiro et al, 2025). Similarly, deletion of AMPKα2 in POMC neurons induces obesity through reduced energy expenditure and elevated food intake, whereas its loss in AgRP neurons leads to an age‑dependent lean phenotype (Claret et al, 2007).

Overall, the current knowledge indicates that a precise and cell-specific study of the different hypothalamic AMPK subunits in the melanocortin system is necessary to understand their function. However, the specific role of AMPKα subunits in mediating neuronal adaptations to changes in energy homeostasis and body weight regulation remains unexplored. This study aimed to elucidate the role of AMPKα1 in hypothalamic POMC neurons in whole-body energy balance regulation. Our results unveil that genetic ablation of AMPKα1 in POMC neurons of the ARC induced a feeding-independent protection against diet-induced obesity coupled to an increased BAT thermogenesis in male, but not in female mice. This phenotype was linked to phosphoproteome alterations and reduced hypothalamic ER stress and ceramide content, highlighting the cell-specific roles of AMPK subunits in the metabolic adaptation to obesogenic diets and specifically implicating the α1 subunit in this process.

## Results

### Generation and validation of specific AMPKα1 null mice in POMC neurons

**Figure 1:**
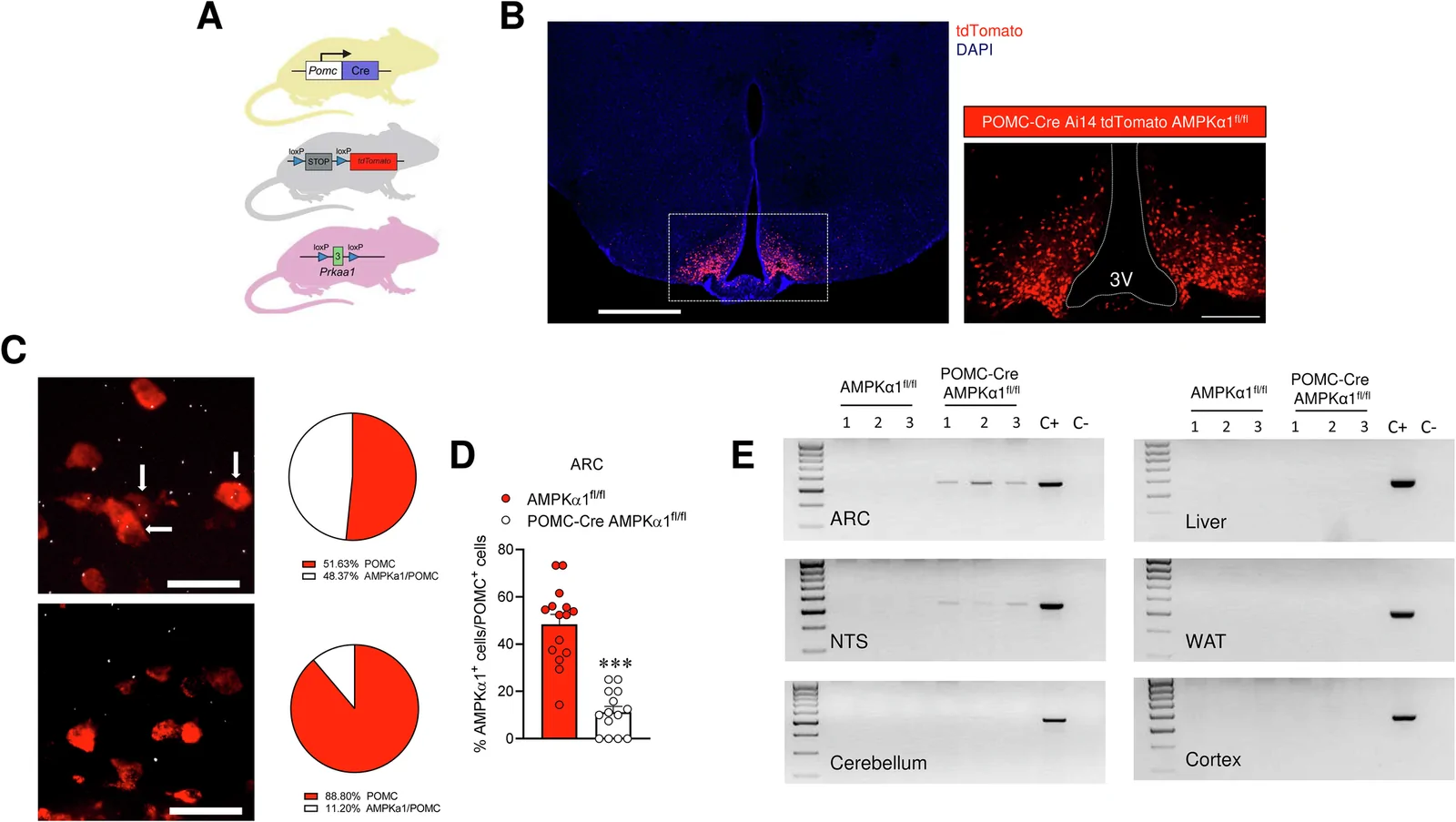
Genetic ablation of AMPKα1 in POMC neurons. (**A**) Graphical scheme showing POMC-Cre tdTomato Ai14 mice and AMPKα1^fl/fl^ mice; *Pomc-*mediated Cre expression (purple) leads to excision of the exon 3 (green) in *Prkaa1* and to the removal of a loxP-STOP-loxP cassette that prevents the expression of tdTomato (red). (**B**) Left: representative tile-scan of confocal images of the hypothalamus depicting ARC-specific tdTomato immunoreactivity (red) of POMC-Cre Ai14-tdTomato AMPKα1^fl/fl^ mice. Scale bar 500 μm. Right: zoomed image of the ARC. Scale bar 150 μm. (**C**) Left: representative images of POMC^+^ neurons (red) and *Prkaa1*-specific probe (white). White arrows indicate colocalization of both signals. Scale bar 50 μm. Right: quantification of the relative number of POMC^+^ positive cells and AMPKα1^+^/POMC^+^ cells. (**D**) Percentage of cells that show colocalization for both POMC and AMPKα1 signal (*n* = 15 brain slices from 4 control mice and 14 brain slices from 4 KO mice). (**E**) PCR for detection of the recombined *Prkaa1* allele in the following tissues from AMPKα1^fl/fl^ and POMC-Cre AMPKα1^fl/fl^ mice: ARC, NTS, cerebellum, liver, WAT, and cortex (*n* = 3 control and 3 KO). C+ and C− indicate positive and negative controls, respectively. Data were expressed as mean ± SEM. Statistical significance was assessed by Student’s *t*-test (**D**). ***P < 0.001 vs. AMPKα1^fl/fl^. Source data are available online for this figure.

To investigate the functional relevance of the AMPKα1 subunit in POMC neurons, we generated a constitutive *Prkaa1* knockout (KO) mouse model in those neurons (referred to as POMC-Cre AMPKα1^fl/fl^). By crossing the POMC-Cre AMPKα1^fl/fl^ mice with the Ai14-tdTomato reporter mouse line (Fig. 1A), we fluorescently labeled the Cre-recombined cells (Fig. 1B), allowing us to confirm the genetic targeting of POMC-derived cells in the ARC. Using the POMC-Cre AMPKα1^fl/fl^ mouse, we detected by double in situ hybridization a clear decrease of *Prkaa1* transcripts in the POMC-derived cells (Fig. 1C,D), validating the knockout of *Prkaa1* in our mouse model. Together with this, we explored the presence of DNA recombination as a surrogate marker of the *Prkaa1* deletion in different tissues of the central nervous system and periphery. We detected amplicons compatible with effective recombination in the ARC, and to a lesser extent, in the nucleus tractus solitarius (NTS) (Fig. 1E). In contrast, non-expressing POMC tissues/areas, such as the cerebellum, cortex, liver, and white adipose tissue (WAT), showed a complete absence of the excised DNA material (Fig. 1E). These results confirm the AMPKα1 subunit deletion in POMC-derived neurons.

### Loss of AMPKα1 in POMC neurons did not impact energy balance under a chow diet

**Figure 2:**
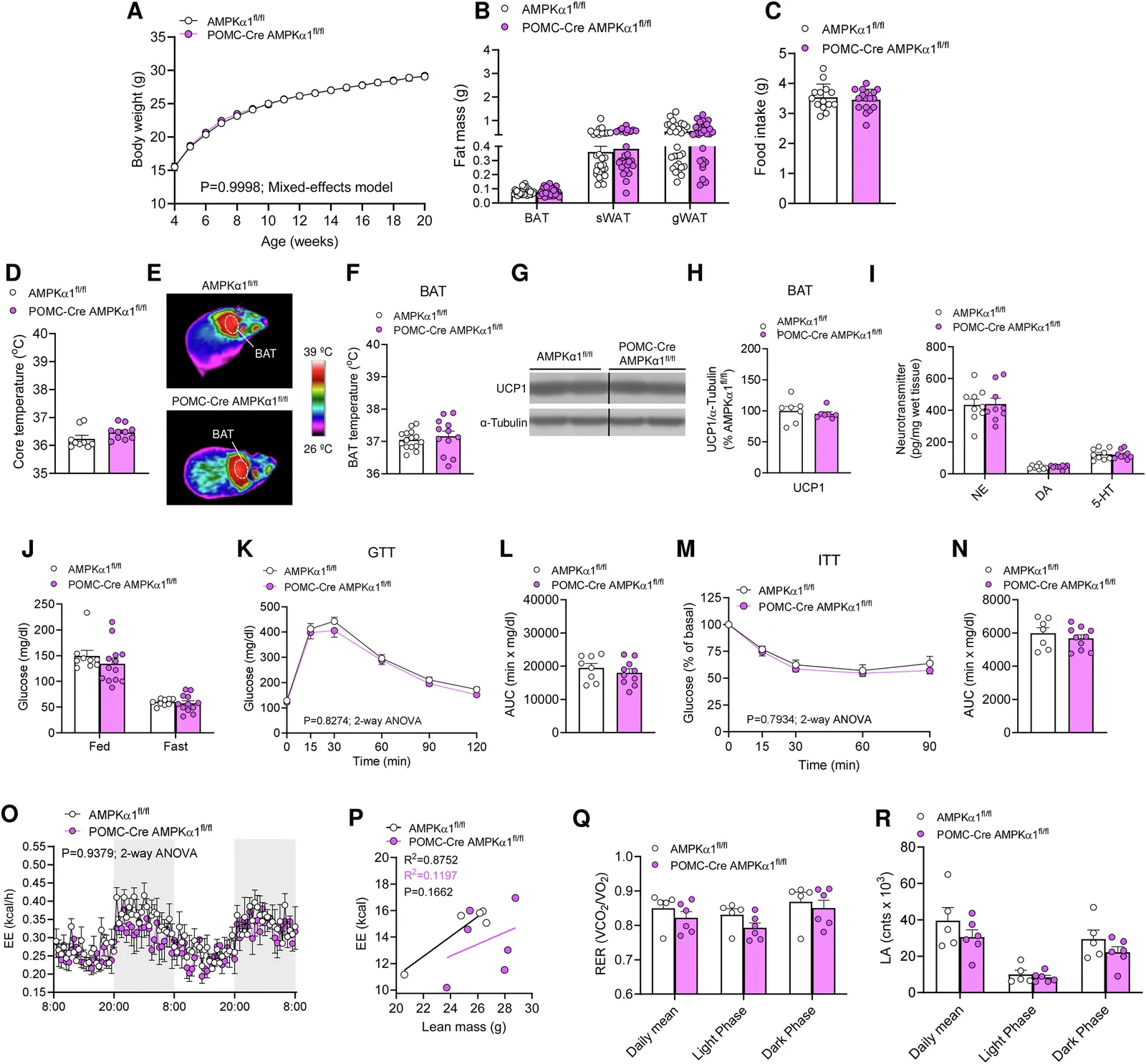
Effects of loss of AMPKα1 in POMC neurons on energy balance, energy expenditure and glucose homeostasis in male mice fed a chow diet. (**A**–**D**) Body weight (**A**; *n* = 33 control and 37 KO), fat depot mass (**B**; *n* = 30 control and 30 KO), daily food intake (**C**; *n* = 14 control and 17 KO), and core body temperature (**D**; *n* = 9 control and 10 KO) of control (littermates AMPKα1^fl/fl^) and POMC-Cre AMPKα1^fl/fl^ male mice. (**E**–**H**) Representative BAT infrared thermographic images (**E**), BAT temperature (**F**; *n* = 15 control and 12 KO), representative BAT UCP1 western blot images (**G**) and BAT UCP1 protein levels (**H**; *n* = 7 control and 7 KO) of control (littermates AMPKα1^fl/fl^) and POMC-Cre AMPKα1^fl/fl^ male mice. In the western blot analyses, α-tubulin was used as a control. A black line between the immunoblots depicts samples loaded in the same gel, but not next to each other. (**I**) BAT neurotransmitter levels (*n* = 9 control and 9 KO) of control (littermates AMPKα1^fl/fl^) and POMC-Cre AMPKα1^fl/fl^ male mice. (**J**–**N**) Fed and fasted blood glucose levels (**J**; *n* = 9 control and 12-13 KO), glucose tolerance test and area under the curve (AUC) (**K**,** L**; *n* = 8 control and 10 KO), insulin tolerance test, and AUC (**M**,** N**; *n* = 7 control and 10 KO) of control (littermates AMPKα1^fl/fl^) and POMC-Cre AMPKα1^fl/fl^ male mice. (**O**–**R**) Energy expenditure (EE; **O**; *n* = 5 control and 6 KO), ANCOVA analysis of 48 h total energy expenditure using lean mass as a covariate (**P**; *n* = 5 control and 6 KO), respiratory exchange ratio (RER; **Q**; *n* = 5 control and 6 KO), locomotor activity (LA; **R**; *n* = 5 control and 6 KO) of control (littermates AMPKα1^fl/fl^) and POMC-Cre AMPKα1^fl/fl^ male mice. Data were expressed as mean ± SEM. Statistical significance was assessed by mixed-effect model (**A**), two-way ANOVA (**K**,** M**, **O**) and Student’s *t*-test (**B**–**D**,** F**,** H**–**J**,** L**,** N**,** Q**,** R**) and ANCOVA (**P**). Source data are available online for this figure.

**Figure EV1:**
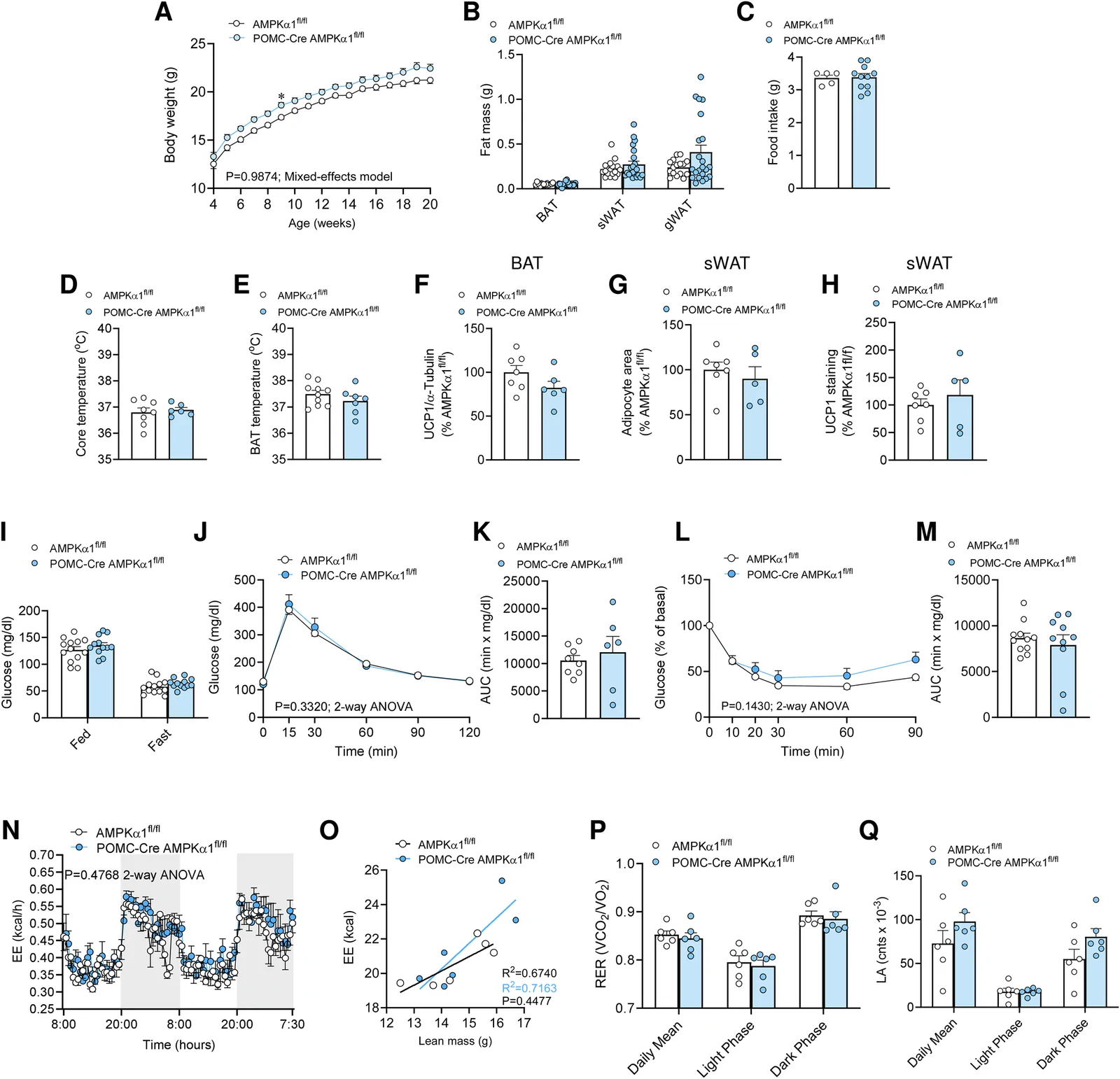
Effects of loss of AMPKα1 in POMC neurons on energy balance, energy expenditure and glucose homeostasis in female mice fed a chow diet. (**A**–**E**) Body weight (**A**; *n* = 20 control and 31 KO), fat depots mass (**B**; *n* = 17 control and 22 KO), and daily food intake (**C**; *n* = 5 control and 11 KO) of control (littermates AMPKα1^fl/fl^) and POMC-Cre AMPKα1^fl/fl^ female mice. (**D**–**H**) Core body temperature (**D**; *n* = 8 control and 6 KO) BAT temperature (**E**; *n* = 10 control and 7 KO), BAT UCP1 protein levels (**F**; *n* = 7 control and 6 KO), quantification of adipocyte area (**G**; *n* = 7 control and 5 KO) and quantification of UCP1-stained area (**H**; *n* = 7 control and 5 KO) of control (littermates AMPKα1^fl/fl^) and POMC-Cre AMPKα1^fl/fl^ female mice. (**I**–**M**) Fed and fasted blood glucose levels (**I**; *n* = 13 control and 12KO), glucose tolerance test and area under the curve (AUC) (**J**, **K**; *n* = 8 control and 6 KO), insulin tolerance test, and AUC (**L**, **M**; *n* = 11 control and 9 KO) of control (littermates AMPKα1^fl/fl^) and POMC-Cre AMPKα1^fl/fl^ female mice. (**N**–**Q**) Energy expenditure (EE; **N**; *n* = 6 control and 6 KO), ANCOVA analysis of 48 h total energy expenditure using body weight as a covariate (**O**; *n* = 6 control and 6 KO), respiratory exchange ratio (RER; **P**; *n* = 6 control and 6 KO), locomotor activity (LA; **Q**; *n* = 6 control and 6 KO) of control (littermates AMPKα1^fl/fl^) and POMC-Cre AMPKα1^fl/fl^ female mice. Data were expressed as mean ± SEM. Statistical significance was assessed by mixed-effect model (**A**), two-way ANOVA (**J**, **L**, **N**) and Student’s *t*-test (**B**–**I**, **K**, **M**, **P**, **Q**) and ANCOVA (**O**). *P < 0.05 vs. female AMPKα1^fl/fl^.

**Figure EV2:**
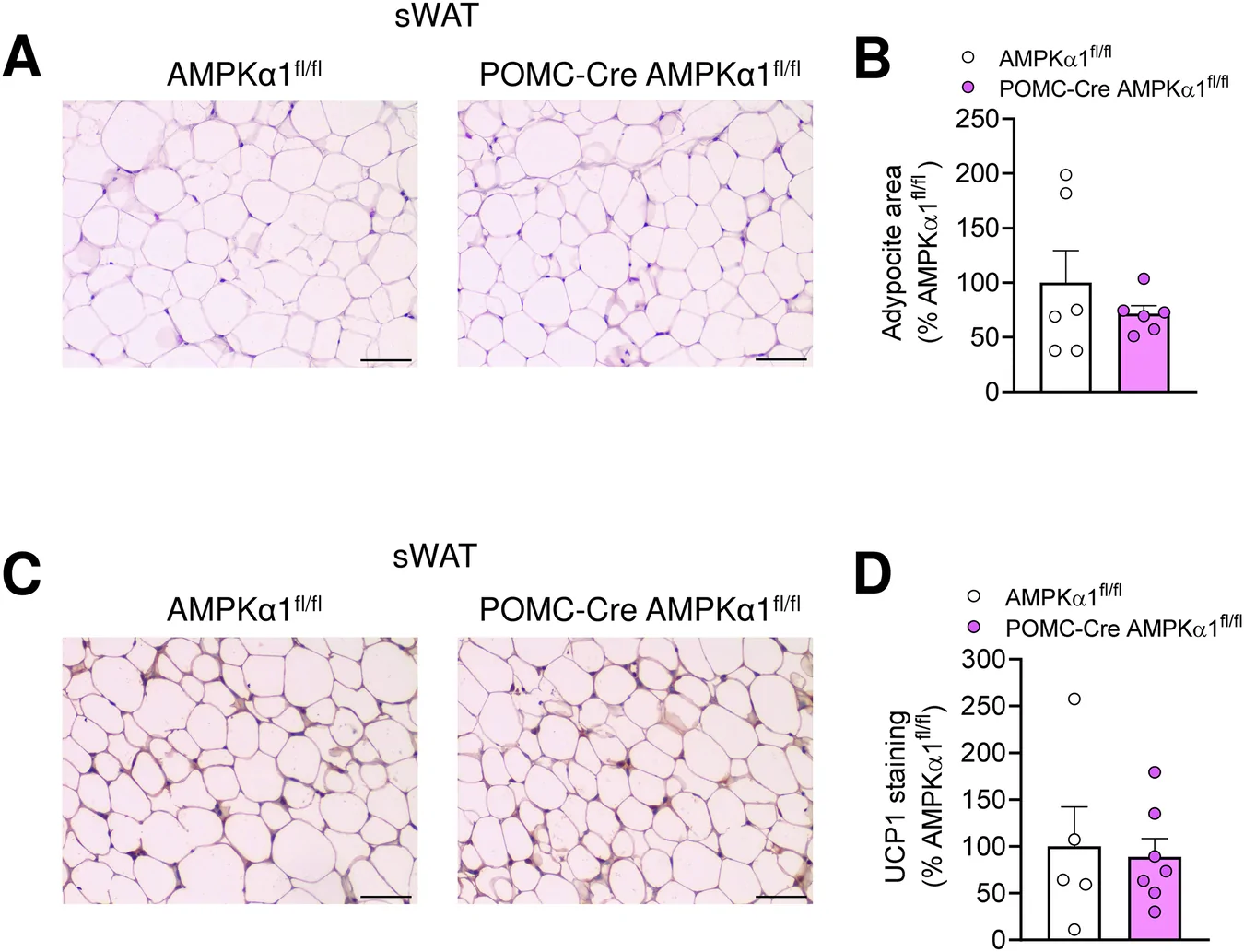
Effects of loss of AMPKα1 in POMC neurons on WAT in male mice fed a chow diet. (**A**,** B**) Quantification of the adipocyte area (**A**), and representative subcutaneous white adipose tissue (sWAT) hematoxylin-eosin staining (**B**; *n* = 6 control and 6 KO) of AMPKα1^fl/fl^ mice, and POMC-Cre AMPKα1^fl/fl^ mice. Scale bar 50 μm. The mean value of each animal was obtained from 3 photos of each tissue. (**C**,** D**) Quantification of UCP1-stained area (**C**) and representative sWAT immunohistochemistry with anti-UCP1 antibody showing UCP1 staining (**D**; *n* = 5 control and 7 KO) of AMPKα1^fl/fl^ mice, and POMC-Cre AMPKα1^fl/fl^ mice. Scale bar 50 μm. Data were expressed as mean ± SEM. Statistical significance was assessed by Student’s *t*-test (**B**,** D**).

To elucidate the metabolic implications of the AMPKα1 subunit ablation in POMC neurons, we subjected male and female POMC-Cre AMPKα1^fl/fl^ mice, including cohorts phenotyped in parallel under standardized conditions, and their littermate controls to a complete metabolic phenotyping under a standard diet. Our data revealed that male and female POMC-Cre AMPKα1^fl/fl^ mice attained the same body weight as the controls when fed chow (Figs. 2A and EV1A). The weight of fat pads (Figs. 2B and EV1B) and average daily food intake (Figs. 2C and EV1C) were similar between POMC-Cre AMPKα1^fl/fl^ mice and controls. We also observed comparable body (Figs. 2D and EV1D) and BAT temperature (Figs. 2E,F and EV1E), as along with similar UCP1 levels in the BAT between genotypes (Figs. 2G,H and EV1F) and norepinephrine (NE, indicative of similar levels of sympathetic BAT activation), dopamine (DA) and serotonin (5-HT) levels (Fig. 2I). Both male and female controls and POMC-Cre AMPKα1^fl/fl^ mice also exhibited similar levels of subcutaneous WAT (sWAT) browning, assessed by adipocyte area (Figs. EV1G and EV2A,B) and UCP1 staining (Figs. EV1H and EV2C,D).

Given the relevance of POMC neurons in the regulation of glucose homeostasis (Quarta et al, 2021; Quarta et al, 2020; Schneeberger et al, 2014), we characterized blood glucose levels in fed and fasted states (Figs. 2J and EV1I), glucose tolerance (Figs. 2K,L and EV1J,K) and insulin sensitivity (Figs. 2M,N and EV1L,M) of POMC-Cre AMPKα1^fl/fl^ mice and controls without observing differences between genotypes either in males or females. To complete the metabolic phenotyping of these animals, we subjected them to indirect calorimetry. POMC-Cre AMPKα1^fl/fl^ mice and controls displayed similar energy expenditure (EE; Figs. 2O,P and EV1N,O), respiratory exchange ratio (RER; Figs. 2Q and EV1P) and locomotor activity (LA; Figs. 2R and EV1Q). Overall, this evidence suggested that the lack of AMPKα1 in POMC neurons did not impact energy metabolism in conditions of a standard diet in either male or female mice.

### Loss of AMPKα1 in POMC neurons conferred protection against HFD in male but not female mice

**Figure 3:**
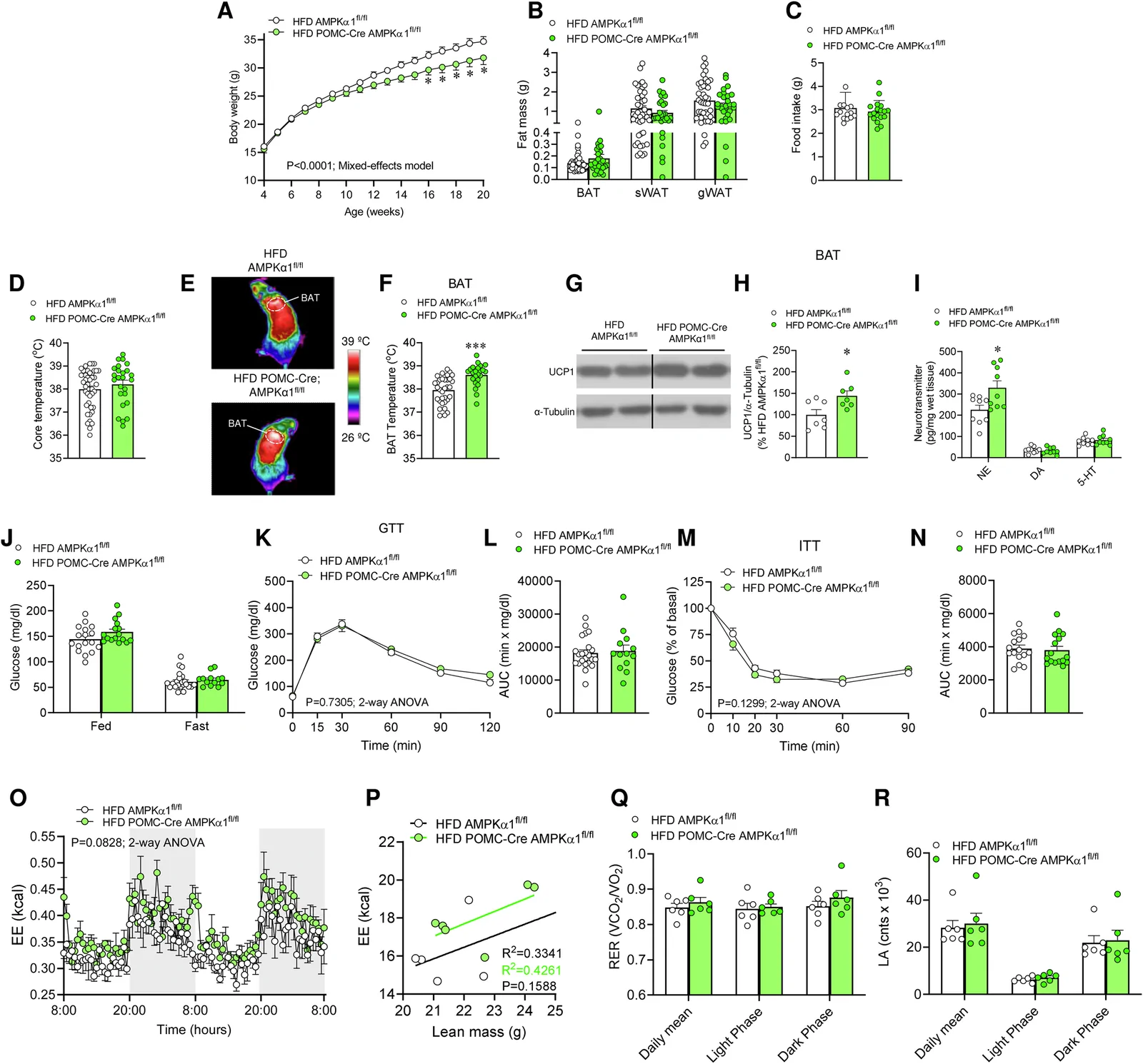
Effects of loss of AMPKα1 in POMC neurons on energy balance, energy expenditure and glucose homeostasis in male mice fed a HFD. (**A**–**D**) Body weight (**A**; *n* = 44 control and 31 KO), fat depot mass (**B**; *n* = 43 control and 29 KO), daily food intake (**C**; *n* = 15 control and 18 KO), core body temperature (**D**; *n* = 43 control and 25 KO) of control (littermates AMPKα1^fl/fl^) and POMC-Cre AMPKα1^fl/fl^ male mice fed a HFD. (**E**–**H**) Representative BAT infrared thermographic images (**E**), BAT temperature (**F**; *n* = 33 control and 23 KO; *P* < 0.0001), representative BAT UCP1 western blot images (**G**) and BAT UCP1 protein levels (**H**; *n* = 7 control and 6 KO; *P* = 0.0212) of control (littermates AMPKα1^fl/fl^) and POMC-Cre AMPKα1^fl/fl^ male mice fed a HFD. In the western blot analyses, α-tubulin was used as a control. A black line between the immunoblots depicts samples loaded in the same gel, but not next to each other. (**I**) BAT neurotransmitter levels (*n* = 9 control and 9 KO; *P* = 0.017622 for NE) of control (littermates AMPKα1^fl/fl^) and POMC-Cre AMPKα1^fl/fl^ male mice fed a HFD. (**J**–**N**) Fed and fasted blood glucose levels (**J**; *n* = 17–23 control and 13–16 KO), glucose tolerance test and area under the curve (AUC) (**K**,** L**; *n* = 23 control and 13 KO), insulin tolerance test, and AUC (**M**,** N**; *n* = 16 control and 16 KO) of control (littermates AMPKα1^fl/fl^) and POMC-Cre AMPKα1^fl/fl^ male mice fed a HFD. (**O**–**R**) Energy expenditure (EE; **O**; *n* = 6 control and 6 KO), ANCOVA analysis of 48 h total energy expenditure using lean mass as a covariate (**P**; *n* = 6 control and 6 KO), respiratory exchange ratio (RER; **Q**; *n* = 6 control and 6 KO), locomotor activity (LA; **R**; *n* = 6 control and 6 KO) of control (littermates AMPKα1^fl/fl^) and POMC-Cre AMPKα1^fl/fl^ male mice fed a HFD. Data were expressed as mean ± SEM. Statistical significance was assessed by mixed-effect model (**A**), two-way ANOVA (**K**,** M**,** O**) and Student’s *t*-test (**B**–**D**,** F**,** H**–**J**,** L**,** N**,** Q**,** R**) and ANCOVA (**P**). Source data are available online for this figure.

**Figure EV3:**
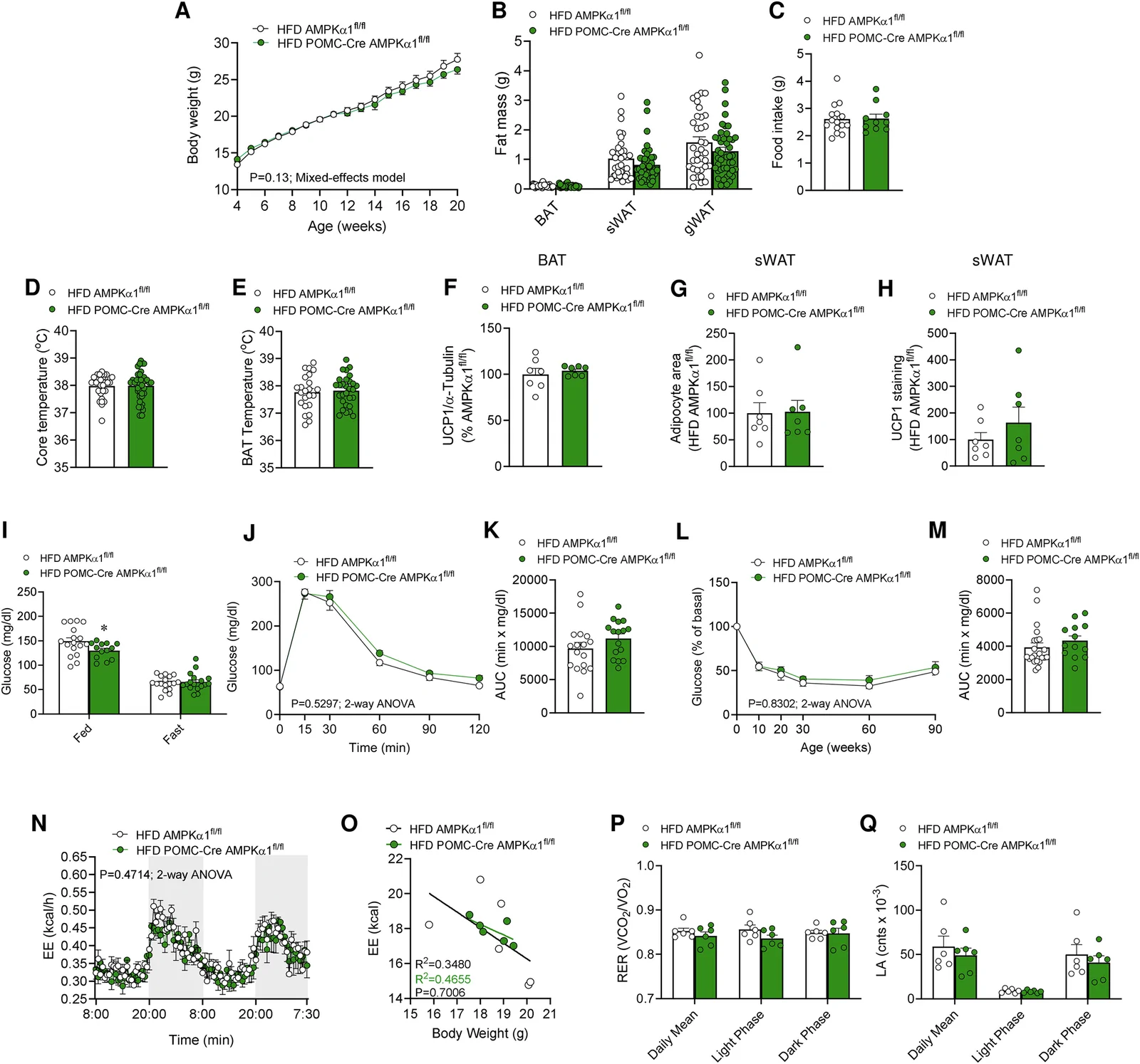
Effects of loss of AMPKα1 in POMC neurons on energy balance, energy expenditure and glucose homeostasis in female mice fed a HFD. (**A**–**E**) Body weight (**A**; *n* = 31 control and 42 KO), fat depot mass (**B**; *n* = 36 control and 43 KO), and daily food intake (**C**; *n* = 15 control and 10 KO) of control (littermates AMPKα1^fl/fl^) and POMC-Cre AMPKα1^fl/fl^ female mice in HFD. (**D**–**H**) Core body temperature (**D**; *n* = 28 control and 38 KO) BAT temperature (**E**; *n* = 23 control and 29 KO), BAT UCP1 protein levels (**F**; *n* = 7 control and 7 KO), quantification of adipocyte area (**G**; *n* = 7 control and 7 KO) and quantification of UCP1-stained area (**H**; *n* = 7 control and 7 KO) of control (littermates AMPKα1^fl/fl^) and POMC-Cre AMPKα1^fl/fl^ female mice in HFD. (**I**–**M**) Fed and fasted blood glucose levels (**I**; *n* = 17 control and 13-17 KO; *P* = 0.047664 for fed), glucose tolerance test and area under the curve (AUC) (**J**, **K**; *n* = 17 control and 16 KO), insulin tolerance test, and area under the curve (**L**, **M**; *n* = 22 control and 13 KO) of control (littermates AMPKα1^fl/fl^) and POMC-Cre AMPKα1^fl/fl^ female mice in HFD. (**N**–**Q**) Energy expenditure (EE; **N**; *n* = 6 control and 6 KO), ANCOVA analysis of 48 h total energy expenditure using body weight as a covariate (**O**; *n* = 6 control and 6 KO), respiratory exchange ratio (RER; **P**; *n* = 6 control and 6 KO), locomotor activity (LA; **Q**; *n* = 6 control and 6 KO) of control (littermates AMPKα1^fl/fl^) and POMC-Cre AMPKα1^fl/fl^ female mice in HFD. Data are expressed as mean ± SEM. Statistical significance was assessed by Mixed-effect model (**A**), two-way ANOVA (**J**, **L**, **N**), Student’s *t*-test (**B**–**I**,** K**,** M**,** P**,** Q**) and ANCOVA (**O**). *P < 0.05 vs. HFD female AMPKα1^fl/fl^.

**Figure EV4:**
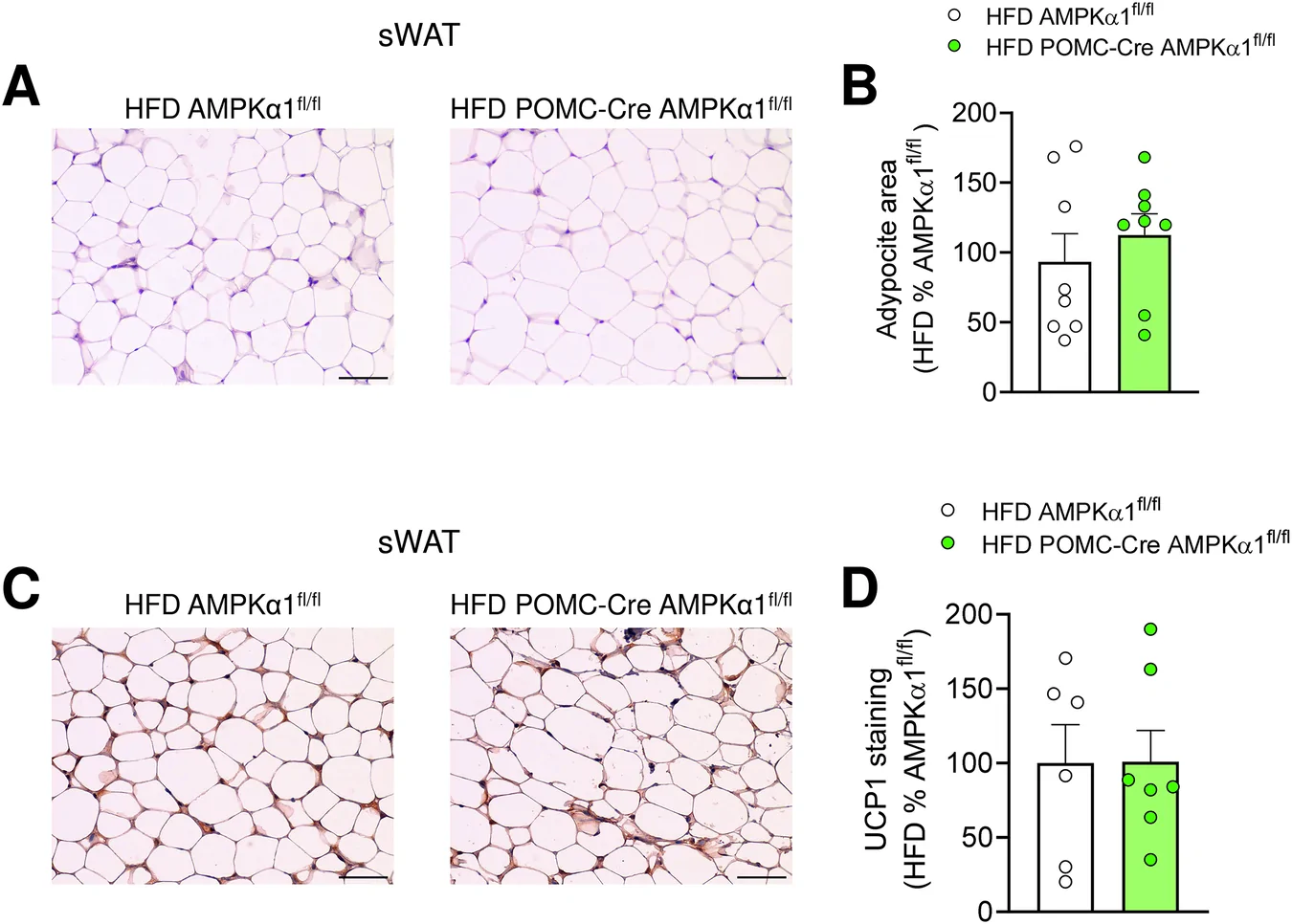
Effects of loss of AMPKα1 in POMC neurons on WAT in male mice fed a HFD. (**A**,** B**) Quantification of the adipocyte area (**A**), and representative subcutaneous white adipose tissue (sWAT) hematoxylin-eosin staining (**B**; *n* = 8 control and 8 KO) of AMPKα1^fl/fl^ mice, and POMC-Cre AMPKα1^fl/fl^ mice fed a HFD. Scale bar 50 μm. The mean value of each animal was obtained from three photos of each tissue. (**C**,** D**) Quantification of UCP1-stained area (**C**) and representative sWAT immunohistochemistry with anti-UCP1 antibody showing UCP1 staining (**D**; *n* = 7 control and 6 KO) of AMPKα1^fl/fl^ mice, and POMC-Cre AMPKα1^fl/fl^ mice fed a HFD. Scale bar 50 μm. Data are expressed as mean ± SEM. Statistical significance was assessed by Student’s *t*-test (**B**,** D**).

To gain further insights into the role of the AMPKα1 subunit in POMC neurons in the metabolic context of obesity, we characterized male and female POMC-Cre AMPKα1^fl/fl^ mice and their controls under a high-fat diet (HFD). Several reports have shown how POMC neurons participate in the pathophysiological mechanisms of diet-induced obesity (Claret et al, 2007; Quarta et al, 2021; Quarta et al, 2020; Ramadori et al, 2010; Souza et al, 2016). We observed that male, but not female, POMC-Cre AMPKα1^fl/fl^ mice were protected against the body weight gain induced by HFD (Figs. 3A and EV3A). However, fat pads weight did not show major differences in either male or female POMC-Cre AMPKα1^fl/fl^ mice (Figs. 3B and EV3B). When the different components of the energy balance were dissected, we observed no differences in either food intake (Figs. 3C and EV3C), or body temperature (Figs. 3D and EV3D). On the other hand, in males, but not females, BAT displayed an increased temperature (Figs. 3E,F and EV3E) and UCP1 expression (Figs. 3G,H and EV3F). In line with these data, NE levels were increased in the BAT of POMC-Cre AMPKα1^fl/fl^ male mice (Fig. 3I), indicating enhanced sympathetic activation of the tissue. Of note, the thermogenic effect seemed restricted to the BAT since null mice and their control littermates displayed similar levels of sWAT browning, with no differences in adipocyte area (Figs. EV3G and EV4A,B) or UCP1 staining Figs. EV3H and EV4C,D).

In keeping with the similar food intake observed between POMC-Cre AMPKα1^fl/fl^ mice and controls fed a HFD, we detected no changes in glycemia in both fed and fasted conditions (Figs. 3J and EV3I). Glucose tolerance (Figs. 3K,L and EV3J,K), and insulin sensitivity (Figs. 3M,N and EV3L,M) were similar in POMC-Cre AMPKα1^fl/fl^ male and female mice fed a HFD, when compared to control littermates. The observed changes in thermogenesis, including higher BAT temperature and UCP1 expression in POMC-Cre AMPKα1^fl/fl^ male mice, led us to hypothesize that components of overall energy homeostasis would be affected. Indirect calorimetry analyses demonstrated that POMC-Cre AMPKα1^fl/fl^ male, but not female, mice fed a HFD displayed a tendency toward higher EE when compared to controls (Figs. 3O,P and EV3N,O). However, both male and female mice presented similar RER (Figs. 3Q and EV3P) and LA (Figs. 3R and EV3Q) between genotypes. Overall, these results point that absence of AMPKα1 in POMC neurons has a protective effect under obesogenic conditions in male, but not female mice, and the reduction in body weight is driven by an increased BAT thermogenesis and EE. This sex-specific HFD response indicates that AMPKα1 in POMC neurons exerts diet-dependent, sexually dimorphic control over energy balance, likely reflecting gonadal hormone modulation of hypothalamic energy sensing.

### Loss of AMPKα1 in POMC neurons did not impact either cold- or diet-induced thermogenesis

**Figure 4:**
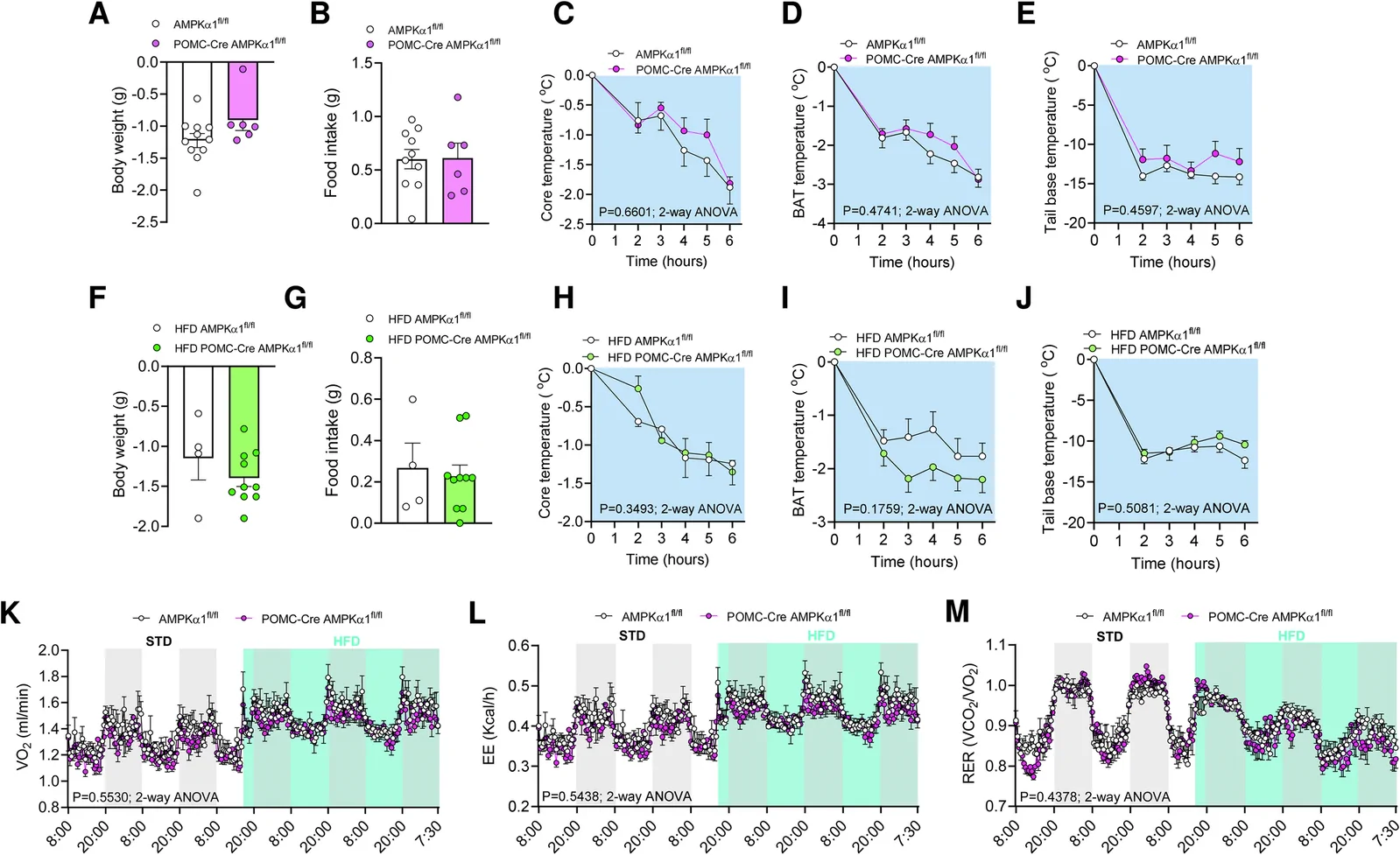
Effects of loss of AMPKα1 in POMC neurons in cold and short-term dietary responses in male mice. (**A**–**E**) Body weight (**A**; *n* = 11 control and 6 KO), food intake (**B**; *n* = 11 control and 6 KO), change in: core body temperature (**C**; *n* = 10 control and 6 KO), BAT temperature (**D**; *n* = 10 control and 6 KO) and tail base temperature (**E**; *n* = 10 control and 6 KO) of cold exposed control (littermates AMPKα1^fl/fl^) and POMC-Cre AMPKα1^fl/fl^ male mice. (**F**–**J**) Body weight (**F**; *n* = 4 control and 10 KO), food intake (**G**; *n* = 4 control and 9 KO), change in: core body temperature (**H**; *n* = 4 control and 10 KO), BAT temperature (**I**; *n* = 4 control and 10 KO) and tail base temperature (**J**; *n* = 4 control and 10 KO) of cold exposed control (littermates AMPKα1^fl/fl^) and POMC-Cre AMPKα1^fl/fl^ male mice fed a HFD. (**K**–**M**) Oxygen consumption rate (VO_2_; **K**; *n* = 6 control and 6 KO), energy expenditure (EE; **L**; *n* = 6 control and 6 KO), and respiratory exchange ratio (RER; **M**; *n* = 6 control and 6 KO) of control (littermates AMPKα1^fl/fl^) and POMC-Cre AMPKα1^fl/fl^ male mice, during a chow-HFD switch. Cold exposure and diet switch experiments were performed once. Data were expressed as mean ± SEM. Statistical significance was assessed by two-way ANOVA (**C**–**E**,** H**–**M**) and Student’s *t*-test (**A**,** B**,** F**,** G**). Source data are available online for this figure.

To determine whether the elevated basal BAT activity in POMC AMPKα1 KO mice reflects enhanced adaptive thermogenic capacity, we challenged male POMC-Cre AMPKα1^fl/fl^ mice and their littermate controls with acute cold exposure (4 °C for 6 h) under both chow and HFD conditions. Neither body weight (Fig. 4A) nor food intake (Fig. 4B) was affected by cold challenge in chow mice. Core body temperature maintenance was identical between genotypes during cold challenge in chow-fed mice (Fig. 4C), accompanied by comparable intrascapular BAT temperature dynamics (Fig. 4D) and tail temperature as a vasoconstriction/heat dissipation proxy (Fig. 4E). These findings were replicated in HFD-fed mice, with no differences in body weight (Fig. 4F), food intake (Fig. 4G), core body temperature (Fig. 4H), BAT temperature kinetics (Fig. 4I), and tail temperature (Fig. 4J). Overall, cold tolerance outcomes confirmed preserved adaptive responses across diets in null mice. We further assessed metabolic adaptation to dietary challenge through indirect calorimetry during the chow-to-HFD transition. Oxygen consumption rates (VO_2_; Fig. 4K) and EE dynamics (Fig. 4L) were similar between genotypes across light/dark cycles. RER declined equivalently in both groups, reflecting a similar shift toward fat oxidation upon HFD introduction (Fig. 4M). Collectively, these data demonstrate that POMC AMPKα1 deletion elevates basal sympathetic nervous system (SNS)-driven BAT tone without altering cold- or diet-induced thermogenic adaptations. Notably, this uncouples POMC AMPKα1 from the dual basal/cold-adaptive role well-established for SF1 neuronal AMPKα1 (Martinez-Sanchez et al, 2017b; Milbank et al, 2021; Seoane-Collazo et al, 2018), highlighting hypothalamic neuronal subunit specificity.

### Loss of AMPKα1 in POMC neurons impacted the ARC phosphoproteome

**Figure 5:**
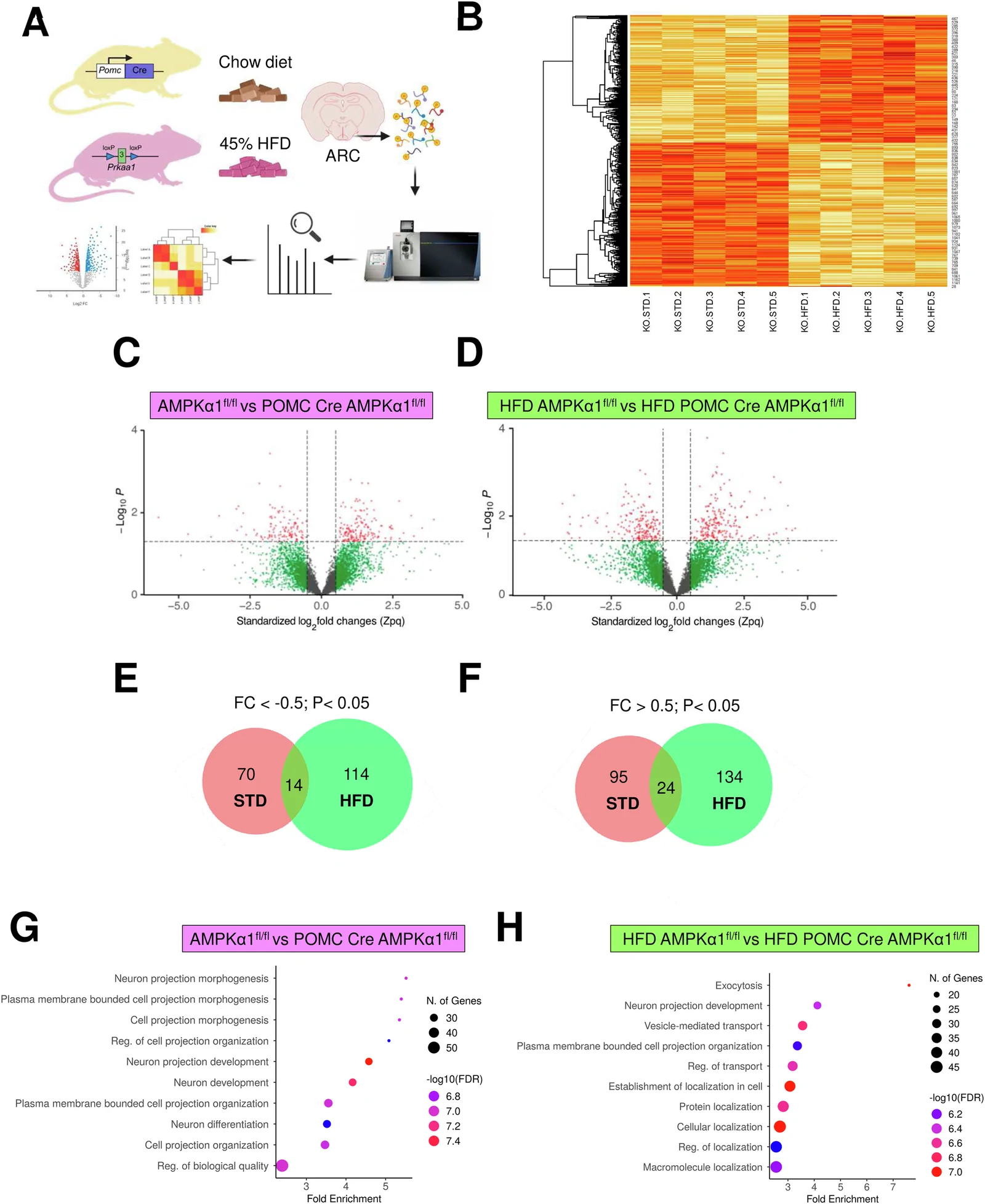
Impact of AMPKα1 deletion in POMC neurons on the phosphoproteome of the ARC. (**A**) Schematic representation of the phosphoproteomics workflow. (**B**) Hierarchical clustering of differentially phosphorylated peptides in the ARC of POMC-Cre AMPKα1^fl/fl^ male mice under standard (STD) or high-fat diet (HFD). Relative abundances of phosphopeptides were expressed in relation to their corresponding protein using standardized log2-ratios (*Zpq* values). (**C**) Volcano plot illustrating differential phosphorylation in the ARC [*n* = 5 control (ten total mice pulled by pairs) and 5 KO (ten total mice pulled by pairs)] between AMPKα1^fl/fl^ and POMC-Cre AMPKα1^fl/fl^ male mice. (**D**) Volcano plot depicting differential phosphorylation in the ARC [*n* = 5 control (ten total mice pulled by pairs) and five KO (ten total mice pulled by pairs)] between HFD AMPKα1^fl/fl^ and HFD POMC-Cre AMPKα1^fl/fl^ male mice. The phosphopeptides with the highest abundance changes (*P* < 0.05 and *Zpq* > 0.5 or <−0.5) in (**C**) and in (**D**) are highlighted in red. (**E**,** F**) Venn diagram showing the overlap between downregulated (**E**) and upregulated (**F**) phosphopeptides with the highest abundance change in STD POMC-Cre AMPKα1^fl/fl^ and HFD POMC-Cre AMPKα1^fl/fl^ male mice. (**G**) Bubble plot of the top ten enriched GO Biological Process terms associated with the proteins harboring the phosphopeptides selected in the comparison between AMPKα1^fl/fl^ and POMC-Cre AMPKα1^fl/fl^ male mice. (**H**) Bubble plot of the top ten enriched GO Biological Process terms for phosphopeptide-harboring proteins in HFD-fed AMPKα1^fl/fl^ and POMC-Cre AMPKα1^fl/fl^ male mice. In (**F** and **G**), circle size reflects the number of proteins, and color intensity indicates enrichment significance (−log10(FDR)). Statistical significance was assessed by Student’s *t*-test (**C**,** D**). Source data are available online for this figure.

**Figure 6:**
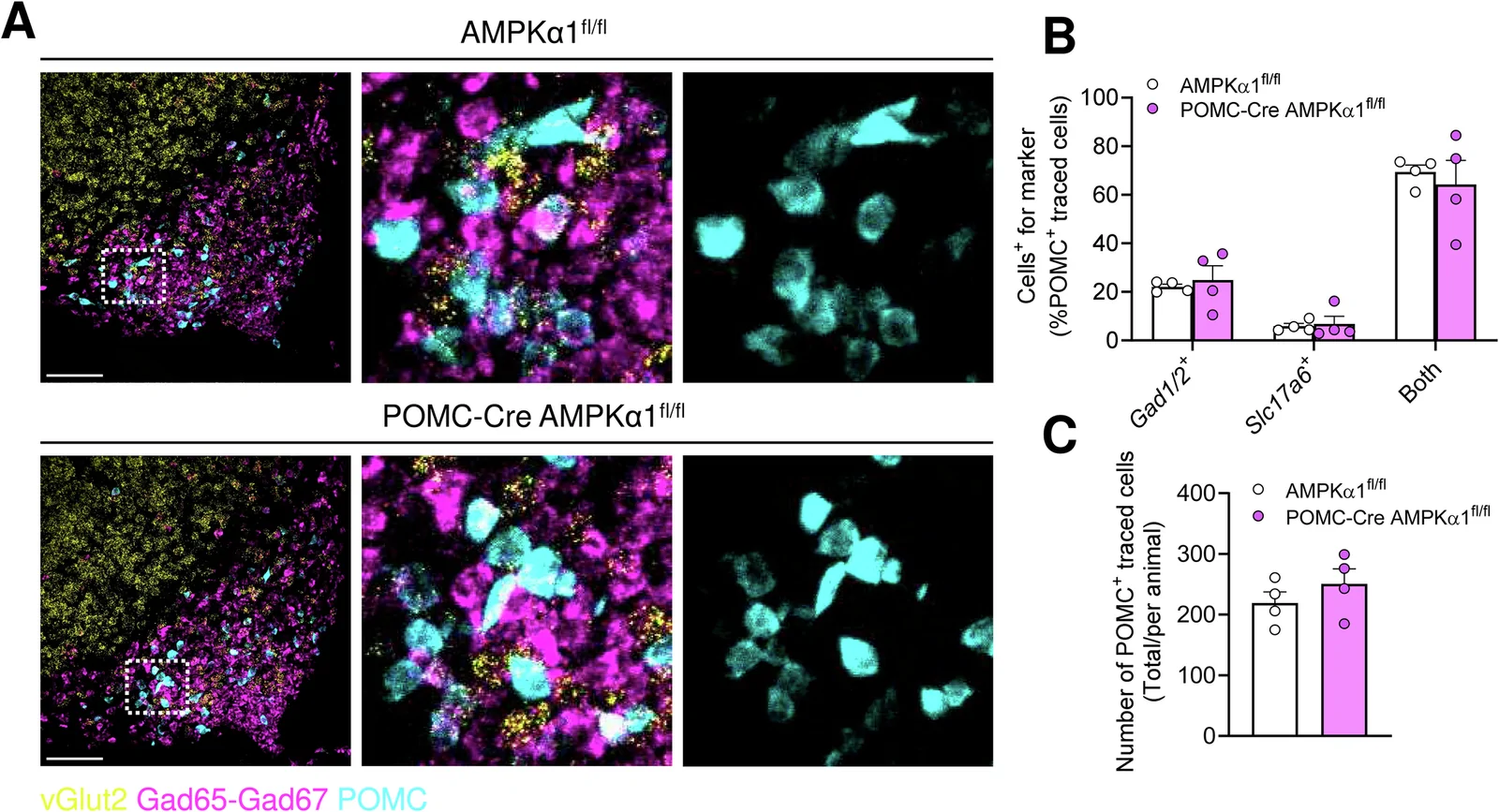
Effect of loss of AMPKα1 in POMC neurons on their neurotransmitter profile in male mice fed a HFD. (**A**) Top: representative confocal images from the ARC of AMPKα1^fl/fl^ mice; bottom: representative confocal images from the ARC of POMC-Cre AMPKα1^fl/fl^ mice. Both, showing GABAergic (Gad65 (*Gad2*) and Gad67 (*Gad1*) in pink), and glutamatergic (vGlut2 (*Slc17a6*) in yellow) profile of POMC neurons (in blue). Scale bar 100 μm. Zoomed images come from the squared region in the first image, depicting details of the POMC neurons neurotransmitter profile. (**B**) Quantification of the number of POMC^+^ neurons that co-express either *Gad1/2* (GABAergic), *Slc17a6* (glutamatergic) or both markers (*n* = 4 control and 4 KO animals). (**C**) Total number of POMC neurons traced from AMPKα1^fl/fl^ mice, and POMC-Cre AMPKα1^fl/fl^ mice (*n* = 4 control and 4 KO animals). Data were expressed as mean ± SEM. Statistical significance was assessed by Student’s *t*-test (**B**,** C**). Source data are available online for this figure.

To investigate the molecular mechanisms potentially underlying our observed thermogenic phenotype, hypothalami were harvested from POMC-Cre AMPKα1^fl/fl^ male mice and littermate controls that were fed either chow or HFD and subjected to unbiased phosphoproteomics (Fig. 5A). To assess the impact of AMPKα1 deletion in POMC neurons, we performed unsupervised clustering of the differentially enriched phosphoproteins. This analysis clearly distinguished POMC-Cre AMPKα1^fl/fl^ mice based on dietary conditions, demonstrating the robustness of our approach to capture metabolic effects on the phosphoproteomic profile (Fig. 5B). To further characterize the molecular signatures associated with the genetic loss of AMPKɑ1 in POMC neurons, we performed differential expression analysis by comparing POMC-Cre AMPKα1^fl/fl^ mice and littermate controls. Under chow diet, AMPKɑ1 deletion in POMC neurons led to a profound alteration in the phosphorylation profile in the ARC, (Fig. 5C). The HFD also produced a clear modification of the phosphorylation pattern when comparing the null mice with the controls (Fig. 5D). However, when comparing between the diets the proteins harboring the affected phosphopeptides (acknowledging that a single protein may harbor multiple phosphorylation at different residues) most of them were of different nature, with few proteins being shared in both diets, suggesting that the phosphoproteome profile is highly dependent on the metabolic context (Fig. 5E,F). The fact that diet alters the pattern of phosphoproteome changes may explain why the phenotype we observed became apparent only under diet-induced obesity. To gain further insight into the downstream molecular effects of AMPKα1 deletion in POMC neurons, particularly under HFD, we performed a post hoc Gene Ontology (GO) enrichment analysis on the non-overlapping phosphopeptide-containing protein sets. Under the chow diet, AMPKα1 loss in POMC neurons enriched biological processes related to neuronal projection, morphogenesis and cell projection organization (Fig. 5G). Interestingly, categories related to neuron development and differentiation were also enriched, raising the possibility that AMPKα1 deletion alters the neurotransmitter identity of POMC neurons. However, the percentage of glutamatergic and GABAergic POMC neurons was similar between genotypes (Fig. 6A,B), as well as the total number of POMC neurons, which was comparable (Fig. 6A,C), thus discarding any differentiation issue of our model. Applying a similar analysis on HFD in the POMC-Cre AMPKα1^fl/fl^ mice, we found that affected phosphoproteins belonged to several biological processes associated with exocytosis and vesicle transport, as well as cell localization. We also found enriched projection-related processes (Fig. 5H). Taken together, these findings showed that AMPKα1 deletion in POMC neurons drives specifically differential phosphorylation landscapes under chow and HFD conditions. Moreover, the enrichment of exocytosis-associated pathways in diet-induced obesity is particularly remarkable, as it may carry significant functional implications given the peptidergic nature of POMC neurons.

### Loss of AMPKα1 in POMC neurons was associated with an amelioration of ER stress and ceramide levels in the ARC in HFD

**Figure 7:**
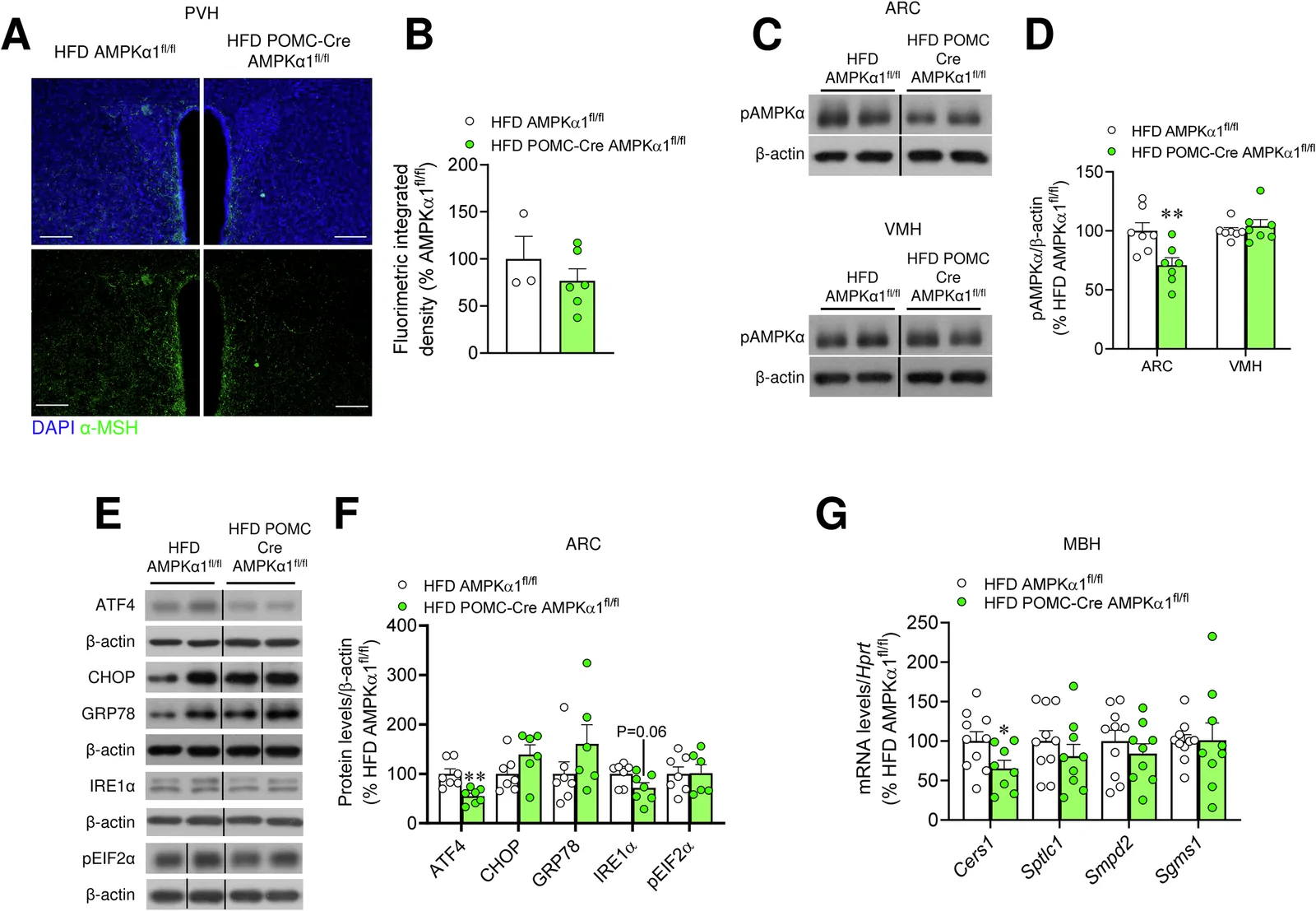
Effect of loss of AMPKα1 deletion in POMC neurons on the hypothalamus in HFD in male mice fed a HFD. (**A**) Representative confocal images of the paraventricular nucleus of the hypothalamus (PVH), depicting α-MSH immunoreactivity (green), (**B**) Quantification of the mean intensity of α-MSH (*n* = 3 control and 6 KO) of control (littermates AMPKα1^fl/fl^) and POMC-Cre AMPKα1^fl/fl^ male mice fed a HFD. Scale bar 150 μm. (**C**,** D**) Representative ARC and VMH pAMPKα western blot images (**C**) and pAMPKα protein levels in the ARC and VMH (**D**; *n* = 7 control and 7 KO; *P* = 0.009217) of HFD (littermates AMPKα1^fl/fl^) and POMC-Cre AMPKα1^fl/fl^ male mice fed a HFD. (**E**,** F**) Representative ARC ER stress pathway western blot images (**E**) and ER stress pathway protein levels in the ARC (**F**; *n* = 7 control and 6-7 KO; *P* = 0.002429 for ATF4; *P* = 0.061881 for IRE1α) of control (littermates AMPKα1^fl/fl^) and POMC-Cre AMPKα1^fl/fl^ male mice fed a HFD. (**G**) mRNA expression of ceramide pathway enzymes in the MBH (*n* = 10 control and 9-10 KO; *P* = 0.045272 for *Cers1*) of control (littermates AMPKα1^fl/fl^) and POMC-Cre AMPKα1^fl/fl^ male mice fed a HFD. In the Western blot analyses, β-actin was used as control. A black line between the immunoblots depicts samples loaded in the same gel, but not next to each other. Data were expressed as mean ± SEM. Statistical significance was assessed by Student’s *t*-test (**B**,** D**,** F**,** G**). *P < 0.05, **P < 0.01 vs. AMPKα1^fl/fl^. Source data are available online for this figure.

Our GO enrichment analysis highlighted several projection developmental processes. Given that our mouse line undergoes AMPKα1 deletion during embryogenesis, we decided to study POMC neuronal projections to the paraventricular nucleus of the hypothalamus (PVH) in male mice fed an HFD. We did not find differences in the PVH-αMSH fibers between the POMC-Cre AMPKα1^fl/fl^ mice fed a HFD and the littermate controls, likely excluding the possibility that our phenotype is driven by a developmental alteration of the ARC-PVH melanocortin system (Fig. 7A,B).

**Figure EV5:**
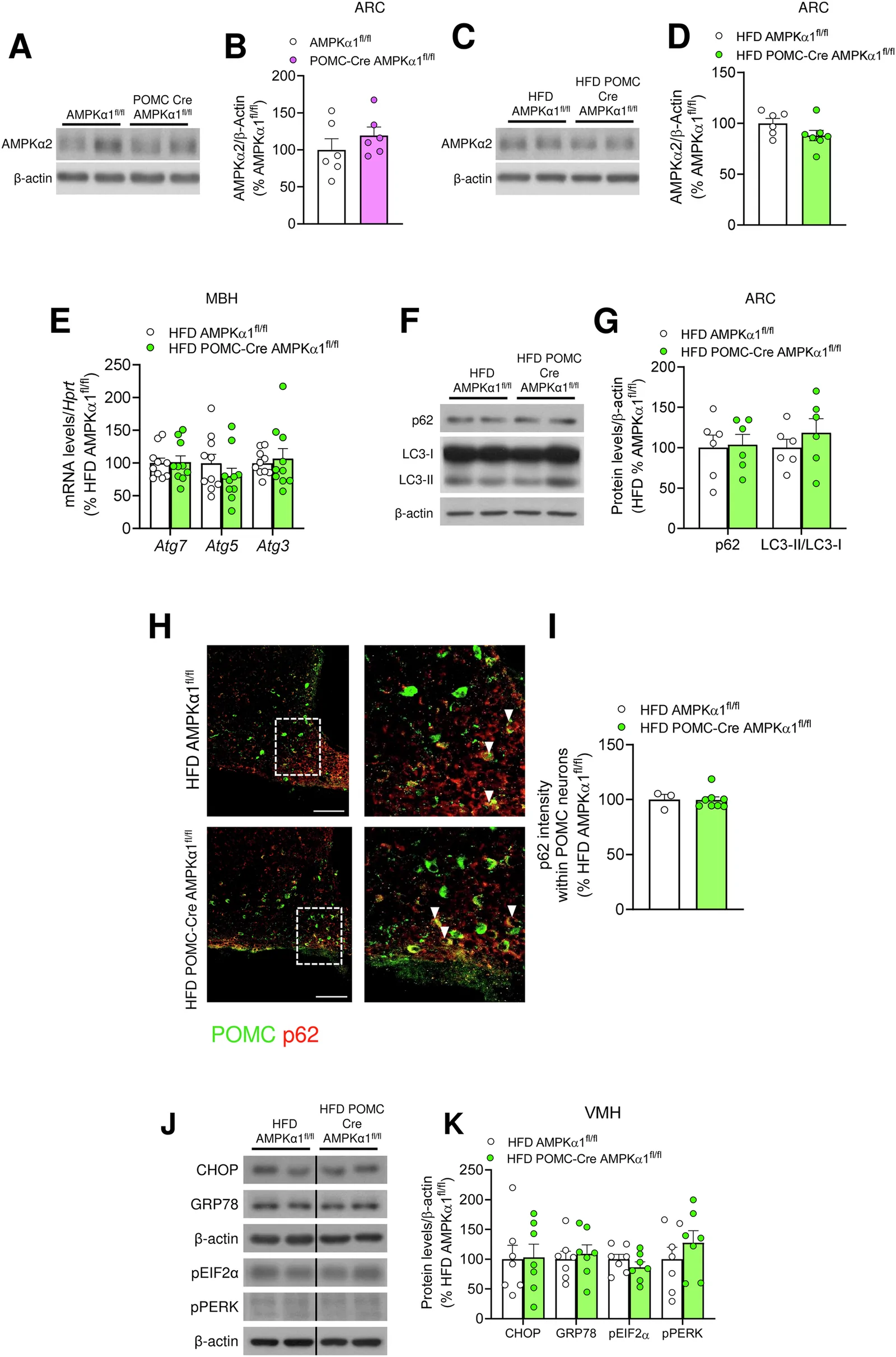
Effects of loss of AMPKα1 in POMC neurons on hypothalamic AMPKα2, autophagy and ER stress. (**A**, **B**) Representative ARC AMPKα2 western blot images (**A**) and AMPKα2 protein levels in the ARC (**B**; *n* = 6 control and 6 KO) of control (littermates AMPKα1^fl/fl^) and POMC-Cre AMPKα1^fl/fl^ male mice. (**C**,** D**) Representative ARC AMPKα2 western blot images (**C**) and AMPKα2 protein levels in the ARC (**D**; *n* = 6 control and 7 KO) of control (littermates AMPKα1^fl/fl^) and POMC-Cre AMPKα1^fl/fl^ male mice in HFD. (**E**) mRNA expression of autophagy-related proteins in the MBH (*n* = 10 control and 10 KO) of control (littermates AMPKα1^fl/fl^) and POMC-Cre AMPKα1^fl/fl^ male mice fed a HFD. (**F**,** G**) Representative ARC autophagy markers western blot images (**F**) and autophagy markers protein levels in the ARC (**G**; *n* = 6 control and 6 KO) of control (littermates AMPKα1^fl/fl^) and POMC-Cre AMPKα1^fl/fl^ male mice fed a HFD. (**H**,** I**) Representative confocal images from the ARC of HFD AMPKα1^fl/fl^ mice and HFD POMC-Cre AMPKα1^fl/fl^ mice showing p62 (red) and POMC (green) immunofluorescent staining (white arrowheads indicate cells coexpressing POMC and p62; scale bar 100 μm; zoomed images come from the squared region in the first image, depicting details of POMC neurons colocalization with p62) and quantification of the p62 intensity within POMC neurons (**I**; *n* = 3 control and 7 KO) of control (littermates AMPKα1^fl/fl^) and POMC-Cre AMPKα1^fl/fl^ male mice in HFD. (**J**,** K**) Representative VMH ER stress pathway western blot images (**J**) and ER stress pathway protein levels in the VMH (**K**; *n* = 7 control and 6-7 KO) of control (littermates AMPKα1^fl/fl^) and POMC-Cre AMPKα1^fl/fl^ male mice fed a HFD. In the western blot analyses, β-actin was used as control. A black line between the immunoblots depicts samples loaded in the same gel, but not next to each other. Data were expressed as mean ± SEM. Statistical significance was assessed by Student’s *t*-test (**B**,** D**,** E**,** G**,** I**,** K**).

Considering our previous findings, we decided to explore two possible AMPK-linked functions, namely ER stress and autophagy, that may contribute to the predicted alterations in exocytosis and vesicle transport. AMPK promotes autophagy (Li and Chen, 2019), a cell recycling process that has been described in ARC melanocortin neurons as a molecular mechanism controlling energy homeostasis (Coupe et al, 2012; Kaushik et al, 2012; Schneeberger et al, 2012). ATG7 (a key protein in the autophagy pathway) KO mice in AgRP neurons develop a decreased body weight and adipose mass (Kaushik et al, 2011). When autophagy related 7 (ATG7) was selectively ablated in POMC neurons, it leads to increased postweaning body weight, and higher adiposity (Coupe et al, 2012). We therefore hypothesized that autophagy may act as a downstream signal mediating the AMPKɑ1 role in obesity in POMC-Cre AMPKα1^fl/fl^ mice fed a HFD. To test this, we first confirmed that our model had a specific downregulation of the AMPK pathway (measured by a decreased pAMPK in the KO mice) in the ARC, but not in the VMH (Fig. 7C,D). No upregulation of AMPKα2 subunit was found in the ARC of POMC-Cre AMPKα1^fl/fl^ mice, either in chow diet (Fig. EV5A,B) or in HFD (Fig. EV5C,D) excluding the existence of any potential compensatory action between AMPKα subunits. Next, we investigated the hypothalamic mRNA expression of the main autophagy markers, namely *Atg7*, autophagy related 5 (*Atg5*), autophagy related 3 (*Atg3*), but no changes were detected (Fig. EV5E). Similarly, the protein levels of other autophagy markers, such as protein 62 (p62), and microtubule-associated protein light chain 3 (LC3-II/LC3-I) (Fig. EV5F,G) were also unchanged between null mice and the littermate controls fed a HFD. To achieve more cell-specificity in our analyses, we quantified p62 levels in POMC neurons from WT and POMC-Cre AMPKα1^fl/fl^ fed a HFD. Our data revealed no significant changes in p62 levels in POMC neurons of null mice (Fig. EV5H,I). Overall, this evidence did not support a primary role for autophagy in mediating the metabolic effects of POMC AMPKα1 deletion.

Hypothalamic ER stress also plays a prominent role in the regulation of energy balance (Contreras et al, 2014; Contreras et al, 2017; Dragano et al, 2024; Martinez-Sanchez et al, 2017b), being relevant in POMC neurons where it is associated with obese phenotypes (Dragano et al, 2024; He et al, 2021; Schneeberger et al, 2013; Williams et al, 2014; Xiao et al, 2017). To assess this potential mechanism in our model, we measured the protein levels of five key markers of the unfolded protein response (UPR): activating transcription factor 4 (ATF4), CHOP (C/EBP homologous protein), GRP78 (78 kDa glucose-regulated protein; a chaperone also known as BiP: binding immunoglobulin protein), and inositol-requiring enzyme 1α (IRE1α; an ER stress sensor), along with pEIF2α (phosphorylated eukaryotic initiation factor 2α). We observed a trend toward increased GRP78 expression, indicating enhanced capacity to handle unfolded proteins, accompanied by decreased IRE1α and a strong reduction in ATF4 in the ARC of POMC-Cre AMPKα1^fl/fl^ mice (Fig. 7E,F). These changes collectively reflect reduced ER stress. (Contreras et al, 2014; Contreras et al, 2017; Dragano et al, 2024; Martinez-Sanchez et al, 2017b). Of note, differences in ER stress were nucleus specific since the VMH had comparable levels of the main UPR proteins, including phosphorylated protein kinase-like endoplasmic reticulum kinase (pPERK), between floxed and of POMC-Cre AMPKα1^fl/fl^ mice (Fig. EV5J,K). Consequently, our results suggest that the blunted weight gain associated with AMPKα1 ablation in POMC neurons is in part mediated by the ameliorated ER stress, specifically within the ARC.

**Figure 8:**
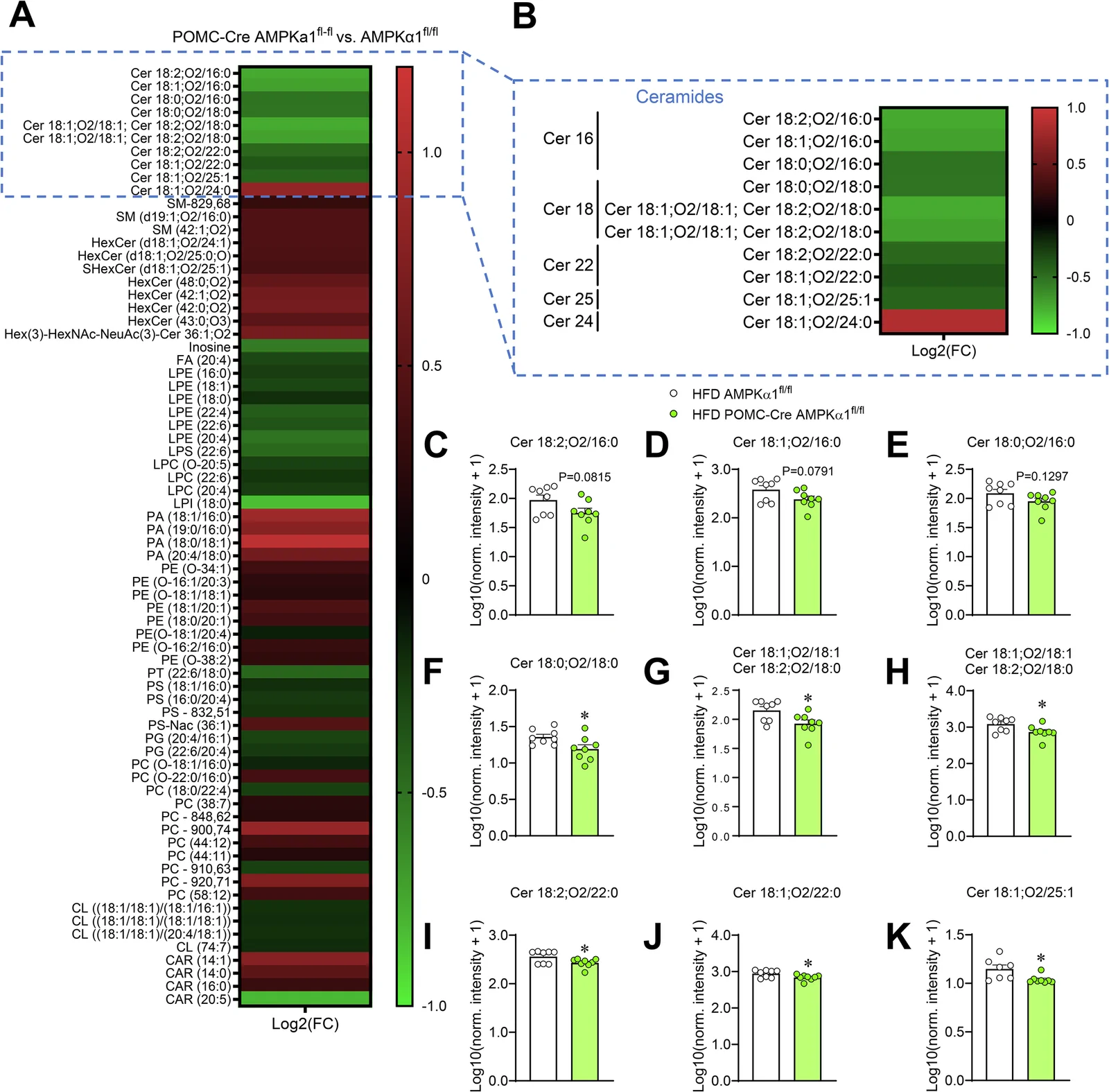
Effect of loss of AMPKα1 deletion in POMC neurons on the hypothalamic lipidomic profile in male mice fed a HFD. (**A**) Heat map depicting lipid species detected by liquid chromatography coupled to mass spectrometry (LC/MS) and differentially enriched (significantly) in the mediobasal hypothalamus (MBH; containing the ARC) of control (littermates AMPKα1^fl/fl^) and POMC-Cre AMPKα1^fl/fl^ male mice fed a HFD. (**B**) Detailed zoom in showing species of ceramides with the highest Log2 (FC) values. (**C**–**K**) Individual ceramide species: (**C**) Cer 18:2;O2/16:0 (*P* = 0.0815), (**D**) Cer 18:1;O2/16:0 (*P* = 0.0791), (**E**) Cer 18:0;O2/16:0 (*P* = 0.1289), (**F**) Cer 18:0;O2/18:0 (*P* = 0.0320), (**G**,** H**) Cer 18:1;O2/18:1 and Cer 18:2;O2/18:0 isomers (*P* = 0.0253 and *P* = 0.0349), (**I**) Cer 18:2;O2/22:0 (*P* = 0.0356), (**J**) Cer 18:1;O2/22:0 (*P* = 0.0308) and (**K**) Cer 18:1;O2/25:1 (*P* = 0.0195). Data were expressed as mean ± SEM. Statistical significance was assessed by Student’s *t*-test (**C**–**K**). *P < 0.05 vs. AMPKα1^fl/fl^. Source data are available online for this figure.

Ceramide-induced lipotoxicity is a canonical mechanism inducing hypothalamic ER stress (Contreras et al, 2014; Gonzalez-Garcia et al, 2018; Heras et al, 2020; Lopez et al, 2025; Martinez-Sanchez et al, 2017b; Meneyrol et al, 2021). Therefore, we explored the mRNA expression of the ceramide synthesis pathway by measuring the expression of ceramide synthase 1 (*Cers1*), serine palmitoyltransferase long chain base subunit 1 (*Sptlc1*), sphingomyelin phosphodiesterase 2 (*Smpd2*) and sphingomyelin synthase 1 (*Sgms1*) in the ARC of POMC-Cre AMPKα1^fl/fl^ mice. We found a decrease in *Cers1* mRNA expression, which could account for a decreased ceramide synthesis (Hammerschmidt et al, 2023), in the ARC of POMC-Cre AMPKα1^fl/fl^ mice fed a HFD (Fig. 7G). To address this, we performed targeted lipidomics on mediobasal hypothalamus (MBH, containing the ARC) tissue from POMC-Cre AMPKα1^fl/fl^ mice and control male mice fed a HFD, focusing on ceramide species. The analysis identified widespread alterations in lipid classes including sphingomyelins and glycerophospholipids (Fig. 8A), with prominent reductions in ceramides (Fig. 8A,B; Dataset EV1). Short-chain (C16) ceramides were decreased, including Cer 18:2;O2/16:0 (Fig. 8C), Cer 18:1;O2/16:0 (Fig. 8D), and Cer 18:0;O2/16:0 (Fig. 8E). Medium-chain (C18) species such as Cer 18:0;O2/18:0 (Fig. 8F) and Cer 18:1;O2/18:1/Cer 18:2;O2/18:0 isomers (Fig. 8G,H) were also reduced; a biochemical change that perfectly mirrored the downregulation in *Cers1* (Fig. 7G), the enzyme responsible for C18 ceramide synthesis. Very long-chain ceramides showed marked declines, notably Cer 18:2;O2/22:0 (Fig. 8I), Cer 18:1;O2/22:0 (Fig. 8J), and Cer 18:1;O2/25:1 (Fig. 8K). These data indicate a global suppression of ceramide levels in the MBH upon AMPKα1 deletion in POMC neurons. These detailed lipidomics data unequivocally validate the functional consequences of the phosphoproteomic changes, demonstrating that POMC AMPKα1 deletion leads to reduced hypothalamic ceramide burden under obesogenic conditions. This would be consistent with the increased BAT thermogenesis in these animals (Contreras et al, 2014; Gonzalez-Garcia et al, 2018; Martinez-Sanchez et al, 2017b). Overall, these results suggested that selective ablation of AMPKα1 in POMC neurons would lead to reduced ceramide-induced lipotoxicity in the ARC and, subsequently, to decreased hypothalamic ER stress, promoting increased BAT thermogenesis and weight loss under HFD.

## Discussion

In recent years it has become clear that the different AMPK subunits display a complex functional and nuclei-dependent role controlling energy balance within the hypothalamus (Freire-Agulleiro et al, 2025; Martinez-Sanchez et al, 2017b; Milbank et al, 2023; Milbank et al, 2021; Okamoto et al, 2018; Seoane-Collazo et al, 2018). In the ARC, AMPKα2 deletion in AgRP neurons induced an age-dependent lean phenotype, while its deletion in POMC neurons promotes an increase in body weight both in chow and HFD due to reduced energy expenditure and exacerbated refeeding response after fasting (Claret et al, 2007). Interestingly, these AMPKα loss-of-function models previously described contrast with the one reported herein, where AMPKα1 ablation in the same POMC neuronal population, specifically in males, drives an increased thermogenesis, coupled to a protection against diet-induced obesity. Several aspects might explain the differences found. (i) Firstly, the subcellular location of AMPKα1 and AMPKα2. AMPKα2, but not AMPKα1, has a nuclear localization signal (NLS) which is key for its nuclear translocation (Afinanisa et al, 2021; Suzuki et al, 2007). This suggests that AMPKα1-containing heterotrimers mainly regulate cytosolic substrates, while those AMPKα2-based would control transcriptional machinery that regulates gene expression (Afinanisa et al, 2021; Suzuki et al, 2007). This might also explain why in our model we observe changes in ER stress, while the AMPKα2 POMC KO mice reported by Claret and colleagues exhibited altered neuropeptide expression (Claret et al, 2007). (ii) Secondly, POMC neuronal heterogeneity should also be considered. This neuronal population is composed of different subclusters, which respond to diverse combinations of endocrine and metabolic signals (Biglari et al, 2021; Campbell et al, 2017; Chen et al, 2017; Lam et al, 2017; Leon et al, 2024; Quarta et al, 2021; Quarta et al, 2020; Saucisse et al, 2021). Given that AMPKα1 and AMPKα2 display a heterogeneous expression in POMC neurons (Steuernagel et al, 2022), they might integrate different cues and exert different mechanisms through specific neuronal projections. (iii) Thirdly, the *Pomc*-Cre lines used to generate the AMPKα2 and AMPKα1 knockout mice were different (Claret et al, 2007). This might have resulted in the genetic target of different POMC subpopulations (Leon et al, 2024; Quarta et al, 2021; Quarta et al, 2020; Saucisse et al, 2021), triggering the mentioned differences in signal integration and downstream projections, not only during postnatal life but also during embryogenesis. Given its role as gene expression regulator, AMPKα2 deletion might induce differences in POMC neurons development, something that seems unlikely in our model, since no differences were detected in GABAergic and glutamatergic POMC neurons subpopulations in our analyses.

The constitutive POMC-Cre driver may introduce developmental compensation or circuit alterations, a known limitation of non-inducible systems. Inducible POMC-Cre models will enable adult-onset deletion, enabling clearer attribution to mature AMPKα1 and AMPKα2 signaling. Regardless of these considerations, the overall conclusion from the comparison between our data with the former literature indicates that the role of hypothalamic AMPK is not only nucleus-dependent but rather subunit-dependent (Seoane-Collazo et al, 2018). This finding reinforces the idea that within the hypothalamus AMPKα1 and AMPKα2 are indeed different protein kinases (Seoane-Collazo et al, 2018), are regulated in different ways, and engage divergent downstream signaling pathways. This is further supported by the fact that none of those changes seem to be mediated by a compensatory action of the non-ablated subunit (Claret et al, 2007; Seoane-Collazo et al, 2018).

Notably, our comprehensive phenotyping revealed a striking sexual dimorphism in POMC neuronal AMPKα1 function: whereas males exhibit robust HFD resistance through elevated basal BAT thermogenesis and SNS outflow, females display no significant metabolic alterations even under obesogenic challenge. This sex-dependent phenotype likely reflects gonadal hormone modulation of hypothalamic nutrient sensing, with estrogen potentially affecting AMPKα1-dependent signaling or SNS output in POMC circuits, which is consistent with the known sexual dimorphisms in ARC NPY/AgRP-POMC interactions (Gonzalez-Garcia et al, 2023; López and Tena-Sempere, 2017; Martínez de Morentin et al, 2014; Martinez de Morentin et al, 2015).

Our phosphoproteomics identified enrichment in exocytosis and vesicle transport in the POMC-Cre AMPKα1^flox/flox^ mice when compared to the littermate controls in HFD. Together with this, the same animal model and experimental paradigm [HFD is a classical inductor of ER stress in the hypothalamus (Contreras et al, 2014; Contreras et al, 2017; Dragano et al, 2024; Schneeberger et al, 2013)] displayed a downregulated ER stress in the ARC. In line with this, we have previously demonstrated that AMPK inhibition decreases ER stress by reducing ceramide-induced lipotoxicity in the VMH (Contreras et al, 2014; Gonzalez-Garcia et al, 2018; Lopez et al, 2025; Martinez-Sanchez et al, 2017b). Therefore, it was plausible to conclude that the same molecular mechanism might operate in neighboring POMC neurons within the ARC. Our expression and lipidomic data confirmed decreased ceramide synthesis in the ARC of POMC-Cre AMPKα1^flox/flox^ mice, demonstrating that the regulation of complex lipid metabolism by AMPKα1 represents a canonical mechanism shared by both SF1 (Martinez-Sanchez et al, 2017b) and POMC neurons. Interestingly, high ER stress levels in the hypothalamus and more specifically in POMC neurons have been associated with obese phenotypes (Dragano et al, 2024; He et al, 2021; Schneeberger et al, 2013; Williams et al, 2014; Xiao et al, 2017) and their amelioration drives an increase in BAT thermogenesis, which improves the energy imbalance. Notably, one of the downregulated ER stress markers we found, ATF4, was previously studied in hypothalamic POMC neurons and its specific ablation in this population reproduces a similar phenotype to our mice, where body weight is reduced because of increased energy expenditure (Xiao et al, 2017).

All this evidence might suggest that changes in ER stress, exocytosis and vesicle-mediated transport could induce an enhancement in the peptidergic output of POMC neurons facilitating the release of α-MSH in pre-autonomic sympathetic areas, other than the PVH, projecting to BAT. In this sense, it is known that POMC neurons modulate BAT thermogenesis through their control of the SNS (Bell et al, 2018; Jiang et al, 2020; Quarta et al, 2021). Several studies have demonstrated how these neurons project to pre-autonomic regions in the brainstem, where they modulate SNS tone to BAT (Bell et al, 2018; Joly-Amado et al, 2014; Quarta et al, 2021). The POMC-BAT regulation seems particularly relevant in the obesity context, as several groups have found that under diet-induced obesity paradigms, genetic loss-of-function models trigger an alteration in BAT thermogenesis, which leads to weight gain (Dodd et al, 2015; Qiu et al, 2024; Schneeberger et al, 2013; Tang et al, 2024). It is interesting to highlight how ER stress induced by HFD acts as a causal event leading to a downregulation in BAT thermogenesis and energy expenditure, which contributes to the increased body weight (Contreras et al, 2017; Schneeberger et al, 2013). Our POMC-Cre AMPKα1^fl/fl^ mice fit in this pathophysiological mechanism, since we found a decreased ATF4 and IREα1 expression in the ARC together with an increase in GRP78, all proteins components of the UPR, that indicate a decrease in two of the molecular branches of ER stress signaling and a higher capability to buffer the unfolded proteins in the ER, respectively (Martinez de Morentin and Lopez, 2010). Accordingly, the POMC-Cre AMPKα1^flox/flox^ mice exhibit increased BAT thermogenesis compared to their littermate controls, but only in the HFD context. This confirms that POMC neurons are key elements in the energy imbalance and hypothalamic ER stress induction that characterizes obesity.

In summary, our study reveals that AMPKα1 ablation in POMC neurons has a protective effect over diet-induced obesity, specifically in males. The reduced body weight observed in null male mice is associated with increased BAT thermogenesis and decreased ceramide-induced hypothalamic ER stress. These findings highlight **i**) the complex nature of the different AMPK subunits in the hypothalamic regulation of energy balance and **ii**) emphasize the existence of sex-dependent differences in hypothalamic energy sensing. Overall, these findings reinforce the notion that AMPKα1 in POMC neurons represents both a key component of the pathophysiological mechanism driving to obesity and a promising target for therapeutic interventions, and suggests that AMPKα1-targeted therapies for obesity (Milbank et al, 2023; Milbank et al, 2021; Mukherjee et al, 2023) may require sex-specific optimization to achieve efficacy across diverse patient populations.

## Methods

**Table:** Reagents and tools table

Reagent/resource	Reference or source	Identifier or catalog number
**Experimental models**		
POMC-Cre mice	The Jackson Laboratory	B6.FVB-Tg(Pomc-cre)1Lowl/J - Cat. 010714
AMPKα1 floxed mice	The Jackson Laboratory	Prkaa1^tm1.1Sjm/J^ – Cat. 014141
Ai14-tdTomato mice	The Jackson Laboratory	B6.Cg-Gt(ROSA)26Sortm^14(CAG-tdTomato)Hze/J^ – Cat. 007914
**Antibodies**		
α-tubulin	Sigma-Aldrich	T5168
β-actin	Sigma-Aldrich	A5316
ATF4	Sigma-Aldrich	WH0000468M1
CHOP	Santa Cruz	sc-793
GRP78	Cell Signaling	3183S
IRE1α	Cell Signaling	3294S
LC3-I/II	Cell Signaling	4180S
p62	Abcam	ab56416
pACCα (Ser79)	Cell Signalling	3661S
pAMPKα (Trh172)	Cell Signalling	2535S
pEIF2α (Ser52)	Santa Cruz	sc-101670
pPERK (Thr980)	Cell Signalling	3179S
UCP1	Abcam	ab10983
Polyclonal Rabbit anti-mouse immunoglobulinHPR	Dako	P0260
Polyclonal Rabbit anti-rabbit immunoglobulinHPR	Dako	P0448
**PCR genotyping primers**		
AMPKα1 floxed alleles	Forward 5’- CCC ACC ATC ACT CCA TCT CT - 3’ Reverse 5’ - AGC CTG CTT GGC ACA CTT AT – 3’	
AMPKα1 erased allele	Forward 5’- CCC ACC ATC ACT CCA TCT CT - 3’ Reverse 5’- CCCACATAGGAAAGCGTGTT -3’	
**qPCR primers and probes**		
*Cers1*	Applied Biosystems TaqMan	Mm03024093_mH
*Sptlc1*	Applied Biosystems TaqMan	Mm00447343_m1
*Smpd2*	Applied Biosystems TaqMan	Mm01188195_g1
*Smgs1*	Applied Biosystems TaqMan	Mm00522643_m1
*Atg7*	Forward primer 5’-AGCGGCGGCTGGTAAGAA-3’; Reverse primer 5’-TCTTCTGGGTCAGTTCGTGC-3’	
*Atg5*	Forward primer 5’-GCTTTTGCCAAGAGTCAGCTA-3’; Reverse primer 5’- TCTGGAAAACTCATTTCAGGGGT-3’	
*Atg3*	Forward primer 5’-GCAGAGTCAGGGTGCTATTGA-3’; Reverse primer 5’- GCACAGAGCAGATGTAGGGA -3’	
*Hprt*	Forward Primer 5′-AGCCGACCGGTTCTGTCAT-3’; Reverse Primer 5′-GGTCATAACCTGGTTCATCATCAC-3’; Probe FAM-5′-CGACCCTCAGTCCCAGCGTCGTGAT-3′-TAMRA	
**Probes for** **in situ** **hybridization and RNAScope**		
*PrKaa1-*ex3 riboprobe	Forward T3-AMPKex3-sense (5’-CAGAGATGCAATTAACCCTCACTAAAGGGAGAGTACCAGGTCATCAGTACACCATCT-3’ Reverse T7-AMPKex3-as (5’-CCAAGCCTTCTAATACGACTCACTATAGGGAGACCTTCCATTTTTACAGATATAATCA -3’)	
*Pomc* riboprobe	Forward - 3-POMC-sense (5’-CAGAGATGCAATTAACCCTCACTAAAGGGAGACCTCCTGCTTCAGACCTCCAT-3’ Reverse T7-POMC-as (5’-CCAAGCCTTCTAATACGACTCACTATAGGGAGATGCTCCATGGAGTAGGAGCGC-3’)	
*Pomc* probe (RNA Scope)	Advanced Cell Diagnostics	314081-C3
*Gad1* probe (RNA Scope)	Advanced Cell Diagnostics	400951-C1
*Gad2* probe (RNA Scope)	Advanced Cell Diagnostics	439371-C1
*Slc17a6* probe (RNA Scope)	Advanced Cell Diagnostics	456751-C2
**Chemicals, diets and other reagents**		
High-fat diet	Research Diets	D12451
Chow diet	Global Diet	2918
D-Glucose	Sigma-Aldrich	G8270
Insulin	Novo Nordisk	Actrapid 100 UI/ml,
10% Neutral Buffered Formalin	Sigma-Aldrich	HT501128
RNAscope Fluorescent Multiplex Detection Reagent kit	Advanced Cell Diagnostics	323110
Opal650	Akoya Biosciences	FP1496001KT
Opal520	Akoya Biosciences	FP1487001KT
Opal570	Akoya Biosciences	FP1488001KT
ProLong Gold Antifade Mountant	Thermo Fisher Scientific	P36930
TRIzol	Invitrogen	15596026
SuperScript First-Strand Synthesis System	Invitrogen	18091050
Protease inhibitor	Roche	Complete Mini EDTA-free and phosSTOP
Protein detection kit	Bio-Rad Laboratories	RC DC Protein Assay Kit
**Software**		
FLIR Tools	FLIR	FLIR Tools - Version 5.13.18031.2002
Leica Application Suite	Leica	v3.5.7
FIJI	NIH	
Shimadzu LC solution	Shimadzu	
Proteome Discoverer 2.5	Thermo Scientific	
RStudio		v1.2.1335
MassFrontiers	Thermo Scientific	version 8.0 SR1
Graphpad Prism	Graphpad	8.0.2
**Other**		
Infrared thermal camera	FLIR	B335
Glucometer	Roche	Accu-Check Guide
Calorimetric system	TSE Systems	LabMaster
Nuclear magnetic resonance	EchoMRI	EchoMRI-100H
Cryostat	Leica	NX50
Superfrost Plus Gold slides	Thermo Fisher Scientific	J1800AMNZ
HybEZ oven	Advanced Cell Diagnostics	HybEZ™ II Hybridization System
HybEZ Humidity Control Tray	Advanced Cell Diagnostics	HybEZ Humidity Control Tray
Confocal microscope	Leica	Leica DM6 CFS TCS SP8
Thermocycler	Applied Biopsystems	QuantStudio 5 Real-Time PCR
High-performance liquid chromatography (HPLC)	Shimadzu Corporation	Shimadzu LC Prominence
Analytical columns	Waters Symmetry	300C18
Coulometric electrochemical detector	Thermo Scientific	ESA Coulochem III
Liquid chromatoghrap	Thermo Scientific	Thermo Scientific EASY-nLC 1000
Mass spectrometer	Thermo Scientific	Tribrid Orbitrap
Column	Thermo Scientific	PepMap100 C18 LC 75 µm ID, 2 cm
Liquid chromatography coupled to mass spectrometry	Thermo Scientific Waltham	Vanquish Horizon UHPLC Orbitrap ID-X Tribrid Mass Spectrometer

### Animals

To generate ARC-specific POMC neuron AMPKα1 knock-out mice, POMC-Cre mice (B6.FVB-Tg(Pomc-cre)1Lowl/J, catalog number 010714; The Jackson Laboratory) (Dragano et al, 2024; Quinones et al, 2018) were crossed with AMPKα1 floxed mice (Prkaa1tm1.1Sjm/J, catalog number 014141; The Jackson Laboratory) (Martinez-Sanchez et al, 2017b; Seoane-Collazo et al, 2018). Mice were bred to generate both POMC-Cre AMPKα1^fl/fl^ (KO mice) and AMPKα1^fl/fl^ (control littermates). POMC-Cre AMPKα1^fl/fl^ were also bred with Ai14-tdTomato animals (B6.Cg-Gt(ROSA)26Sortm14(CAG-tdTomato)Hze/J, catalog number 007914; The Jackson Laboratory) to obtain POMC-Cre Ai14-tdTomato AMPKα1^fl/fl^, for histological characterization and validation of Cre-mediated recombination. Animal genotyping was performed according to the primers and PCR protocols provided by The Jackson Laboratory.

Male and female mice were housed (Centro de Biomedicina Experimental, CEBEGA University of Santiago de Compostela, USC) under a 12-h artificial light cycle (08:00 to 20:00), maintaining a controlled temperature (22–24 °C; with the exception of the cold exposure, in which animals were exposed to 4 °C for 6 h) and humidity (55%), and provided with free access (ad libitum) to either standard laboratory chow (2918 Global Diet) or high-fat diet (45 kcal% from fat; D12451, Research Diets) and tap water. All mice were group-housed, except for periods designated for food intake analyses, temperature measurements, infrared thermography measurements for BAT temperature, and indirect calorimetry. Food intake was measured for 7 consecutive days, and the average daily food intake for the whole period was plotted. The experiments were conducted in accordance with the International Law on Animal Experimentation and were approved by USC Ethical Committee (Project ID 15012/2020/010). No blinding was performed for the conduct of experiments. The study was designed, conducted, and reported in accordance with the ARRIVE guidelines to ensure transparent and reproducible reporting of animal research.

### Temperature measurements

Core body temperature was measured daily for 7 days with a rectal probe connected to a digital thermometer (BAT-12: Microprobe-Thermometer) (Freire-Agulleiro et al, 2025; Lopez-Rey et al, 2026; Seoane-Collazo et al, 2018). Skin temperature surrounding the interscapular BAT was recorded with a B335 compact infrared thermal imaging camera (FLIR) and analyzed with FLIR Tools software (FLIR Systems), as previously described (Dragano et al, 2024; Freire-Agulleiro et al, 2025; Gonzalez-Garcia et al, 2022; Milbank et al, 2023; Milbank et al, 2021).

### Glucose and insulin tolerance test

Basal blood glucose levels were measured after an overnight fast (12 h during the dark phase) for the glucose tolerance test (GTT), and after 6 h (during the light phase) for the insulin tolerance test (ITT), with a glucometer (Accu-Chek, Roche). The GTT and ITT were performed after an intraperitoneal injection of 2 g/kg D-glucose (Sigma-Aldrich) or 0.7 (STD mice) and 1 (HFD mice) U/kg insulin (Novo Nordisk), respectively. Blood glucose measured at baseline (0 min) and post-injection time points 15, 30, 60, 90, and 120 min for GTT and 10/15, 20, 30, 60, and 90 min for ITT, Glucose levels and area under the curve (AUC) values were determined as previously described (Freire-Agulleiro et al, 2025; Gonzalez-Garcia et al, 2022; Seoane-Collazo et al, 2018). Fed glucose levels were assessed during the light phase and at the end of food intake measurements over 7 days.

### Indirect calorimetry and nuclear magnetic resonance

Animals were analyzed for VO_2_ EE, RER, and LA using calorimetric systems (LabMaster; TSE Systems) as previously described (Dragano et al, 2024; Freire-Agulleiro et al, 2025; Gonzalez-Garcia et al, 2022; Milbank et al, 2021). Continuous 48-h or 120-h monitoring of VO_2_, EE, RER, and LA were recorded every 30 min across light/dark cycles. For body composition analysis, nuclear magnetic resonance (NMR; EchoMRI) was employed (Dragano et al, 2024; Gonzalez-Garcia et al, 2018; Milbank et al, 2021; Seoane-Collazo et al, 2019).

### Sample processing

Mice were killed by cervical dislocation and decapitation. From each mouse, the mediobasal hypothalamus (MBH), the VMH and ARC [dissected from the hypothalamus, as shown (Gonzalez-Garcia et al, 2018; Milbank et al, 2021; Seoane-Collazo et al, 2018)], as well as the cerebellum, nucleus tractus solitarius (NTS), cortex, liver, BAT, sWAT and gonadal WAT (gWAT) were immediately homogenized on ice. Liver, BAT, sWAT, and gWAT were weighed. Samples and serum were stored at −80 °C until further processing.

### PCR for recombined allele detection

Cre-mediated recombination was assessed by PCR in both *Pomc* expressing (ARC and NTS) and non-expressing tissues (cerebellum, cortex, WAT and liver), for both control littermates and POMC-Cre AMPKα1^fl/fl^. A specific antisense primer 5’- CCCACATAGGAAAGCGTGTT -3’ was employed in combination with the forward primer used for genotyping to detect the recombined *Prkaa1* allele (531 bp) over the floxed one, as shown (Martinez-Sanchez et al, 2017b; Seoane-Collazo et al, 2018).

### Double in situ hybridization

In situ hybridization (ISH) analyses were performed as previously shown (Manfredi-Lozano et al, 2016) using specific antisense riboprobes complementary to the coding sequence of the whole exon 3 of mice *Prkaa1 mRNA* (*PrKaa1-*ex3 riboprobe; from 278- to 371-nt; NM_001013367.3) and *Pomc mRNA* (*Pomc* riboprobe; from 376- to 717-nt; NM_001278581). For the generation of the template, primer sequences were as follows: *PrKaa1-*ex3 riboprobe [forward T3-AMPKex3-sense (5’-CAGAGATGCAATTAACCCTCACTAAAGGGAGAGTACCAGGTCATCAGTACACCATCT-3’); reverse T7-AMPKex3-as (5’-CCAAGCCTTCTAATACGACTCACTATAGGGAGACCTTCCATTTTTACAGATATAATCA -3’)]; *Pomc* riboprobe [forward T3-POMC-sense (5’-CAGAGATGCAATTAACCCTCACTAAAGGGAGACCTCCTGCTTCAGACCTCCAT-3’); reverse T7-POMC-as (5’-CCAAGCCTTCTAATACGACTCACTATAGGGAGATGCTCCATGGAGTAGGAGCGC-3’)]. For double ISH *PrKaa1-*ex3 riboprobe was radiolabelled with [^33^P]-UTP (Perkin Elmer), while *Pomc* riboprobe was labeled with UTP-digoxigenin using DIG labeling mix (Roche). Following hybridization, sections were processed for the detection of Pomc DIG-labeled riboprobe using an anti-DIG antibody conjugated to alkaline phosphatase (Roche; dilution 1:200; incubation overnight at room temperature). Next, staining detection was developed by incubating with ImmPACT Vector Red alkaline phosphatase substrate following the manufacturer’s instructions (Vector Laboratories). After, the slices were dried, dipped in Kodak Autoradiography Emulsion type NTB (Kodak) and exposed for 3 weeks at 4 °C in dark for *PrKaa1-*ex3-radiolabelled riboprobe detection. After this period, the sections were developed and fixed. For evaluation of colocalization, DIG-labeled cells (POMC neurons) were located under the microscope and considered positive for *PrKaa1-*ex3 expression when the number of silver grains deposited on POMC neurons exceeded that of the background by threefold (Kauffman et al, 2007). Data were expressed as the percentage of POMC neurons expressing *PrKaa1-*ex3 in each brain slice.

### Immunohistochemistry

Adult male POMC-Cre Ai14-tdTomato AMPKα1^fl/fl^, POMC-Cre AMPKα1^fl/fl^, and AMPKα1^fl/fl^ mice were transcardiacally perfused with a saline solution followed by 4% neutral buffered paraformaldehyde (PFA). Brains were dissected and post-fixed for 24 h with 4% PFA, followed by immersion in a 30% sucrose solution for dehydration. When brains sank, they were sectioned at 30 μm on a cryostat (Leica Biosystems). Sections were washed in 0.1 M TBS and blocked with 0.1 M TBS containing gelatin (0.25%) and Triton X-100 (0.5%) for 2 h at room temperature followed by incubation with primary antibody (Sheep anti-α-MSH; 1:10000; #AB5087 RRID: AB_91683; Merck) overnight at 4 °C. Sections were washed with 01 M PBS and incubated with secondary antibody (Alexa Fluor 555 donkey anti-sheep 1:1000; #A-21436; RRID: AB_2535857; Invitrogen) for 2 h at room temperature with 0.1 M TBS containing gelatin (0.25%) and Triton X-100 (0.5%) and further incubated with DAPI for nuclear staining. Images were obtained using a Leica AOBS-SP5X Confocal Microscope (Leica Microsystems), and analysis was performed using Fiji 2.0.0 (ImageJ; NIH). For α-MSH stainings maximum projections were further skeletonized and integrated density was calculated. For the p62/POMC staining, brain sections were washed in 0.1 M TBS and blocked with 0.1 M TBS containing donkey serum (5%) and Triton X-100 (0.25%) for 2 h at room temperature followed by incubation with primary antibody (Rabbit Anti-p62 antibody; 1:250; RRID: AB_2810880, #AB109012; Abcam) overnight at 4 °C. Sections were washed with 0.1 M TBS and incubated with secondary antibody (Donkey anti-Rabbit Alexa Fluor 647; RRID: AB_2536183; #A31573; Invitrogen) for 2 h at room temperature with 0.1 M TBS containing donkey serum (5%) and Triton X-100 (0.25%). Next, sections were washed with 0.1 M TBS and further incubated with Rabbit Anti-POMC antibody (1:1000; RRID: AB_2753188; #H-029-030; Phoenix Pharmaceutical) overnight at 4 °C with 0.1 M TBS containing donkey serum (5%) and Triton X-100 (0.25%). Finally, sections were washed with 0.1 M TBS and incubated with secondary antibody (Donkey anti-Rabbit Alexa Fluor 488; RRID: AB_2535792; #A21206; Invitrogen) for 2 h at room temperature with 0.1 M TBS containing donkey serum (5%) and Triton X-100 (0.25%) and further incubated with DAPI for nuclear staining. Images were obtained using a Leica AOBS-SP5X Confocal Microscope (Leica Microsystems), and analysis was performed using Fiji 2.0.0 (ImageJ; NIH). For p62/POMC staining, max projections were used to semi-automatically define POMC neurons and p62 intensity was calculated within the area of the selected POMC neurons.

sWAT samples were processed as shown (Contreras et al, 2017; Gonzalez-Garcia et al, 2022; Martinez-Sanchez et al, 2017a; Seoane-Collazo et al, 2019). For the adipocyte area, images were analyzed with the Adiposoft plugin in Fiji Software (NIH). sWAT sections were used for immunohistochemistry detection of uncoupling protein 1 (UCP1). UCP1-stained area quantified with Fiji image analysis software, as described elsewhere (Contreras et al, 2017; Gonzalez-Garcia et al, 2022; Martinez-Sanchez et al, 2017a; Seoane-Collazo et al, 2019). Three pictures per image section were analyzed for histological analysis.

### RNAscope

Adult male POMC-Cre AMPKα1^fl/fl^ and AMPKα1^fl/fl^ mice were transcardiacally perfused with ice-cold PBS, pH 7.4, followed by 4% paraformaldehyde (Sigma-Aldrich). Brains were extracted and post-fixed in 4% PFA overnight at 4 °C, then cryoprotected in 30% sucrose in PBS at 4 °C. Coronal sections (30 μm) were cut using a cryostat (CM1950; Leica). All reagents were purchased from Advanced Cell Diagnostics (ACD), except for the Opal fluorophores, which were obtained from Akoya Biosciences. The *Pomc* probe (314081-C3) contained ten oligonucleotide pairs targeting region 19-995 (NM_008895.3) of the *Pomc* transcript; the *Gad1* probe (400951-C1) contained 15 oligonucleotide pairs targeting region 62-3113 (NM_008077.4) of the *Gad1* transcript; the *Gad2* probe (439371-C1) contained 20 oligonucleotide pairs targeting region 522-1506 (NM_008078.2) of the *Gad2* transcript; the *Slc17a6* probe (456751-C2) contained 19 oligonucleotide pairs targeting region 3-1240 (NM_080853.3) of the *Slc17a6* transcript. Two brain sections per mouse were selected, based on the Allen Brain Atlas. Sections were mounted on SuperFrost Plus Gold slides (J1800AMNZ; Thermo Fisher Scientific), dried at room temperature for 1 h, baked at 60 °C for 1 h, briefly rehydrated in ultrapure (Milli-Q^®^ water), air-dried overnight, and baked again at 60 °C for 1 h the following day. For all baking steps, the slides were placed in a dry HybEZ Humidity Control Tray (without lid or humidifying paper), and incubated in the HybEZ oven at 60 °C. Subsequent steps—including tissue pretreatment, probe hybridization, and signal detection and amplification were carried out according to the manufacturer’s protocol for fixed-frozen samples (RNAscope Fluorescent Multiplex Detection Reagent kit; 323110). During pretreatment, slides were incubated in target retrieval solution for 5 min and in protease III for 30 min. Probes against *Pomc*(-C3) and *Slc17a6*(-C2) were each diluted 1:50 into a 1:1 mix of *Gad1*(-C1) and *Gad2*(-C1) probes, which were combined to label GABAergic neurons. Probes were then detected using tyramide signal amplification with the following fluorophores, each diluted 1:750: Opal650 (FP1496001KT; for *Gad1/Gad2*), Opal570 (FP1488001KT; for *Slc17a6*), and Opal520 (FP1487001KT; for *Pomc*). Sections were counterstained with DAPI and mounted with ProLong Gold Antifade Mountant (P36930; Thermo Fisher Scientific), with glass coverslips (thickness 0.13–0.17 mm). Slides were stored in the dark until imaging. Images were acquired using Leica Application Suite (v3.5.7) and a Leica DM6 CFS TCS SP8 confocal microscope equipped with an X20/1 dry objective available at the Bordeaux Imaging Centre (BIC). Optical sectioning was set at 0.9 µm to perform Z-stacks. A total of 4 hemi-ARC were imaged per mouse (two sections). Images were imported into FIJI (NIH) for analysis. For each image maximum intensities were projected and POMC positive cells were segregated using visual threshold. Then the plugin “Analyze Particle” was used to define single-cell ROIs. Mean fluorescence intensities for every channel were measured in each cell and positivity for each marker was assessed by individual thresholds.

### RT-qPCR

RNA was extracted using TRIzol reagent (Invitrogen) according to the manufacturer’s instructions. About 500 ng of total RNA were used for each RT reaction, and cDNA synthesis was performed using the SuperScript First-Strand Synthesis System (Invitrogen) and random primers. Negative control reactions, containing all reagents except the sample were used to ensure specificity of the PCR amplification. For Real-time PCR, we used a fluorescent temperature cycler (Applied Biosystems) following the manufacturer’s instructions and TaqMan (Applied Biosystems) commercially available validated probes for ceramide synthase 1 (*Cers1*; assay ID: Mm03024093_mH), serine palmitoyltransferase long chain base subunit1 (*Sptlc1*; assay ID: Mm00447343_m1), sphingomyelin phosphodiesterase 2 (*Smpd2*; assay ID: Mm01188195_g1), sphingomyelin synthase 1 (*Smgs1*; assay ID: Mm00522643_m1). For autophagy related 7 (*Atg7*; Forward primer 5’-AGCGGCGGCTGGTAAGAA-3’; Reverse primer 5’-TCTTCTGGGTCAGTTCGTGC-3’), autophagy related 5 (*Atg5*, forward primer 5’-GCTTTTGCCAAGAGTCAGCTA-3’; reverse primer 5’- TCTGGAAAACTCATTTCAGGGGT-3’), and autophagy related 3 (*Atg3*, forward primer 5’-GCAGAGTCAGGGTGCTATTGA-3’; reverse primer 5’- GCACAGAGCAGATGTAGGGA -3’), we employed SYBR green. Cycling conditions included an initial denaturation at 95 °C for 3 min followed by 40 cycles at 95 °C for 5 s and 60 °C for 32 s, with a holding stage of 95 °C for 15 s, 60 °C for 1 min, and 95 °C for 15 s. Primers values were expressed relative to hypoxanthine-guanine phosphoribosyl-transferase levels (*Hprt*; Forward Primer 5′-AGCCGACCGGTTCTGTCAT-3’; Reverse Primer 5′-GGTCATAACCTGGTTCATCATCAC-3’; Probe FAM-5′-CGACCCTCAGTCCCAGCGTCGTGAT-3′-TAMRA). All reactions were performed in duplicate using the QuantStudio 5 Real-Time PCR (qPCR) as shown (Freire-Agulleiro et al, 2025; Gonzalez-Garcia et al, 2022; Milbank et al, 2021).

### Western blot

Protein lysates from VMH, ARC and BAT were separated by SDS-PAGE, electrotransferred to polyvinylidene difluoride membranes (PVDF; Merck Millipore) with a semidry blotter and probed with the following antibodies against: α-tubulin (1:5000; T5168; RRID: AB_330337; Sigma), β-actin (1:5000; A5316; RRID: AB_476743; Sigma), ATF4 (1:1000; WH0000468M1; RRID: AB_1839944; Sigma), CHOP (1:1000; sc-793; RRID:AB_631364; Santa Cruz), GRP78 (1:1000; 3183S; RRID:AB_10695864; Cell Signaling), IRE1α (14C10; 1:1000; 3294S; RRID:AB_823545; Cell Signaling), LC3-I/II (1:1000; 4180S; RRID: AB_2137703; Cell Signaling), p62 (1:1000; ab56416; RRID: AB_945626; Abcam), pACCα (Ser79; 1:1000; 3661S; RRID: AB_330337; Cell Signalling), pAMPKα (Trh172; 1:1000; 2535S; RRID: AB_331250; Cell Signalling), pEIF2α (Ser52; 1:1000; sc-101670; AB_2096507; Santa Cruz), pPERK (Thr980; 1:500; 3179S; RRID:AB_2095853; Cell Signalling) and, UCP1 (1:10,000; ab10983; RRID: AB_2241462; Abcam) as previously described (Dragano et al, 2024; Freire-Agulleiro et al, 2025; Gonzalez-Garcia et al, 2022; Seoane-Collazo et al, 2018). Primary antibodies were incubated with the following secondary antibodies: polyclonal goat anti-rabbit immunoglobulin/HPR (1:5000; Dako; P0448; RRID: AB_2617138) and polyclonal rabbit anti-mouse immunoglobulin/HPR (1:5000; Dako; P0260; RRID: AB_2636929). Autoradiographic films (Fujifilm) were scanned, and band signals were quantified by densitometry using ImageJ-1.44 software (NIH) as reported (Dragano et al, 2024; Freire-Agulleiro et al, 2025; Gonzalez-Garcia et al, 2022; Seoane-Collazo et al, 2018). Values were expressed relative to α-tubulin, or β-actin. Representative images for all proteins are displayed, with all bands in each picture derived from the same gel, although spliced for clarity. A black line was inserted on the immunoblots when samples were loaded on the same gel, but not side by side.

### HPLC-based neurotransmitter quantification

BAT, sWAT, and gWAT samples were dissected on a chilled plate, and immediately flash-frozen in dry ice before being stored at −80 °C until further analysis. The frozen tissue was subsequently homogenized, sonicated, and centrifuged at 14,000×*g* for 20 min at 4 °C. The resulting supernatant fraction was filtered and then injected into a high-performance liquid chromatography (HPLC) system (Shimadzu LC Prominence; Shimadzu Corporation) as shown (Dragano et al, 2024; Fernandez-Gonzalez et al, 2025; Gonzalez-Garcia et al, 2022; Seoane-Collazo et al, 2019).

### Mass‑spectrometry-based quantitative phosphoproteomics analysis

Protein extracts were prepared using a lysis buffer composed of 50 mM Tris-HCl (pH 7.6), 2% SDS, and 10 mM Tris-(2-Carboxyethyl) phosphine (TCEP), supplemented with protease and phosphatase inhibitor cocktails (Complete Mini EDTA-free and phosSTOP, Roche). The samples were heated to boiling for 5 min and then left at room temperature for 20 min to allow for protein solubilization and reduction. Following centrifugation at 15,000×*g* for 15 min, the supernatants were collected. Protein concentrations were measured using the RC DC Protein Assay Kit (Bio-Rad Laboratories). Proteins (120 μg) were digested using the FASP method. Protein extracts were diluted in UA Buffer (7 M urea in 0.1 M Tris-HCl, pH 8.5) and loaded onto 30 kDa centrifugal filter devices (NanoSep 10k Omega, Pall Life Sciences). Proteins were alkylated with 20 mM iodoacetamide in UA for 30 min in the dark, followed by three washes with UA and three washes with 50 mM ammonium bicarbonate. Digestion was carried out overnight at 37 °C using modified trypsin (Promega) in 50 mM ammonium bicarbonate at a protein-to-trypsin ratio of 40:1 (w/w). Trifluoroacetic acid (TFA) was added to a final concentration of 1%, and the peptides were desalted using Oasis-HLB columns (Waters) and dried using a Speed Vac system for further analysis. The tryptic peptides were dissolved in 150 mM Triethylammonium bicarbonate (TEAB) buffer. Peptide concentration was determined by measuring amide bonds with the Direct Detect system (Millipore). Equal amounts of each peptide sample were labeled using TMT 11plex reagents (Thermo Fisher) according to the manufacturer’s protocol with minor modifications. Peptides were labeled with 0.8 mg of TMT reagents reconstituted in 70 μL of acetonitrile. After a 1-h incubation at room temperature, the reaction was stopped with 0.5% TFA and incubated for 10 min. Peptides were combined, concentrated using a Speed Vac, and excess labeling reagents were removed and desalted using Oasis-HLB columns. The samples were dried for further analysis. A fraction of the TMT-labeled sample underwent phosphopeptide enrichment using Ti4+IMAC HP (Immobilized Metal Affinity Chromatography) microparticles (Resyn Biosciences) following the manufacturer’s protocol with slight modifications. Magnetic particles were equilibrated with loading/binding solvent (0.1 M glycolic acid in 80% acetonitrile, 5% TFA) for 5 min and incubated with the reconstituted TMT-labeled sample for 20 min. Bead-bound peptides were washed sequentially with 80% ACN-1% TFA and 10% ACN-0.2% TFA. Phosphopeptides were eluted using 1% ammonium hydroxide, acidified with TFA, desalted on C18 spin microcolumns (Nest Group), and dried in a vacuum centrifuge. A portion of the enriched phosphopeptides was fractionated by using the High pH Reversed-Phase Peptide Fractionation Kit (Pierce). LC-MS analysis of TMT-labeled peptides was performed using a Thermo Scientific EASY-nLC 1000 liquid chromatograph (Thermo Scientific) coupled to a Tribrid Orbitrap mass spectrometer (Orbitrap Fusion, Thermo Scientific). Peptides were suspended in Buffer (0.2% formic acid - 2% acetonitrile), loaded onto a pre-column (PepMap100 C18 LC 75 µm ID, 2 cm, Thermo Scientific), and separated online on a NanoViper PepMap100 C18 LC analytical column (75 µm ID, 50 cm, Thermo Scientific) using a continuous gradient of 5–32% B for 240 min and 32–90% B for 5 min (solvents A and B were 0.1% FA in water and 0.1% FA in acetonitrile, respectively) at a flow rate of 200 nL/min. Each MS run included enhanced FT-resolution spectra (60,000 resolution) in the 400–1500 m/z range, followed by data-dependent MS/MS spectra of the most intense parent ions with charge states 2–4. The AGC target value in the Orbitrap was set to 200,000, with a maximum injection time of 50 ms. Fragmentation was performed at 34% normalized collision energy, targeting 50,000 ions, with 30,000 resolution, 120 ms injection time, and dynamic exclusion set to 40 s. For peptide identification, MS/MS spectra were searched using Proteome Discoverer 2.1 (Thermo Scientific; with the Sequest HT algorithm against the Uniprot database containing all mouse sequences (Uniprot SwissProt and Trembl; September 2018; 53,780 sequences), with decoy sequences generated using DecoyPyrat (Wright and Choudhary, 2016). Parameters included trypsin digestion with up to two missed cleavage sites, precursor and fragment mass tolerances of 2 Da and 0.03 Da, respectively, carbamidomethyl (+57.021) cysteine, and TMT modifications (+229.1629) at peptide N-terminal and Lys residues as fixed modifications. Methionine oxidation (+15.995) and serine/threonine/tyrosine phosphorylation (+79.096) were selected as dynamic modifications. The precursor charge range was set to 2–4. The false discovery rate (FDR) was calculated applying an additional filter for precursor mass tolerance of 12 ppm as described (Bonzon-Kulichenko et al, 2015). Identified peptides had an FDR of 1% or lower. Quantitative analysis of phosphopeptides was performed using the SanXoT program (Perez-Riverol et al, 2025; Rodriguez et al, 2024; Trevisan-Herraz et al, 2019). Quantitative information from TMT reporter intensities was integrated from the spectrum level to the peptide level, and then to the protein level, to quantify the relative abundance changes of each peptide within the corresponding protein. This was done using the WSPP (weighted spectrum, peptide, and protein) statistical model (Navarro et al, 2014) and the Generic Integration Algorithm (GIA) (Garcia-Marques et al, 2016). This model standardizes relative peptide abundances (log2-ratios) expressing the quantitative values in units of standard deviation (*Zpq*) according to their estimated variances. Changes in mean *Zpq* values between groups were analyzed using a two-sided t-test using the Limma approach (Ritchie et al, 2015). RStudio v1.2.1335 was used for the determination of differentially regulated phosphopeptides and generation of volcano plots using the R package EnhancedVolcano, (https://github.com/kevinblighe/EnhancedVolcano), with cutoff values for the adjusted *P* value <0.05 and fold change <−0.5, >0.5.

### Lipidomics

Lipids were extracted from MBH by adding 100 µL of cold isopropanol as extraction solvent. Samples were vortexed previously to five cycles of incubation in liquid nitrogen for 30 s, sonicated for 30 s in a water bath and vortexed for 30 s. Samples were kept for 1 h to enable protein precipitation in the freezer. Subsequently, samples were centrifuged for 10 min at 4 °C and 12,000 rpm. The supernatants were transferred to LC vials. Quality control (QC) samples consisting of pooled samples from each condition were injected at the beginning and periodically through the workflow. Liquid chromatography coupled to mass spectrometry (LC/MS) was performed with a Thermo Scientific Vanquish Horizon UHPLC system interfaced with a Thermo Scientific Orbitrap ID-X Tribrid Mass Spectrometer (Waltham). Lipids were separated by reverse-phase chromatography with an Acquity UPLC C18-RP (ACQUITY UPLC BEH C18 1.7 µM; Waters). Mobile phase A was acetonitrile/water (60:40/v:v) (10 mM ammonium formate), and mobile phase B was isopropanol/acetonitrile (90:10/v:v) (10 mM ammonium formate). Solvent modifiers were used to enhance ionization and to improve the LC resolution in positive ionization mode. Separation was conducted under the following gradient: 0–2 min, 15–30% B; 2–2.5 min, 48% B; 2.5–11 min, 82% B; 11–11.5 min, 99% B; 11.5–12 min, 99% B; 12–12.1 min, 15% B; 12.1–15 min, 15% B. The injection volumes were 2 and 3 µL in positive and negative mode, respectively. Data were collected with the following settings: spray voltage, 3.5 kV and −2.8 kV in positive and negative mode, respectively; sheath gas, 50; auxiliary gas, 10; sweep gas, 1; ion transfer tube temperature, 300 °C; vaporizer temperature, 300 °C; mass range, 150–1800 Da; RF lens, 60%; resolution, 120,000 (MS1); AGC target, 2e5; maximum injection time, 200 ms (MS1), isolation window, 1 Da. An LC/MSMS method using a DDA dynamic Inclusion List (IL) method was used for metabolite identification. The instrument settings were identical to those from the untargeted experiments except for resolution, 15,000; AGC target, 5e4; HCD fragmentation energy in Stepped mode combining 10–20–30–40%; and the injection time that was defined by HERMES (Gine et al, 2021) exported IL in csv format. Metabolite identification was performed by HERMES (Gine et al, 2021) using two strategies: cosine spectral matching using an in-house DB containing MS/MS spectra from MassBankEU, MoNA, HMBD, Riken and NIST23 databases; and using MassFrontiers version 8.0 SR1 (Thermo Scientific) matching against the mzCloud database. Spectra hits with high similarity scores (>0.8) were manually revised to assess correct metabolite identifications, hits were restricted to compounds with formulas present in the HERMES formula database. Lipidomics data processing and variable quantification were performed using Rhermes and XCMS R packages. Data quality were evaluated using PCA to check for outliers and run stability was measured through the coefficient of variation of QC samples’ total intensity. Lipidomic variables were normalized using the total sum of quantified intensity per sample and scaled by log10(x + 1).

### Statistical analysis

Data were expressed as mean ± SEM. Normalized data were presented as percentages of the corresponding control group. Data points outside ±2 standard deviations from the group mean were excluded as outliers from the primary analysis; this criterion was pre-specified before data collection. Statistical significance was determined using two-sided tests, including Student’s *t*-test (for two-group comparisons), two-way ANOVA or mixed-effects analysis (when data were missing), followed by Sidak’s or Bonferroni’s post hoc tests (for >2 groups), or ANCOVA (for calorimetric analyses vs. body weight). Effect sizes were quantified using Cohen’s d. *P* < 0.05 was considered statistically significant. Analyses were performed using Prism 8.0.2 (GraphPad Software).

## Supplementary information

Peer Review File

Dataset EV1

Source data Fig. 1

Source data Fig. 2

Source data Fig. 3

Source data Fig. 4

Source data Fig. 5

Source data Fig. 6

Source data Fig. 7

Source data Fig. 8

Expanded View Figures

## Data Availability

The mass spectrometry proteomics data have been deposited to the ProteomeXchange Consortium (https://www.proteomexchange.org/) via the PRIDE partner repository (https://www.ebi.ac.uk/pride/) with the dataset identifier PXD068034 (https://www.ebi.ac.uk/pride/archive/projects/PXD068034). The lipidomic data from this publication have been deposited to the Zenodo database (https://zenodo.org/) and assigned the identifier 21025207 (https://zenodo.org/records/21025207). The source data of this paper are collected in the following database record: biostudies:S-SCDT-10_1038-S44319-026-00915-0.
