# Supplementary material for: POMC AMPKα1 deletion drives male-specific BAT thermogenesis and obesity resistance
Source: EMBO Rep. 2026 Sep 1;27(20):5779–806. doi: 10.1038/s44319-026-00915-0 (PMC13694704; doi:10.1038/s44319-026-00915-0)
Supplement: Supplementary file 1 [file 44319_2026_915_MOESM11_ESM.pdf]

Expanded View Figures

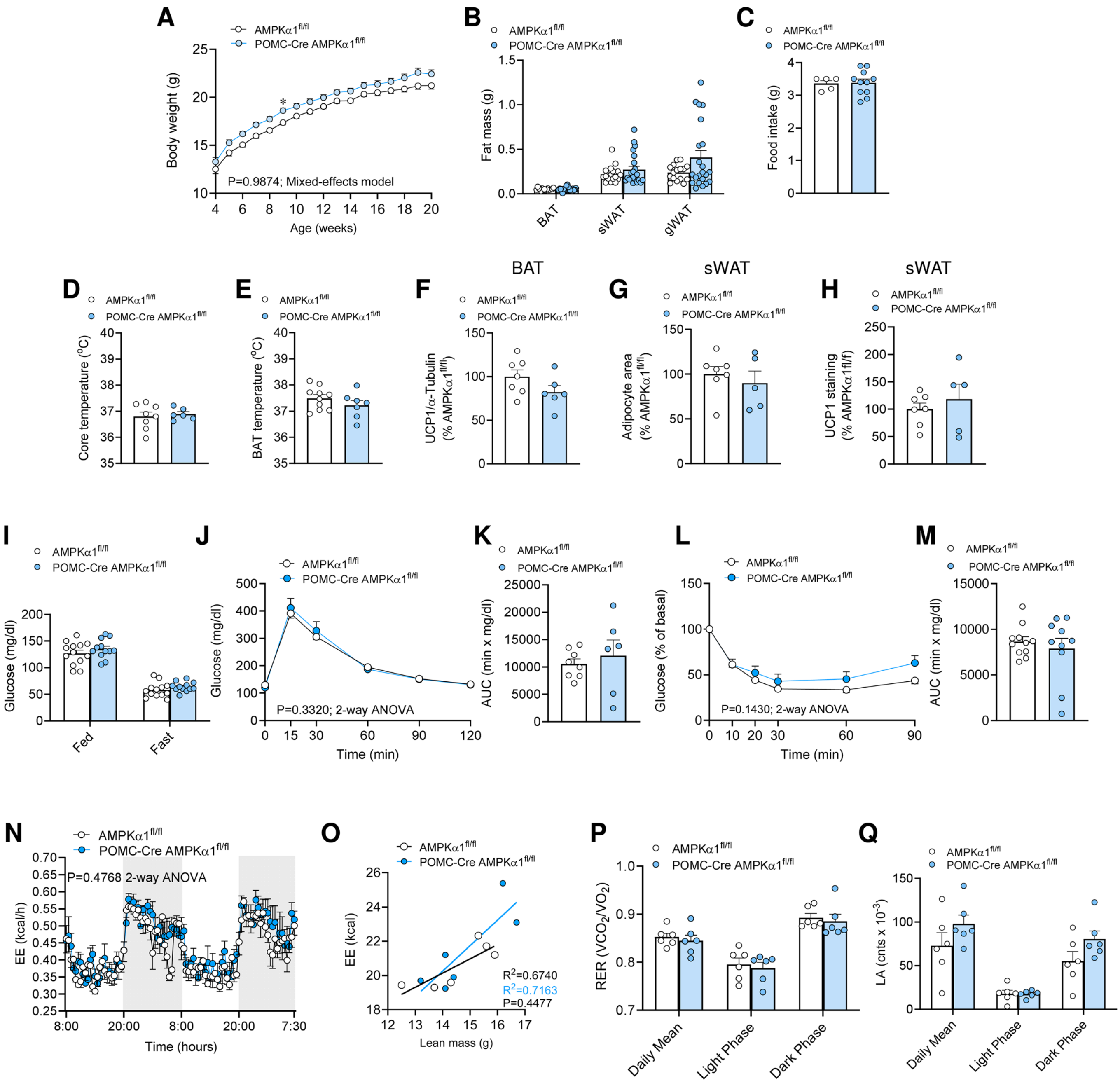

Figure EV1. Effects of loss of AMPKα1 in POMC neurons on energy balance, energy expenditure and glucose homeostasis in female mice fed a chow diet.

(**A–E**) Body weight (**A**; $n = 20$ control and 31 KO), fat depots mass (**B**; $n = 17$ control and 22 KO), and daily food intake (**C**; $n = 5$ control and 11 KO) of control (littermates AMPKα1$^{fl/fl}$) and POMC-Cre AMPKα1$^{fl/fl}$ female mice. (**D-H**) Core body temperature (**D**; $n = 8$ control and 6 KO) BAT temperature (**E**; $n = 10$ control and 7 KO), BAT UCP1 protein levels (**F**; $n = 7$ control and 6 KO), quantification of adipocyte area (**G**; $n = 7$ control and 5 KO) and quantification of UCP1-stained area (**H**; $n = 7$ control and 5 KO) of control (littermates AMPKα1$^{fl/fl}$) and POMC-Cre AMPKα1$^{fl/fl}$ female mice. (**I–M**) Fed and fasted blood glucose levels (**I**; $n = 13$ control and 12KO), glucose tolerance test and area under the curve (AUC) (**J, K**; $n = 8$ control and 6 KO), insulin tolerance test, and AUC (**L, M**; $n = 11$ control and 9 KO) of control (littermates AMPKα1$^{fl/fl}$) and POMC-Cre AMPKα1$^{fl/fl}$ female mice. (**N–Q**) Energy expenditure (EE; **N**; $n = 6$ control and 6 KO), ANCOVA analysis of 48 h total energy expenditure using body weight as a covariate (**O**; $n = 6$ control and 6 KO), respiratory exchange ratio (RER; **P**; $n = 6$ control and 6 KO), locomotor activity (LA; **Q**; $n = 6$ control and 6 KO) of control (littermates AMPKα1$^{fl/fl}$) and POMC-Cre AMPKα1$^{fl/fl}$ female mice. Data were expressed as mean ± SEM. Statistical significance was assessed by mixed-effect model (**A**), two-way ANOVA (**J, L, N**) and Student's *t*-test (**B–I, K, M, P, Q**) and ANCOVA (**O**). *$P < 0.05$ vs. female AMPKα1$^{fl/fl}$.

© The Author(s)

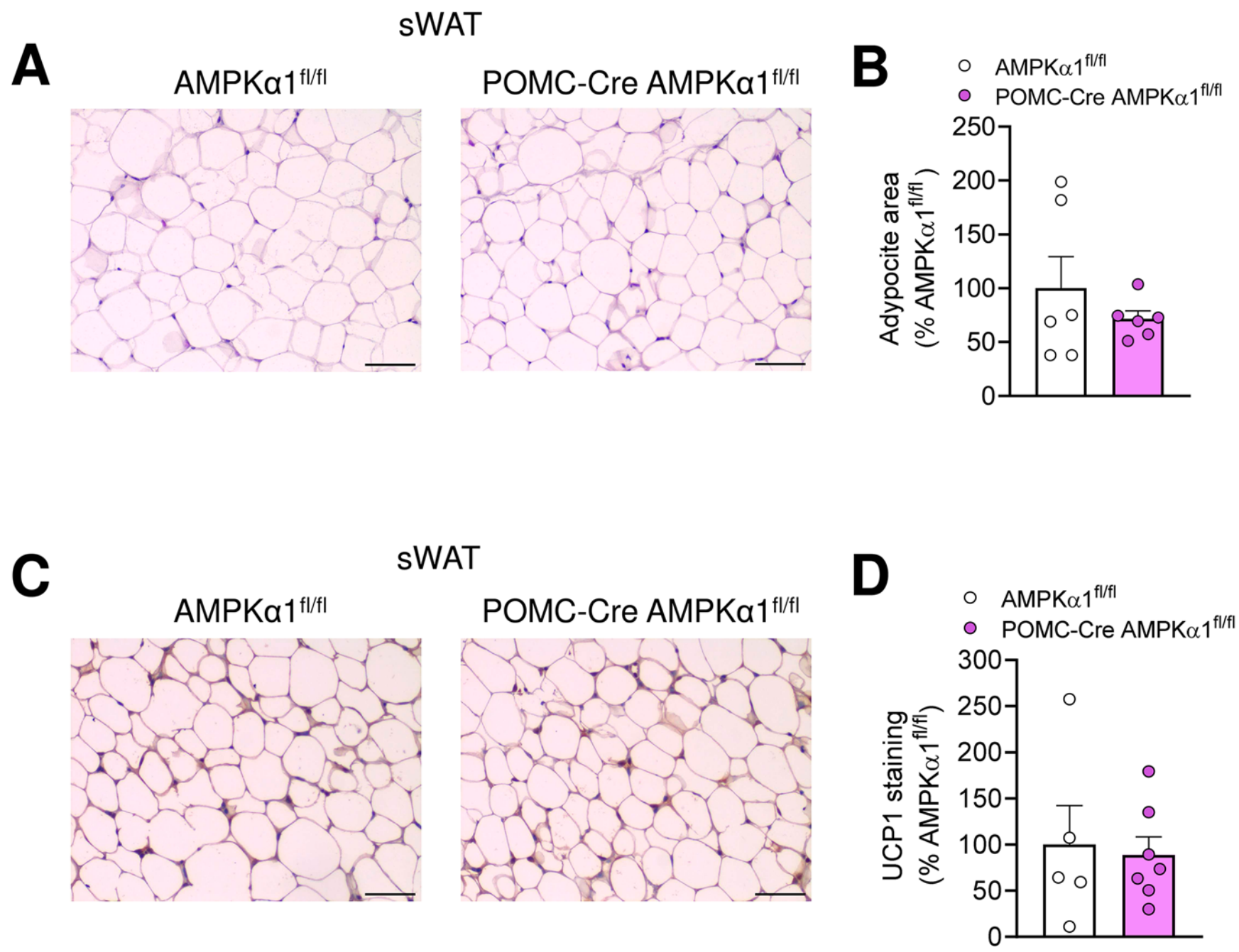

Figure EV2. Effects of loss of AMPKα1 in POMC neurons on WAT in male mice fed a chow diet.

(**A**, **B**) Quantification of the adipocyte area (**A**), and representative subcutaneous white adipose tissue (sWAT) hematoxylin-eosin staining (**B**; $n = 6$ control and 6 KO) of AMPKα1$^{fl/fl}$ mice, and POMC-Cre AMPKα1$^{fl/fl}$ mice. Scale bar 50 µm. The mean value of each animal was obtained from 3 photos of each tissue. (**C**, **D**) Quantification of UCP1-stained area (**C**) and representative sWAT immunohistochemistry with anti-UCP1 antibody showing UCP1 staining (**D**; $n = 5$ control and 7 KO) of AMPKα1$^{fl/fl}$ mice, and POMC-Cre AMPKα1$^{fl/fl}$ mice. Scale bar 50 µm. Data were expressed as mean ± SEM. Statistical significance was assessed by Student's *t*-test (**B**, **D**).

 © The Author(s)

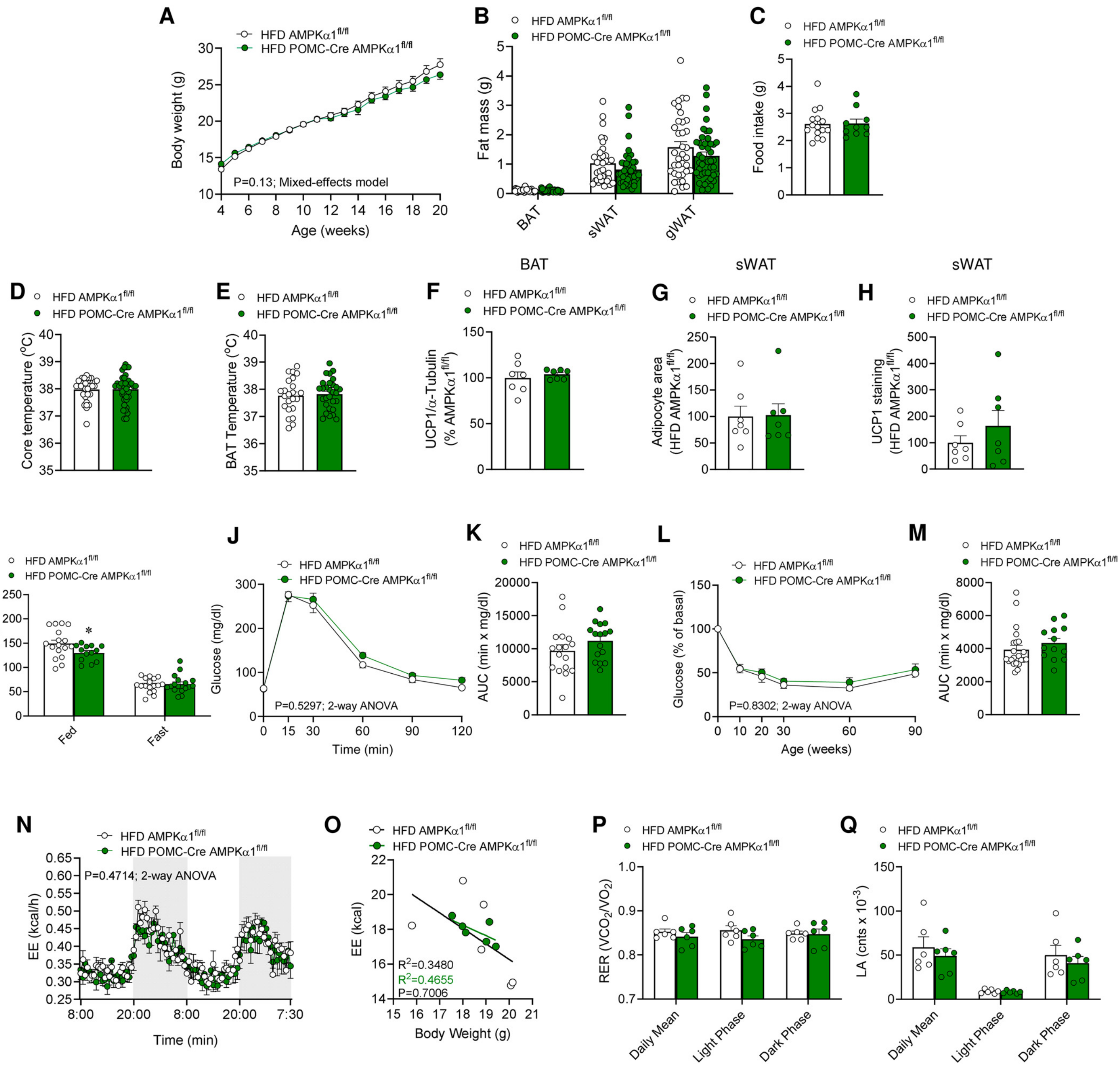

Figure EV3. Effects of loss of AMPKα1 in POMC neurons on energy balance, energy expenditure and glucose homeostasis in female mice fed a HFD.

(**A–E**) Body weight (**A**; $n = 31$ control and 42 KO), fat depot mass (**B**; $n = 36$ control and 43 KO), and daily food intake (**C**; $n = 15$ control and 10 KO) of control (littermates AMPKα1$^{fl/fl}$) and POMC-Cre AMPKα1$^{fl/fl}$ female mice in HFD. (**D–H**) Core body temperature (**D**; $n = 28$ control and 38 KO) BAT temperature (**E**; $n = 23$ control and 29 KO), BAT UCP1 protein levels (**F**; $n = 7$ control and 7 KO), quantification of adipocyte area (**G**; $n = 7$ control and 7 KO) and quantification of UCP1-stained area (**H**; $n = 7$ control and 7 KO) of control (littermates AMPKα1$^{fl/fl}$) and POMC-Cre AMPKα1$^{fl/fl}$ female mice in HFD. (**I–M**) Fed and fasted blood glucose levels (**I**; $n = 17$ control and 13-17 KO; $P = 0.047664$ for fed), glucose tolerance test and area under the curve (AUC) (**J**, **K**; $n = 17$ control and 16 KO), insulin tolerance test, and area under the curve (**L**, **M**; $n = 22$ control and 13 KO) of control (littermates AMPKα1$^{fl/fl}$) and POMC-Cre AMPKα1$^{fl/fl}$ female mice in HFD. (**N–Q**) Energy expenditure (EE; **N**; $n = 6$ control and 6 KO), ANCOVA analysis of 48 h total energy expenditure using body weight as a covariate (**O**; $n = 6$ control and 6 KO), respiratory exchange ratio (RER; **P**; $n = 6$ control and 6 KO), locomotor activity (LA; **Q**; $n = 6$ control and 6 KO) of control (littermates AMPKα1$^{fl/fl}$) and POMC-Cre AMPKα1$^{fl/fl}$ female mice in HFD. Data are expressed as mean ± SEM. Statistical significance was assessed by Mixed-effect model (**A**), two-way ANOVA (**J**, **L**, **N**), Student's *t*-test (**B–I**, **K**, **M**, **P**, **Q**) and ANCOVA (**O**). *$P < 0.05$ vs. HFD female AMPKα1$^{fl/fl}$.

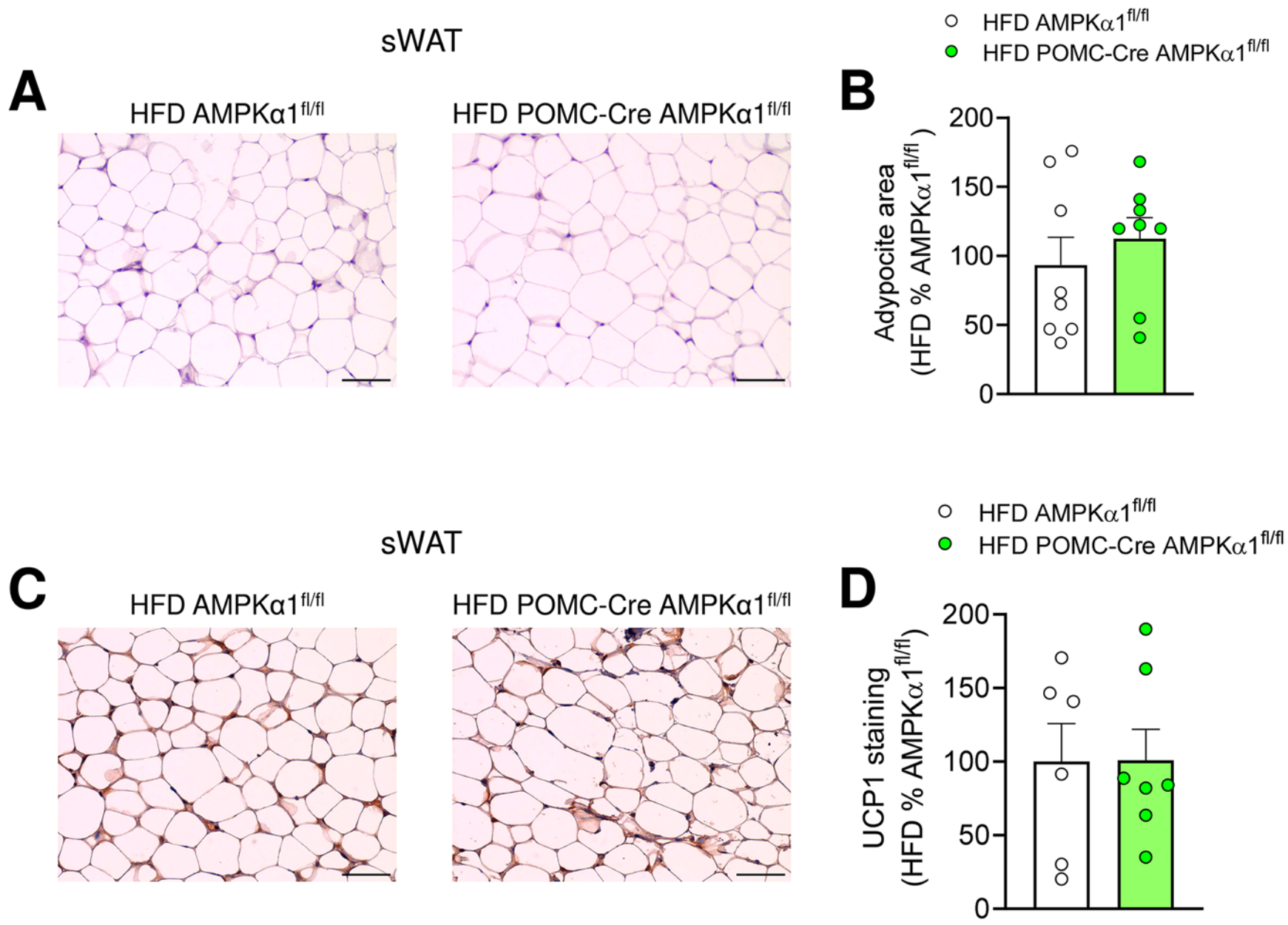

Figure EV4. Effects of loss of AMPKα1 in POMC neurons on WAT in male mice fed a HFD.

(**A**, **B**) Quantification of the adipocyte area (**A**), and representative subcutaneous white adipose tissue (sWAT) hematoxylin-eosin staining (**B**; $n = 8$ control and 8 KO) of AMPKα1$^{fl/fl}$ mice, and POMC-Cre AMPKα1$^{fl/fl}$ mice fed a HFD. Scale bar 50 µm. The mean value of each animal was obtained from three photos of each tissue. (**C**, **D**) Quantification of UCP1-stained area (**C**) and representative sWAT immunohistochemistry with anti-UCP1 antibody showing UCP1 staining (**D**; $n = 7$ control and 6 KO) of AMPKα1$^{fl/fl}$ mice, and POMC-Cre AMPKα1$^{fl/fl}$ mice fed a HFD. Scale bar 50 µm. Data are expressed as mean ± SEM. Statistical significance was assessed by Student's *t*-test (**B**, **D**).

 © The Author(s)

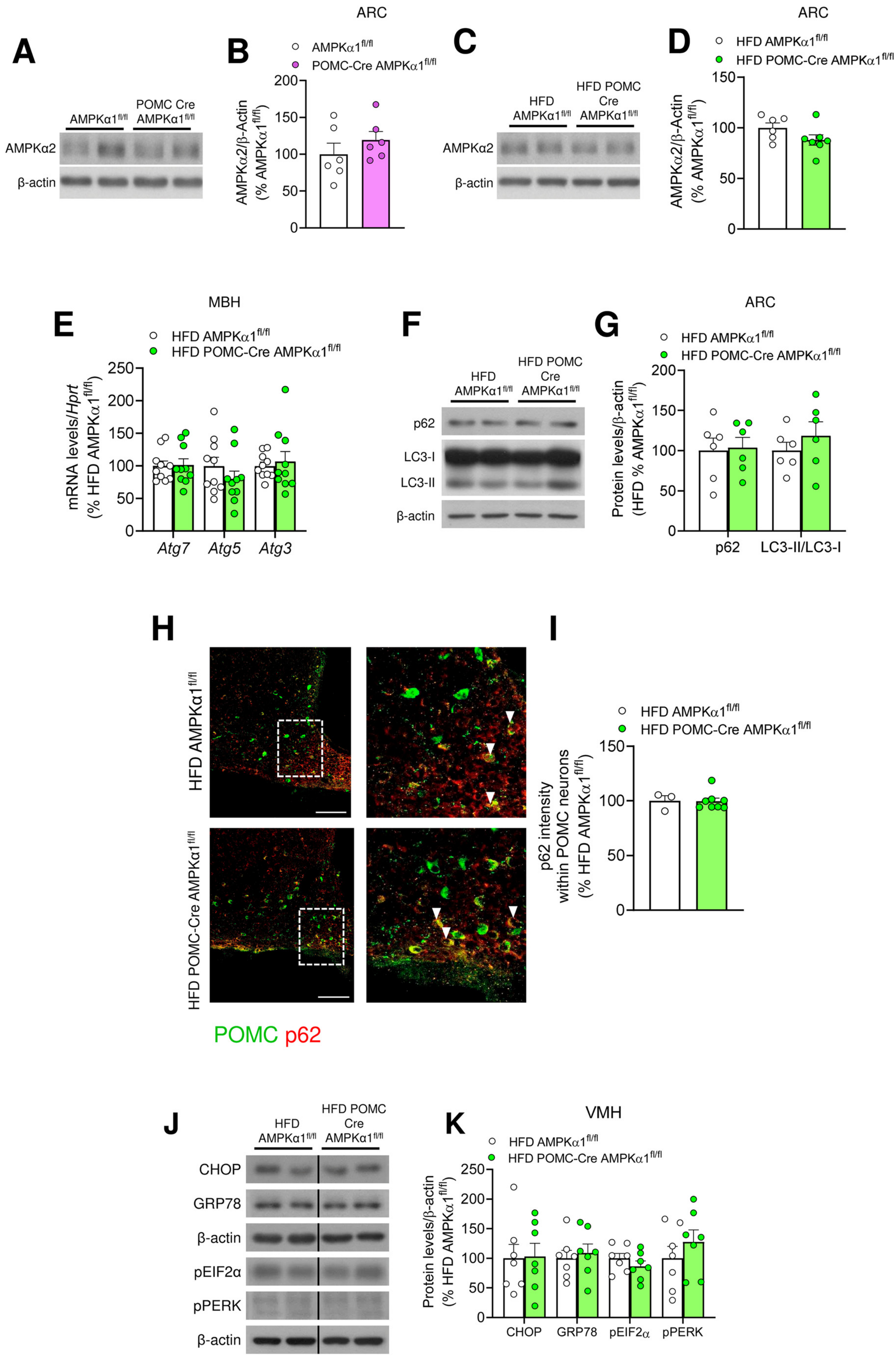
A
AMPKα1fl/fl
POMC Cre AMPKα1fl/fl
AMPKα2
β-actin
B
ARC
AMPKα1fl/fl
POMC-Cre AMPKα1fl/fl
AMPKα2/β-Actin (% AMPKα1fl/fl)
200
150
100
50
0
C
HFD AMPKα1fl/fl
HFD POMC Cre AMPKα1fl/fl
AMPKα2
β-actin
D
ARC
HFD AMPKα1fl/fl
HFD POMC-Cre AMPKα1fl/fl
AMPKα2/β-Actin (% AMPKα1fl/fl)
150
100
50
0
E
MBH
HFD AMPKα1fl/fl
HFD POMC-Cre AMPKα1fl/fl
mRNA levels/Hprt (% HFD AMPKα1fl/fl)
250
200
150
100
50
0
Atg7
Atg5
Atg3
F
HFD AMPKα1fl/fl
HFD POMC Cre AMPKα1fl/fl
p62
LC3-I
LC3-II
β-actin
G
ARC
HFD AMPKα1fl/fl
HFD POMC-Cre AMPKα1fl/fl
Protein levels/β-actin (HFD % AMPKα1fl/fl)
200
150
100
50
0
p62
LC3-II/LC3-I
H
HFD AMPKα1fl/fl
HFD POMC-Cre AMPKα1fl/fl
POMC p62
I
HFD AMPKα1fl/fl
HFD POMC-Cre AMPKα1fl/fl
p62 intensity within POMC neurons (% HFD AMPKα1fl/fl)
150
100
50
0
J
HFD AMPKα1fl/fl
HFD POMC Cre AMPKα1fl/fl
CHOP
GRP78
β-actin
pEIF2α
pPERK
β-actin
K
VMH
HFD AMPKα1fl/fl
HFD POMC-Cre AMPKα1fl/fl
Protein levels/β-actin (% HFD AMPKα1fl/fl)
250
200
150
100
50
0
CHOP
GRP78
pEIF2α
pPERK

Figure EV5. Effects of loss of AMPKα1 in POMC neurons on hypothalamic AMPKα2, autophagy and ER stress.

(**A**, **B**) Representative ARC AMPKα2 western blot images (**A**) and AMPKα2 protein levels in the ARC (**B**; $n = 6$ control and 6 KO) of control (littermates AMPKα1$^{fl/fl}$) and POMC-Cre AMPKα1$^{fl/fl}$ male mice. (**C**, **D**) Representative ARC AMPKα2 western blot images (**C**) and AMPKα2 protein levels in the ARC (**D**; $n = 6$ control and 7 KO) of control (littermates AMPKα1$^{fl/fl}$) and POMC-Cre AMPKα1$^{fl/fl}$ male mice in HFD. (**E**) mRNA expression of autophagy-related proteins in the MBH ($n = 10$ control and 10 KO) of control (littermates AMPKα1$^{fl/fl}$) and POMC-Cre AMPKα1$^{fl/fl}$ male mice fed a HFD. (**F**, **G**) Representative ARC autophagy markers western blot images (**F**) and autophagy markers protein levels in the ARC (**G**; $n = 6$ control and 6 KO) of control (littermates AMPKα1$^{fl/fl}$) and POMC-Cre AMPKα1$^{fl/fl}$ male mice fed a HFD. (**H**, **I**) Representative confocal images from the ARC of HFD AMPKα1$^{fl/fl}$ mice and HFD POMC-Cre AMPKα1$^{fl/fl}$ mice showing p62 (red) and POMC (green) immunofluorescent staining (white arrowheads indicate cells coexpressing POMC and p62; scale bar 100 μm; zoomed images come from the squared region in the first image, depicting details of POMC neurons colocalization with p62) and quantification of the p62 intensity within POMC neurons (**I**; $n = 3$ control and 7 KO) of control (littermates AMPKα1$^{fl/fl}$) and POMC-Cre AMPKα1$^{fl/fl}$ male mice in HFD. (**J**, **K**) Representative VMH ER stress pathway western blot images (**J**) and ER stress pathway protein levels in the VMH (**K**; $n = 7$ control and 6-7 KO) of control (littermates AMPKα1$^{fl/fl}$) and POMC-Cre AMPKα1$^{fl/fl}$ male mice fed a HFD. In the western blot analyses, β-actin was used as control. A black line between the immunoblots depicts samples loaded in the same gel, but not next to each other. Data were expressed as mean ± SEM. Statistical significance was assessed by Student's *t*-test (**B**, **D**, **E**, **G**, **I**, **K**).

 © The Author(s)
