# Supplementary material for: POMC AMPKα1 deletion drives male-specific BAT thermogenesis and obesity resistance
Source: EMBO Rep. 2026 Sep 1;27(20):5779–806. doi: 10.1038/s44319-026-00915-0 (PMC13694704; doi:10.1038/s44319-026-00915-0)
Supplement: Supplementary file 2 [file 44319_2026_915_MOESM1_ESM.pdf]

EMBO reports

POMC AMPKα1 deletion drives male-specific BAT thermogenesis and obesity resistance

Óscar Freire-Agulleiro, Marcos Rios Garcia, Vitor Ferreira, Patricia Seoane-Collazo, Juan Roa, Ánxela Estévez-Salguero, Alicia Otero-Rey, Jordi Capellades, Francisco Ruiz-Pino, Pablo Garrido-Gil, Vincent Simon, Jean-Charles Nicolas, Juan Antonio López, Cristina Contreras, Laura Liñares-Pose, Francisco González, José Labandeira-García, Jesús Vázquez, Carlos Diéguez, Ruben Nogueiras, Daniela Cota, Oscar Yanes, Guadalupe Sabio, Manuel Tena-Sempere, Carmelo Quarta, Ismael González-García, and Miguel Lopez

Corresponding author(s): Miguel Lopez (m.lopez@usc.es) , Ismael González-García (ismael.gonzalez@usc.es)

Review Timeline:

Submission Date:	12th Jun 25
Editorial Decision:	11th Jul 25
Revision Received:	19th Jun 26
Editorial Decision:	10th Jul 26
Revision Received:	20th Jul 26
Accepted:	3rd Aug 26

Editor: Deniz Senyilmaz Tiebe and Kurt Weir

Transaction Report: This manuscript was transferred to EMBO reports following peer review at The EMBO Journal.

(Note: With the exception of the correction of typographical or spelling errors that could be a source of ambiguity, letters and reports are not edited. Depending on transfer agreements, referee reports obtained elsewhere may or may not be included in this compilation. Referee reports are anonymous unless the Referee chooses to sign their reports.)

1st Editorial Decision 11th Jul 2025

Dear Prof. Lopez,

Thank you for transferring your manuscript to EMBO Reports, which was now seen by two referees, whose reports are copied below.

Referees express interest in the proposed role of AMPKα1 in POMC neurons in regulation of BAT activity. However, they also raise important overlapping concerns. In particular,

1. All experiments are done on male mice, which does not address the sex specificity of the findings (referee #1, point 4; referee #2, point 3). I discussed this point further with the referees, who find that redoing all experiments in female mice is naturally not expected. However, some of the key findings need to be tested in female mice as well and the results need to be carefully discussed.

2. The effect of AMPKα1 depletion in POMC neurons on BAT activity (referee #1, point 5; referee #2, point 1), body composition (referee #2, point 2) and autophagic flux (referee #1, point 3) need to be characterized in more depth.

Please contact me if you have questions or comments regarding any of these points or the revision for further discussion (also by video chat).

In case you can satisfactorily address these concerns, we would like to invite you to revise your manuscript with the understanding that the referee concerns (as in their reports) must be fully addressed and their suggestions taken on board. Please address all referee concerns in a complete point-by-point response. Acceptance of the manuscript will depend on a positive outcome of a second round of review. It is EMBO reports policy to allow a single round of major experimental revision only and acceptance or rejection of the manuscript will therefore depend on the completeness of your responses included in the next, final version of the manuscript.

We realize that it is difficult to revise to a specific deadline. In the interest of protecting the conceptual advance provided by the work, we recommend a revision within 3 months. Please discuss the revision progress ahead of this time with me if you require more time to complete the revisions, or if you have questions or comments regarding the revision (also by video chat).

IMPORTANT NOTE: we perform an initial quality control of all revised manuscripts before re-review. Your manuscript will FAIL this control and the handling will be DELAYED if the following APPLIES:
1. A data availability section providing access to data deposited in public databases is missing (where applicable).
2. Your manuscript contains statistics and error bars based on n=2. Please use scatter plots in these cases.

You can submit the revision either as a Scientific Report or as a Research Article. For Scientific Reports, the revised manuscript can contain up to 5 main figures and 5 Expanded View figures, and it should not exceed 27000 characters. If the revision leads to a manuscript with more than 5 main figures it will be published as a Research Article. In this case the Results and Discussion section should be separate. If a Scientific Report is submitted, these sections have to be combined. This will help to shorten the manuscript text by eliminating some redundancy that is inevitable when discussing the same experiments twice. In either case, all materials and methods should be included in the main manuscript file.

When submitting your revised manuscript, please carefully review the instructions that follow below. Failure to include requested items will delay the evaluation of your revision.

1) a .docx formatted version of the manuscript text (including legends for main figures, EV figures and tables). Please make sure that the changes are highlighted to be clearly visible.

2) individual production quality figure files as .eps, .tif, .jpg (one file per figure). See https://wol-prod-cdn.literatumonline.com/pb-assets/embo-site/EMBOPress_Figure_Guidelines_061115-1561436025777.pdf for more info on how to prepare your figures.

3) We replaced Supplementary Information with Expanded View (EV) Figures and Tables that are collapsible/expandable online. A maximum of 5 EV Figures can be typeset. EV Figures should be cited as 'Figure EV1, Figure EV2" etc... in the text and their respective legends should be included in the main text after the legends of regular figures.

- For the figures that you do NOT wish to display as Expanded View figures, they should be bundled together with their legends in a single PDF file called *Appendix*, which should start with a short Table of Content. Appendix figures should be referred to in the main text as: "Appendix Figure S1, Appendix Figure S2" etc. See detailed instructions regarding expanded view here: <https://www.embopress.org/page/journal/14693178/authorguide#expandedview>

- Additional Tables/Datasets should be labeled and referred to as Table EV1, Dataset EV1, etc. Legends have to be provided in

a separate tab in case of .xls files. Alternatively, the legend can be supplied as a separate text file (README) and zipped together with the Table/Dataset file.

4) a .docx formatted letter INCLUDING the reviewers' reports and your detailed point-by-point responses to their comments. As part of the EMBO publication's Transparent Editorial Process, EMBO reports publishes online a Review Process File (RPF) to accompany accepted manuscripts. This File will be published in conjunction with your paper and will include the referee reports, your point-by-point response and all pertinent correspondence relating to the manuscript.
https://www.embopress.org/page/journal/14693178/authorguide#transparentprocess
You are able to opt out of this by letting the editorial office know (emboreports@embo.org). If you do opt out, the Review Process File link will point to the following statement: "No Review Process File is available with this article, as the authors have chosen not to make the review process public in this case."

5) a complete author checklist, which you can download from our author guidelines
https://www.embopress.org/page/journal/14693178/authorguide. Please insert information in the checklist that is also reflected in the manuscript. The completed author checklist will also be part of the RPF.

6) Please note that all corresponding authors are required to supply an ORCID ID for their name upon submission of a revised manuscript (<https://orcid.org/>). Please find instructions on how to link your ORCID ID to your account in our manuscript tracking system in our Author guidelines
<https://www.embopress.org/page/journal/14693178/authorguide#authorshipguidelines>

7) Before submitting your revision, primary datasets produced in this study need to be deposited in an appropriate public database (see https://www.embopress.org/page/journal/14693178/authorguide#datadeposition). Please remember to provide a reviewer password if the datasets are not yet public. The accession numbers and database should be listed in a formal "Data Availability" section placed after Materials & Method (see also
https://www.embopress.org/page/journal/14693178/authorguide#datadeposition). Please note that the Data Availability Section is restricted to new primary data that are part of this study. * Note - All links should resolve to a page where the data can be accessed. *
If your study has not produced novel datasets, please mention this fact in the Data Availability Section.

8) At EMBO Press we ask authors to provide source data for the main manuscript figures. You will receive a separate email with instructions for providing source data with your revised manuscript, including how to upload and organize the files.

Additional information on source data and instruction on how to label the files are available:
https://www.embopress.org/page/journal/14693178/authorguide#sourcedata

9) Our journal encourages inclusion of *data citations in the reference list* to directly cite datasets that were re-used and obtained from public databases. Data citations in the article text are distinct from normal bibliographical citations and should directly link to the database records from which the data can be accessed. In the main text, data citations are formatted as follows: "Data ref: Smith et al, 2001" or "Data ref: NCBI Sequence Read Archive PRJNA342805, 2017". In the Reference list, data citations must be labeled with "[DATASET]". A data reference must provide the database name, accession number/identifiers and a resolvable link to the landing page from which the data can be accessed at the end of the reference. Further instructions are available at http://www.embopress.org/page/journal/14693178/authorguide#referencesformat

10) Regarding data quantification (see Figure Legends:
https://www.embopress.org/page/journal/14693178/authorguide#figureformat)

The following points must be specified in each figure legend:

- the name of the statistical test used to generate error bars and P values,

- the number (n) of independent experiments (please specify technical or biological replicates) underlying each data point,

- the nature of the bars and error bars (s.d., s.e.m.),

- If the data are obtained from n Program fragment delivered error ``Can't locate object method "less" via package "than" (perhaps you forgot to load "than"?) at //ejpvfs23/sites23b/embor_www/letters/embor_decision_revise_and_review.txt line 56.' 2, use scatter blots showing the individual data points.

Discussion of statistical methodology can be reported in the materials and methods section, but figure legends should contain a basic description of n, P and the test applied.

- Please also include scale bars in all microscopy images.

11) The journal requires a statement specifying whether or not authors have competing interests (defined as all potential or actual interests that could be perceived to influence the presentation or interpretation of an article). In case of competing interests, this must be specified in your disclosure statement. Further information: https://www.embopress.org/competing-interests

12) Please also note our reference format:
http://www.embopress.org/page/journal/14693178/authorguide#referencesformat

13) All Materials and Methods need to be described in the main text using our 'Structured Methods' format, which is required for all research articles. According to this format, the Methods section includes a Reagents and Tools Table (listing key reagents, experimental models, software and relevant equipment and including their sources and relevant identifiers) followed by a Methods and Protocols section describing the methods using a step-by-step protocol format. The aim is to facilitate adoption of the methodologies across labs. More information on how to adhere to this format as well as a downloadable template (.docx) for the Reagents and Tools Table can be found in our author guidelines:
https://www.embopress.org/page/journal/14693178/authorguide#structuredmethods.

An example of a Method paper with Structured Methods can be found here:
https://www.embopress.org/doi/10.15252/msb.20178071.

We would also welcome the submission of cover suggestions, or motifs to be used by our Graphics Illustrator in designing a cover.

I look forward to seeing a revised version of your manuscript when it is ready. Please let me know if you have questions or comments regarding the revision.

Kind regards,

Deniz Senyilmaz Tiebe

Deniz Senyilmaz Tiebe, PhD
Senior Scientific Editor
EMBO Reports

Referee #1:

This study by Freire-Agulleiro et al. demonstrates that deletion of AMPKα1 in hypothalamic POMC neurons protects against diet-induced obesity via a mechanism that involves enhanced BAT thermogenesis and reduced hypothalamic ER stress. Using POMC-Cre AMPKα1fl/fl mice, the authors demonstrate that the metabolic benefits are accompanied by distinct phosphoproteomic changes in the ARC. The study addresses an important gap in understanding the specific and subunit-dependent roles of AMPK in POMC neurons in the context of energy homeostasis and obesity.
However, there are some major limitations in the study:
1. For POMC-Cre driver, developmental compensation or early circuit alterations cannot be ruled out. An inducible model would be preferable, or at least discussed in the limitations section.
2. The study lacks direct validation of specific candidate phosphoproteins or signaling pathways. Some functional assays for the mechanistic connection would be helpful.
3. Autophagic flux is dynamic and might require more sensitive assessments.
4. All experiments are done in male mice. Given the known sexual dimorphism in hypothalamic control of energy balance and AMPK signaling, the absence of female mice is a limitation.
5. The conclusion that POMC-AMKPα1 KO increases BAT thermogenesis via enhanced SNS outflow is indirect.

Minor
• AMPKα2 compensates functionally in KO mice, can be shown in the supplementary figure.
• Supplementary figures for adipocyte area, UCP1 staining, and autophagy markers, but not fully referenced in the main text.
• Include effect sizes and sample sizes in figure legends.

Referee #2:

In this manuscript, the authors investigated the metabolic consequences of deleting AMPKa1 in POMC neurons. While this results in no apparent phenotype on a standard chow diet, upon high fat feeding, knockout animals show reduced weight gain. This appears to be driven by increased energy expenditure, as food intake is unchanged. The authors argue that this phenotype is the result of increased BAT thermogenesis, due to small but statistically significant increases in BAT temperature and UCP1 protein. They then go on to investigate molecular mechanisms and show that AMPKa1 deletion in POMC neurons leads to rewiring of the phosphoproteome in the arcuate nucleus. Moreover, knockout animals show a more beneficial ER stress response and decreased expression of ceramide biosynthesis transcripts.

Overall, this is an interesting study that adds to the body of knowledge from this group and others on the role of hypothalamic AMPK signaling in coordinating whole-body metabolism. However, additional experiments are needed to fully support the claim that the phenotype measured here is due to BAT activation.

Main Points:

1. To more fully substantiate the argument that knockout mice have increased BAT activity, a more complete characterization of the brown fat is needed. This should include mRNA analysis of Ucp1 and other thermogenic genes in chow and HFD (Pgc1a, Dio2). Additionally, to help clarify whether this apparent increase in BAT activity is due to diet-induced thermogenesis or a broader increase in BAT activity, the authors should do cold-tolerance tests in control and knockout animals. Body temperature and BAT temperature should be measured before and after a cold challenge, and upon sacrifice, thermogenic gene mRNAs should be measured.

2. In Figure 4, it is clear that the knockout mice gain significantly less weight on high fat diet. However, in Figure 4B, there is no difference in the weights of the major fat depots. This raises the possibility that the knockouts actually have decreased muscle mass. This needs to be addressed with body composition analysis.

3. From the methods, it appears that all the studies were done in male mice. The authors should either extend their studies to female mice, or they need to state explicitly in the text that they only studied male mice and thus do not know if the same findings hold in females.

Minor Points:

1. For the metabolic characterization (GTT, ITT, indirect calorimetry), the authors need to state explicitly what time points they were performed.

2. In Figure 8, the argument about ceramides would be more compelling if they actually measured ceramide levels.

3. The discussion is very long. It would be more readable if it was shortened.

Rebuttal EMBOR-2025-62113-T

EDITOR

Overall comment: *Thank you for transferring your manuscript to EMBO Reports, which was now seen by two referees, whose reports are copied below. Referees express interest in the proposed role of AMPKα1 in POMC neurons in regulation of BAT activity. However, they also raise important overlapping concerns. In particular:*

Response: We thank the Editor and the Reviewers for their constructive evaluation of our manuscript and for the opportunity to revise it. We are pleased that they recognize the interest of the proposed role of AMPKα1 in POMC neurons in the regulation of brown adipose tissue (BAT). In the revised version, we have performed additional experiments and clarifications to address all concerns raised. Altogether, **the new version of the manuscript includes 82 new additional datasets,** greatly strengthening the methodological solidity and conceptual significance of our work. Please note that throughout this Rebuttal, **all Figure numbering refers to the new version of the manuscript**

Editor's comment 1: *All experiments are done on male mice, which does not address the sex specificity of the findings (referee #1, point 4; referee #2, point 3). I discussed this point further with the referees, who find that redoing all experiments in female mice is naturally not expected. However, some of the key findings need to be tested in female mice as well and the results need to be carefully discussed.*

Response: We thank the Editor and Referees for raising this important point regarding sex specificity in hypothalamic AMPK signaling. In response, we have performed comprehensive phenotypic characterization in female mice across all major experiments, including body weight curves, adiposity, food intake, body and BAT temperatures, BAT UCP1 expression, browning of WAT, glucose/insulin tolerance test, energy expenditure (EE), respiratory exchange ratio (RER) and locomotor activity (LA) **(Figures EV2A-Q and EV4A-Q).** Our data reveal a striking sexual dimorphism: like males, female POMC-AMPKα1 KO mice exhibit no metabolic alterations on standard chow diet **(Figure EV2A-Q)**. However, in marked contrast to the robust HFD resistance observed in males, HFD-fed females showed no major differences in body weight gain, fat mass accumulation, or metabolic phenotyping parameters compared to controls **(Figure EV4A-Q).** This sex-specific HFD response indicates that AMPKα1 in POMC neurons exerts diet-dependent, sexually dimorphic control over energy balance, likely reflecting gonadal hormone modulation of hypothalamic nutrient sensing. We thank the Editor and Reviewers for highlighting this critical aspect, which has revealed an important new conclusion regarding sex differences in POMC neuronal AMPKα1 function.

Editor's comment 2: *The effect of AMPKα1 depletion in POMC neurons on BAT activity (referee #1, point 5; referee #2, point 1), body composition (referee #2, point 2) and autophagic flux (referee #1, point 3) need to be characterized in more depth.*

Response: We thank the Editor and Referees for raising these important mechanistic questions, which we have comprehensively tried to address in the revised manuscript.

1. **For BAT activity,** we have deepened the characterization by direct quantification of norepinephrine (NE) levels in BAT tissue via HPLC from both chow- and HFD-fed mice **(Figures 2I and 3I),** confirming elevated sympathetic outflow in POMC-AMPKα1 KO mice in HFD conditions. Additionally, we performed cold-tolerance challenges (4°C for 6h) measuring core body temperature, BAT temperature, and tail temperature **(Figure 4A-J),** alongside indirect calorimetry during HFD transition tracking oxygen consumption (VO_2), energy expenditure (EE), and respiratory exchange ratio (RER) **(Figure 4K-M).** These studies revealed diet-independent BAT activation without enhanced adaptive thermogenic capacity, refining our model of basal BAT activation.

2. **Regarding body composition,** and to address potential lean mass changes, we performed nuclear magnetic resonance (NMR) on HFD-fed POMC-AMPKα1 KO and control mice, revealing no differences in lean body mass between genotypes (**Rebuttal Figure R2**).
3. **For autophagy,** we specifically quantified p62 levels in POMC neurons from WT and KO mice in HFD conditions **(Figure EV5H, I),** complementing prior autophagy-related mRNA **(Figure EV5E)** and protein analyses **(Figure EV5F, G).** Consistent with these datasets, no p62 differences were detected (**Figure EV5H, I**), indicating that autophagy does not contribute to the observed phenotype.

These extensive new characterizations directly address all Referee's concerns and have been integrated into the revised Results and Discussion.

We sincerely thank the Editor for the outstanding and insightful comments, which have greatly contributed to improving the clarity and rigor of our manuscript.

REVIEWER#1

Overall comment: *This study by Freire-Agulleiro et al. demonstrates that deletion of AMPKα1 in hypothalamic POMC neurons protects against diet-induced obesity via a mechanism that involves enhanced BAT thermogenesis and reduced hypothalamic ER stress. Using POMC-Cre AMPKα1fl/fl mice, the authors demonstrate that the metabolic benefits are accompanied by distinct phosphoproteomic changes in the ARC. The study addresses an important gap in understanding the specific and subunit-dependent roles of AMPK in POMC neurons in the context of energy homeostasis and obesity. However, there are some major limitations in the study:*

Response: We sincerely thank Reviewer #1 for the outstanding and insightful comments, which have significantly helped us to improve the clarity, robustness, and overall quality of the manuscript. Please note that throughout this Rebuttal, **all Figure numbering refers to the new version of the manuscript**

Reviewer#1's comment 1: *For POMC-Cre driver, developmental compensation or early circuit alterations cannot be ruled out. An inducible model would be preferable or at least discussed in the limitations section.*

Response: We thank the Reviewer for this insightful comment. We fully agree that the constitutive POMC-Cre driver introduces the possibility of developmental compensation or early circuit alterations that could influence the observed phenotypes, representing a recognized limitation of non-inducible Cre systems. While an inducible POMC-Cre^{ERT2} model would provide precise temporal control of recombination to circumvent these developmental confounds (allowing adult-onset deletion and clearer attribution of effects to mature neuronal AMPKα1 signaling) our study intentionally employed the widely used constitutive POMC-Cre line to ensure direct comparability with the extensive preexisting literature on POMC neuronal manipulations, including multiple seminal studies of metabolic regulation that established this driver as a standard tool. This choice facilitates integration of our subunit-specific findings within the established field framework, despite the inherent developmental caveat. Although we could not detect differences in GABAergic and glutamatergic POMC neurons subpopulations neither the number of POMC neurons in our analyses **(Figure 6A-C)** suggesting that a profound deleterious effect on these neurons is unlikely, we recognize Reviewer's point. Thus, we have now included a detailed discussion of this limitation in the revised manuscript explicitly acknowledging that early developmental recombination could contribute to some observed outcomes and outlining the strengths and trade-offs of constitutive vs. inducible approaches in hypothalamic circuit studies.

Reviewer#1's comment 2: *The study lacks direct validation of specific candidate phosphoproteins or signaling pathways. Some functional assays for the mechanistic connection would be helpful.*

Response: We thank the Reviewer for this insightful suggestion to strengthen mechanistic validation. To directly test the functional relevance of our findings, we performed comprehensive untargeted lipidomics on microdissected ARC tissue from POMC-AMPKα1 KO and littermate control mice fed HFD. The analysis identified widespread alterations in lipid classes including sphingomyelins and glycerophospholipids **(Figure 8A)**, with prominent reductions in ceramides **(Figure 8A, B; Table EV1).** Short-chain (C16) ceramides were decreased, including Cer 18:2;O2/16:0 **(Figure 8C),** Cer 18:1;O2/16:0 **(Figure 8D),** and Cer 18:0;O2/16:0 **(Figure 8E).** Medium-chain (C18) species such as Cer 18:0;O2/18:0 **(Figure 8F)** and Cer 18:1;O2/18:1/Cer 18:2;O2/18:0 isomers **(Figure 8G, H)** were also reduced; a biochemical change that perfectly mirrored the downregulation in *Cers1* **(Figure 7G)**, the enzyme responsible for C18 ceramide synthesis. Very long-chain ceramides showed marked declines, notably Cer 18:2;O2/22:0 **(Figure 8I),** Cer 18:1;O2/22:0 **(Figure 8J),** and Cer 18:1;O2/25:1 **(Figure 8K).** These data indicate a global suppression of ceramide levels in the MBH upon AMPKα1 deletion in POMC neurons. These detailed lipidomics data unequivocally validate the functional consequences of the phosphoproteomic changes, demonstrating that POMC AMPKα1 deletion leads to reduced hypothalamic ceramide burden under obesogenic conditions. This would establish a mechanistic cascade: AMPKα1 loss → altered phosphosignaling in lipid regulators → lower ARC ceramides → protection against HFD-induced metabolic dysfunction, and highlights ceramide reduction as a novel downstream effector of POMC neuronal AMPKα1 signaling. Thus, the new data strengthen the potential involvement of ceramide metabolism in mediating the effects of AMPKα1 loss on hypothalamic and whole-body metabolic regulation (Contreras *et al*, 2014; Gonzalez-Garcia *et al*, 2018; Hammerschmidt *et al*, 2023; Heras *et al*, 2020; Lopez *et al*, 2025; Martinez-Sanchez *et al*, 2017; Meneyrol *et al*, 2021; Ramírez *et al*, 2013; Turpin-Nolan & Bruning, 2020; Turpin *et al*, 2014). While detailed mechanistic confirmation of individual downstream protein lies beyond the scope of the current study, our findings provide a solid framework and several promising candidates for future functional exploration

Reviewer#1's comment 3: *Autophagic flux is dynamic and might require more sensitive assessments.*

Response: We thank the Reviewer for this important comment regarding the dynamic nature of autophagic flux and the need for sensitive readouts. To address this, we have now quantified p62 levels specifically in POMC neurons from WT and POMC-AMPKα1 KO mice **(Figure EV5H, I)**. These analyses revealed no significant changes in p62, in line with our previous measurements of autophagy-related mRNA expression **(Figure EV5E)** and protein levels **(Figure EV5F, G)**, which were also unchanged between genotypes. Although we acknowledge that more sophisticated flux assays (e.g., in vivo lysosomal blockade or tandem fluorescent LC3 reporters) could, in principle, detect subtle alterations, the convergent lack of differences across transcriptional, protein, and p62 readouts argues against a major contribution of altered autophagy in POMC neurons to the phenotype described.

Reviewer#1's comment 4: *All experiments are done in male mice. Given the known sexual dimorphism in hypothalamic control of energy balance and AMPK signaling, the absence of female mice is a limitation.*

Response: We thank the Reviewer for raising this important point regarding sex specificity in hypothalamic AMPK signaling. In response, we have performed comprehensive phenotypic characterization in female mice across all major experiments, including body weight curves, adiposity, food intake, body and BAT temperatures, BAT UCP1 expression, browning of WAT, glucose/insulin tolerance test, energy expenditure (EE), respiratory exchange ratio (RER) and locomotor activity (LA) **(Figures EV2A-Q**

and EV4A-Q). Our data reveal a striking sexual dimorphism: like males, female POMC-AMPKα1 KO mice exhibit no metabolic alterations on standard chow diet **(Figure EV2A-Q)**. However, in marked contrast to the robust HFD resistance observed in males, HFD-fed females showed no major differences in body weight gain, fat mass accumulation, or metabolic phenotyping parameters compared to controls **(Figure EV4A-Q).** This sex-specific HFD response indicates that AMPKα1 in POMC neurons exerts diet-dependent, sexually dimorphic control over energy balance, likely reflecting gonadal hormone modulation of hypothalamic nutrient sensing. We thank the Reviewer for highlighting this critical aspect, which has revealed an important new conclusion regarding sex differences in POMC neuronal AMPKα1 function.

Reviewer#1's comment 5: *The conclusion that POMC-AMKPα1 KO increases BAT thermogenesis via enhanced SNS outflow is indirect.*

Response: We thank the Reviewer for pointing out the need for more direct evidence of SNS activation. To address this, we have now measured noradrenaline (NE) levels in BAT tissue by HPLC in POMC-AMPKα1 KO vs. control mice from both chow-fed and HFD cohorts. These data revealed a significant elevation of NE in the null mice, specifically under HFD conditions, **(Figure 3I),** in full consonance with the diet-dependent BAT phenotype, while no differences were observed on chow (**Figure 2I**). This provides direct biochemical confirmation of increased sympathetic outflow to BAT in the relevant metabolic challenge context, strengthening the mechanistic link to enhanced thermogenesis.

Reviewer#1's comment 6: *AMPKα2 compensates functionally in KO mice, can be shown in the supplementary figure.*

Response: We thank the Reviewer for highlighting the potential for AMPKα2 compensation in POMC-AMPKα1 KO mice. To directly assess this, we performed western blots on ARC lysates from KO and control mice, quantifying total AMPKα2 protein levels **(Figure EV5A-D).** These analyses revealed no upregulation of AMPKα2 in KO tissue, indicating a lack of compensatory adaptation at the protein level. This absence of compensation supports the subunit-specific phosphoproteomic and phenotypic differences observed, underscoring lack of functional compensation for AMPKα1 vs. AMPKα2 in POMC neuronal metabolic regulation.

Reviewer#1's comment 7: *Supplementary figures for adipocyte area, UCP1 staining, and autophagy markers, but not fully referenced in the main text.*

Response: We thank the Reviewer for this observation. These results were indeed cited in the previous version of the manuscript. To further address this concern and enhance clarity, we have now explained this in a better way, referencing **Figure EV1A-D** and **Figure 3A-D**. We apologize for the lack of clarity in the former version.

Reviewer#1's comment 8: *Include effect sizes and sample sizes in figure legends.*

Response: We thank the Reviewer for this suggestion. Sample sizes have been added to all relevant Figure Legends. Effect sizes were calculated as Cohen's d (please, see Material and Methods). The complete statistical information is included in the Source Data File.

We sincerely thank the Reviewer#1 for the outstanding and insightful comments, which have greatly contributed to improving the clarity and rigor of our manuscript.

REVIEWER#2

Overall comment: *In this manuscript, the authors investigated the metabolic consequences of deleting AMPKa1 in POMC neurons. While this results in no apparent phenotype on a standard chow diet, upon high fat feeding, knockout animals show reduced weight gain. This appears to be driven by increased energy expenditure, as food intake is unchanged. The authors argue that this phenotype is the result of increased BAT thermogenesis, due to small but statistically significant increases in BAT temperature and UCP1 protein. They then go on to investigate molecular mechanisms and show that AMPKa1 deletion in POMC neurons leads to rewiring of the phosphoproteome in the arcuate nucleus. Moreover, knockout animals show a more beneficial ER stress response and decreased expression of ceramide biosynthesis transcripts. Overall, this is an interesting study that adds to the body of knowledge from this group and others on the role of hypothalamic AMPK signaling in coordinating whole-body metabolism. However, additional experiments are needed to fully support the claim that the phenotype measured here is due to BAT activation.*

Response: We sincerely thank Reviewer #2 for the outstanding and insightful comments, which have significantly helped us to improve the clarity, robustness, and overall quality of the manuscript. Please note that throughout this Rebuttal, **all Figure numbering refers to the new version of the manuscript**

Reviewer#2's comment 1: *To more fully substantiate the argument that knockout mice have increased BAT activity, a more complete characterization of the brown fat is needed. This should include mRNA analysis of Ucp1 and other thermogenic genes in chow and HFD (Pgc1a, Dio2). Additionally, to help clarify whether this apparent increase in BAT activity is due to diet-induced thermogenesis or a broader increase in BAT activity, the authors should do cold-tolerance tests in control and knockout animals. Body temperature and BAT temperature should be measured before and after a cold challenge, and upon sacrifice, thermogenic gene mRNAs should be measured.*

Response: We thank the Reviewer for these excellent suggestions that prompted important mechanistic clarification. Following their guidance:

1. **We performed comprehensive qPCR analysis of canonical thermogenic genes** (*Ucp1, Dio2, Ppargc1a, Prdm16 and Pparg*) in the BAT from POMC-AMPKα1 KO vs. control mice under both chow and HFD conditions **(Rebuttal Figure R1A, B)**. These results revealed no major changes between genotypes in those transcripts. Not even in in POMC-AMPKα1 KO fed a HFD, consistent with chronic SNS-driven BAT activation where protein-level adaptations (e.g., UCP1 accumulation by Western blot; **Figure 3G, H)** typically predominate over mRNA shifts (Nedergaard & Cannon, 2013). In alignment with the literature, our conclusions are based on the UCP1 protein levels as accepted proxy for the metabolic significance (Nedergaard & Cannon, 2013).

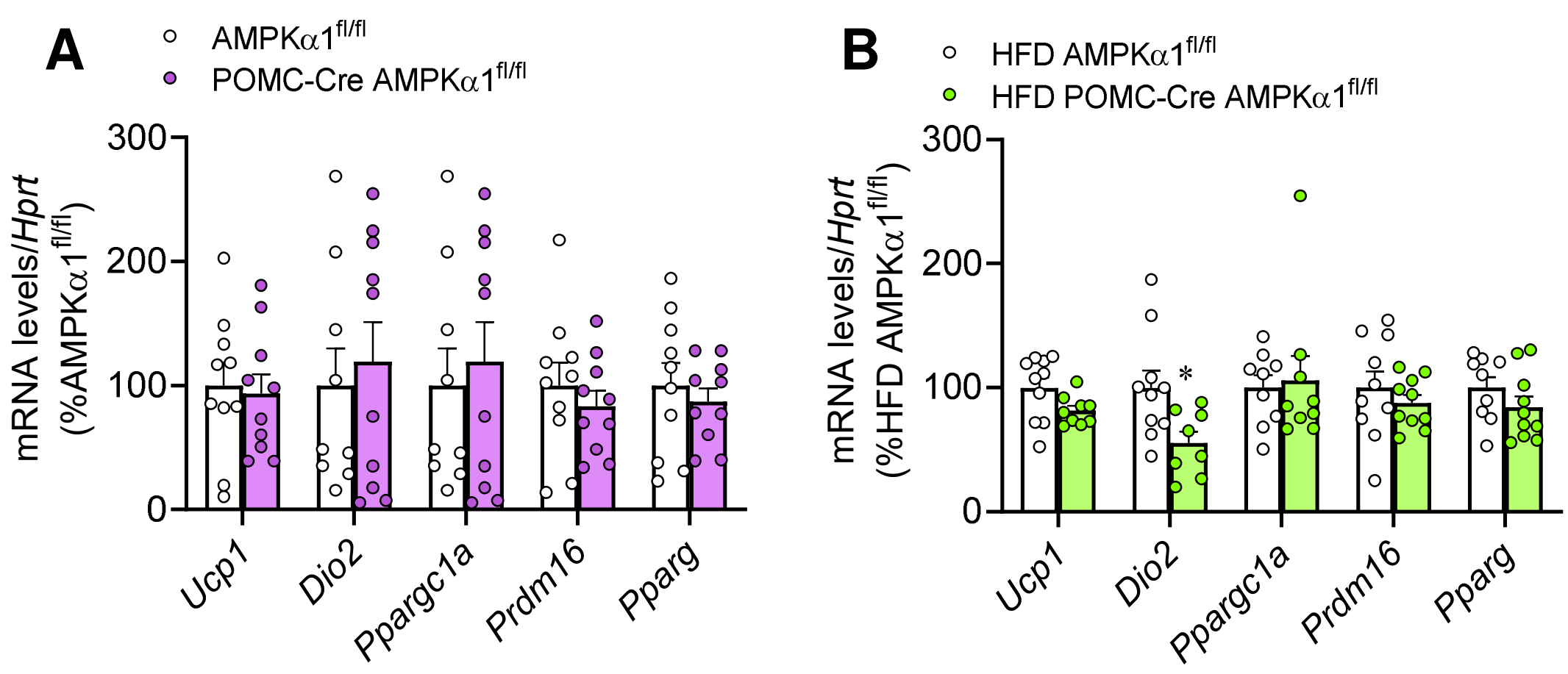

Figure R1. Effect of loss of AMPKα1 in POMC neurons on BAT thermogenic expression. mRNA levels of genes expressing proteins involved in thermogenesis in control (littermates AMPKα1$^{fl/fl}$) and POMC-Cre AMPKα1$^{fl/fl}$ male mice fed a **(A)** normal chow and **(B)** HFD. Data expressed as mean±SEM. Statistical significance was assessed by Student's t-test. *P<0.05, **P<0.01 vs. AMPKα1$^{fl/fl}$.

2. **To discriminate cold-induced vs. basal thermogenesis, we conducted acute cold-tolerance tests** (4°C for 6h) measuring core body temperature, intrascapular BAT temperature, and tail temperature in both dietary contexts (chow: **Figure 4A-E**; HFD: **Figure 4F-J**). Remarkably, no genotype differences emerged in body temperature defense capacity, BAT temperature kinetics, or tail temperature, indicating preserved cold-adaptive responses.
3. **We further examined metabolic adaptation to HFD transition** through detailed indirect calorimetry over a full 48-hour dietary switch (chow to HFD), capturing the dynamic period of diet acclimation when diet-induced thermogenesis is most prominent **(Figure 4K-M).** This analysis tracked energy expenditure (EE), oxygen consumption (VO2), and respiratory exchange ratio (RER) across light/dark cycles. No genotype differences were observed: **i)** VO_2 and EE remained equivalent despite KO resistance to HFD weight gain, and **ii)** RER declined similarly in both groups, reflecting comparable fat oxidation shifts. This comprehensive profiling definitively rules out compensatory diet-induced-thermogenesis or altered dietary fuel utilization as explanations for the lean KO phenotype.

Together, these negative results provide strong positive evidence: the BAT phenotype reflects elevated basal SNS outflow (corroborated by direct noradrenaline measurement, **Reviewer #1 Comment 5; Figures 2I and 3I)** rather than enhanced adaptive thermogenic capacity. This distinction refines our model, positioning POMC AMPKα1 as a specific negative regulator of constitutive BAT tone rather than stress-responsive thermogenesis, with important translational implications for therapeutic targeting of basal energy expenditure (Milbank *et al*, 2023; Milbank *et al*, 2021; Mukherjee *et al*, 2023). Notably, this uncouples POMC AMPKα1 from the dual basal/cold-adaptive role well-established for SF1 neuronal AMPKα1 (Martinez-Sanchez *et al.*, 2017; Seoane-Collazo *et al*, 2018), highlighting regional, nucleus-specific, subunit specificity. We thank the reviewer for these insightful comments that strengthened the study.

Reviewer#2's comment 2: *In Figure 4, it is clear that the knockout mice gain significantly less weight on high fat diet. However, in Figure 4B, there is no difference in the weights of the major fat depots. This raises the possibility that the knockouts actually have decreased muscle mass. This needs to be addressed with body composition analysis.*

Response: We thank the Reviewer for noting this apparent discrepancy and suggesting body composition analysis. We must recognize that he/she is totally right on this point. In fact, we agree that weighing the fat pads is not the best method for the analysis of adiposity. In any case, the summatory of both sWAT and gWAT shows a clear tendency for adiposity to be decreased: HFD AMPKα1$^{fl/fl}$: 2.70±0.27 g; HFD POMC-Cre AMPKα1$^{fl/fl}$: 2.13±1.18 g; P=0.0696), in keeping with the weight loss. In any case, to address potential lean mass changes, we performed nuclear magnetic analysis (NMR) on HFD-fed POMC-AMPKα1 KO and control mice, revealing no differences in lean body mass between genotypes **(Rebuttal Figure R2)**.

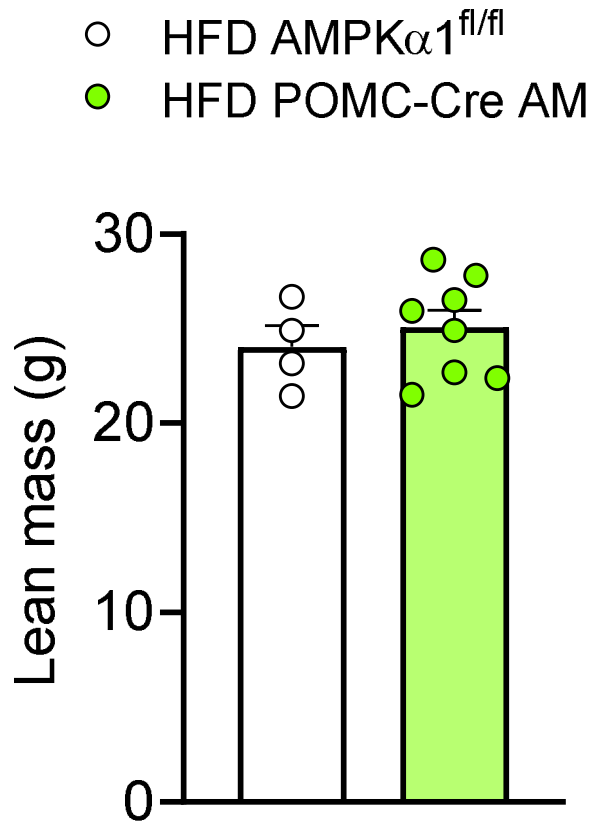

Figure R2. Effect of loss of AMPKα1 in POMC neurons on lean mass. NRM analysis of control (littermates AMPKα1$^{fl/fl}$) and POMC-Cre AMPKα1$^{fl/fl}$ male mice fed a HFD. Data expressed as mean±SEM. Statistical significance was assessed by Student's t-test.

Reviewer#2's comment 3: *From the methods, it appears that all the studies were done in male mice. The authors should either extend their studies to female mice, or they need to state explicitly in the text that they only studied male mice and thus do not know if the same findings hold in females.*

Response: We thank the Reviewer for raising this important point regarding sex specificity in hypothalamic AMPK signaling. In response, we have performed comprehensive phenotypic characterization in female mice across all major experiments, including body weight curves, food intake, body composition, BAT UCP1 expression and activity, glucose/insulin tolerance, core body and BAT temperature and indirect calorimetry **(Figures EV2A-Q and EV3A-Q).** Our data reveal a striking sexual dimorphism: like males, female POMC-AMPKα1 KO mice exhibit no metabolic alterations on standard chow diet **(Figure EV2A-Q)**. However, in marked contrast to the robust HFD resistance observed in males, HFD-fed females showed no major differences in body weight gain, fat mass accumulation, or metabolic phenotyping parameters compared to controls **(Figure EV3A-Q).** This sex-specific HFD response indicates that AMPKα1 in POMC neurons exerts diet-dependent, sexually dimorphic control over energy balance, likely reflecting gonadal hormone modulation of hypothalamic nutrient sensing. We thank the Reviewer for highlighting this critical aspect, which has revealed an important new conclusion regarding sex differences in POMC neuronal AMPKα1 function.

Reviewer#2's comment 4: *For the metabolic characterization (GTT, ITT, indirect calorimetry), the authors need to state explicitly what time points they were performed.*

Response: We thank the Reviewer for requesting this important clarification to ensure full reproducibility. We have now explicitly detailed all time points in the revised Methods section:

- **GTT:** intraperitoneal glucose injection following 12h overnight fasting, with blood glucose measured at baseline (0 min) and post-injection time points 15, 30, 60, 90, and 120 min.
- **ITT:** intraperitoneal insulin injection following 6h fasting, with blood glucose measured at baseline (0 min) and post-injection time points 10/15, 20, 30, 60, and 90 min.
- **Indirect calorimetry:** continuous 48-hour monitoring in **Figure 2O-R, 3O-R, EV2N-Q and EV4N-Q** or 120-hour monitoring in **Figure 4K-M** of O_2 consumption (VO_2), energy expenditure (EE), respiratory exchange ratio (RER) and locomotor activity (LA) were recorded every 30 minutes across light/dark cycles.

These precise timelines have also been accordingly explained in the Material and Methods section.

Reviewer#2's comment 5: *In Figure 8, the argument about ceramides would be more compelling if they actually measured ceramide levels.*

Response: We thank the Reviewer for this valuable suggestion to validate the phosphoproteomic findings functionally. In response, we performed comprehensive untargeted lipidomics on microdissected mediobasal hypothalamus (MBH, containing ARC) tissue from POMC-AMPKα1 KO and littermate control mice fed HFD. The analysis identified widespread alterations in lipid classes including sphingomyelins and glycerophospholipids **(Figure 8A)**, with prominent reductions in ceramides **(Figure 8A, B; Table EV1).** Short-chain (C16) ceramides were decreased, including Cer 18:2;O2/16:0 **(Figure 8C),** Cer 18:1;O2/16:0 **(Figure 8D),** and Cer 18:0;O2/16:0 **(Figure 8E).** Medium-chain (C18) species such as Cer 18:0;O2/18:0 **(Figure 8F)** and Cer 18:1;O2/18:1/Cer 18:2;O2/18:0 isomers **(Figure 8G, H)** were also reduced; a biochemical change that perfectly mirrored the downregulation in *Cers1* **(Figure 7G)**, the

enzyme responsible for C18 ceramide synthesis. Very long-chain ceramides showed marked declines, notably Cer 18:2;O2/22:0 **(Figure 8I),** Cer 18:1;O2/22:0 **(Figure 8J),** and Cer 18:1;O2/25:1 **(Figure 8K).** These data indicate a global suppression of ceramide levels in the MBH upon AMPKα1 deletion in POMC neurons. These detailed lipidomics data unequivocally validate the functional consequences of the phosphoproteomic changes, demonstrating that POMC AMPKα1 deletion leads to reduced hypothalamic ceramide burden under obesogenic conditions. This would establish a mechanistic cascade: AMPKα1 loss → altered phosphosignaling in lipid regulators → lower ARC ceramides → protection against HFD-induced metabolic dysfunction, and highlights ceramide reduction as a novel downstream effector of POMC neuronal AMPKα1 signaling. Thus, the new data strengthen the potential involvement of ceramide metabolism in mediating the effects of AMPKα1 loss on hypothalamic and whole-body metabolic regulation (Contreras *et al.*, 2014; Gonzalez-Garcia *et al.*, 2018; Hammerschmidt *et al.*, 2023; Heras *et al.*, 2020; Lopez *et al.*, 2025; Martinez-Sanchez *et al.*, 2017; Meneyrol *et al.*, 2021; Ramírez *et al.*, 2013; Turpin-Nolan & Bruning, 2020; Turpin *et al.*, 2014).

Reviewer#2's comment 6: *The discussion is very long. It would be more readable if it was shortened.*

Response: We thank the Reviewer for this feedback on readability. We have shortened the Discussion by 30% (from approx. 1750 to approx. 1250 words, despite incorporating new data suggested by the Editor and Reviewers), condensing redundant mechanistic interpretations, consolidating subunit comparisons and focusing on key novel findings The revised version is now more concise and reader friendly.

We sincerely thank the Reviewer#2 for the outstanding and insightful comments, which have greatly contributed to improving the clarity and rigor of our manuscript.

REFERENCES

Contreras C, González-García I, Martínez-Sánchez N, Seoane-Collazo P, Jacas J, Morgan DA, Serra D, Gallego R, González F, Casals N *et al* (2014) Central Ceramide-Induced Hypothalamic Lipotoxicity and ER Stress Regulate Energy Balance. *Cell Rep* 9: 366-377

Gonzalez-Garcia I, Contreras C, Estevez-Salguero A, Ruiz-Pino F, Colsh B, Pensado I, Linares-Pose L, Rial-Pensado E, Martinez de Morentin PB, Ferno J *et al* (2018) Estradiol Regulates Energy Balance by Ameliorating Hypothalamic Ceramide-Induced ER Stress. *Cell Rep* 25: 413-423

Hammerschmidt P, Steculorum SM, Bandet CL, Del Rio-Martin A, Steuernagel L, Kohlhaas V, Feldmann M, Varela L, Majcher A, Quatorze Correia M *et al* (2023) CerS6-dependent ceramide synthesis in hypothalamic neurons promotes ER/mitochondrial stress and impairs glucose homeostasis in obese mice. *Nat Commun* 14: 7824

Heras V, Castellano JM, Fernandois D, Velasco I, Rodriguez-Vazquez E, Roa J, Vazquez MJ, Ruiz-Pino F, Rubio M, Pineda R *et al* (2020) Central Ceramide Signaling Mediates Obesity-Induced Precocious Puberty. *Cell Metab* 32: 951-966 e958

Lopez M, Dieguez C, Tena-Sempere M, Gonzalez-Garcia I (2025) Ceramides in the central control of metabolism. *Trends Endocrinol Metab* 36: 11-14

Martinez-Sanchez N, Seoane-Collazo P, Contreras C, Varela L, Villarroya J, Rial-Pensado E, Buque X, Aurrekoetxea I, Delgado TC, Vazquez-Martinez R *et al* (2017) Hypothalamic AMPK-ER Stress-JNK1 Axis Mediates the Central Actions of Thyroid Hormones on Energy Balance. *Cell Metab* 26: 212-229 e212

Meneyrol K, Estevez-Salguero A, Gonzalez-Garcia I, Guitton J, Taouis M, Benomar Y, Magnan C, Lopez M, Le Stunff H (2021) Ovarian insufficiency impairs glucose-stimulated insulin secretion through activation of hypothalamic de novo ceramide synthesis. *Metabolism* 123: 154846

Milbank E, Dragano N, Vidal-Gomez X, Rivas-Limeres V, Garrido-Gil P, Wertheimer M, Recoquillon S, Pata MP, Labandeira-Garcia JL, Dieguez C *et al* (2023) Small extracellular vesicle targeting of hypothalamic AMPKalpha1 promotes weight loss in leptin receptor deficient mice. *Metabolism* 139: 155350

Milbank E, Dragano NRV, Gonzalez-Garcia I, Garcia MR, Rivas-Limeres V, Perdomo L, Hilairet G, Ruiz-Pino F, Mallegol P, Morgan DA *et al* (2021) Small extracellular vesicle-mediated targeting of hypothalamic AMPKalpha1 corrects obesity through BAT activation. *Nat Metab* 3: 1415-1431

Mukherjee S, Dieguez C, Ferno J, Lopez M (2023) Obesity wars: hypothalamic sEVs a new hope. *Trends Mol Med* 29: 622-634

Nedergaard J, Cannon B (2013) UCP1 mRNA does not produce heat. *Biochim Biophys Acta* 1831: 943-949

Ramírez S, Martíns L, Jacas J, Carrasco P, Pozo M, Clotet J, Serra D, Hegardt FG, Diéguez C, López M *et al* (2013) Hypothalamic ceramide levels regulated by CPT1C mediate the orexigenic effect of ghrelin. *Diabetes* 62: 2329-2337

Seoane-Collazo P, Roa J, Rial-Pensado E, Linares-Pose L, Beiroa D, Ruiz-Pino F, Lopez-Gonzalez T, Morgan DA, Pardavila JA, Sanchez-Tapia MJ *et al* (2018) SF1-Specific AMPKalpha1 Deletion Protects Against Diet-Induced Obesity. *Diabetes* 67: 2213-2226

Turpin-Nolan SM, Bruning JC (2020) The role of ceramides in metabolic disorders: when size and localization matters. *Nat Rev Endocrinol* 16: 224-233

Turpin SM, Nicholls HT, Willmes DM, Mourier A, Brodesser S, Wunderlich CM, Mauer J, Xu E, Hammerschmidt P, Bronneke HS *et al* (2014) Obesity-induced CerS6-dependent C16:0 ceramide production promotes weight gain and glucose intolerance. *Cell Metab* 20: 678-686

EDITOR

Overall comment: *Thank you for considering EMBO Reports. We appreciate the conceptual advance of your study, the amount of new data added in revision, and the arguments presented in your appeal. We would invite a final revision of your paper in line with the appeal you sent to Daniel on June 7th.*

Response: We sincerely thank the Editor for the positive evaluation of our study, for recognizing the conceptual advance of the work, and for appreciating the substantial amount of new data added in revision, as well as the arguments presented in our Appeal. We are very grateful for the opportunity to submit a final revision to EMBO Reports and will revise the manuscript accordingly, in line with the guidance provided in our appeal of June 7th.

REVIEWER#2

Overall comment: *In the revised manuscript, the authors have added extensive phenotyping of female POMC Cre AMPKa1 knockouts. The female mice do not have any evident phenotype on a standard or high fat diet. The authors also performed lipidomics experiments and found decreased levels of ceramides in knockout mice. While the authors have made efforts to address the points raised in my initial review, significant concerns remain.*

Response: We thank the Reviewer for acknowledging the substantial additional work performed in the revised manuscript, including the extensive phenotyping of female POMC-Cre AMPKα1 knockout mice and the lipidomics analyses showing decreased ceramide levels in knockout mice. We appreciate the Reviewer's careful evaluation and the opportunity to further clarify our study.

Reviewer#2's comment 1: *The female data in EV4 shows no significant difference in body weight. However, the weight curves have started to diverge, raising the distinct possibility that if followed longer, the female mice may in fact be protected from dietary obesity, just as the males are. This should be clarified.*

Response: We thank the Reviewer for this important comment. We agree that the body weight curves in female mice show a modest tendency to diverge at the later time point, but this difference does not reach statistical significance and is much less pronounced than in males, where the phenotype emerges earlier and is clearly sustained over time. More importantly, despite this slight late trend in body weight, female mice do not display the accompanying metabolic changes observed in males, including alterations in BAT thermogenesis, energy expenditure, or the broader obesity-resistant phenotype. For this reason, we believe the current data support a sex-dependent effect rather than a robust protective phenotype in females.

Reviewer#2's comment 2: *A greater concern is that the female phenotyping appears to have been done at a later date than the male phenotyping, making it difficult to know whether the reported sexual dimorphism (if it even exists, see point 1 above) truly represents a sex effect as opposed to a batch effect.*

Response: Response: We thank the Reviewer for raising this important point. We agree that sex comparisons are best interpreted when male and female data are obtained under matched experimental conditions. In our case, all experiments were performed under the same controlled housing, diet, and phenotyping protocols, and a subset of the female measurements was collected in parallel with the male cohort, yielding consistent results. Therefore, we do not consider batch effects to be a plausible explanation for the observed sex-dependent differences. This has now been explicitly stated in the revised manuscript.

Reviewer#2's comment 3: *The male phenotyping data remains very difficult to interpret. First, male knockout mice gain significantly less weight on high fat diet, yet they show no difference in fat mass or in lean mass. What then accounts for the difference in body weight? Second, the authors state that males have increased energy expenditure, but the difference between controls and knockouts in Fig 3P has a P value of 0.1588. So, either the study is underpowered or there is no statistically significant difference in energy expenditure.*

Response: We thank the Reviewer for this careful analysis. Regarding the discrepancy between body weight and fat/lean mass measurements, we agree that these individual parameters can appear difficult to reconcile when considered in isolation. However, body weight is the integrated outcome of multiple components, including cumulative differences in energy balance, tissue composition dynamics, and potentially subtle changes in water or organ mass that may not be fully captured by terminal fat and lean mass measurements. In our study, the overall phenotype is best interpreted from the full longitudinal dataset rather than from any single endpoint alone. Concerning energy expenditure, **Figure 3P** corresponds to an ANCOVA model adjusted for lean mass, and in the current dataset the adjusted group effect does not reach conventional statistical significance (P=0.1588). We therefore interpret this result cautiously. At the same time, the circadian ANOVA in **Figure 3O** suggests a trend toward a group-related difference in the temporal pattern of energy expenditure (P=0.08), which is consistent with the longitudinal reduction in body weight gain, the associated thermogenic readouts, and the broader metabolic profile observed across the study. Taken together, these analyses support a trend toward altered energy expenditure in the knockout mice. This has now been explicitly stated in the revised manuscript.

Reviewer#2's comment 4: *The authors claim that their indirect calorimetry data demonstrates increased sympathetic outflow to BAT, but this point is not supported by any experimental data. In order to argue that there is increased sympathetic outflow, sympathetic nerve recordings would be needed. Additionally, the authors claim that the purported difference in energy expenditure (even though there is no statistically significant difference) is BAT-specific, but this too is not supported by data. In order to make this claim, the authors would need to do tissue respiration from BAT and other tissues to show BAT specificity and/or do indirect calorimetry in HFD fed animals at thermoneutrality to see if the difference between genotypes disappears.*

Response: We thank the Reviewer for this comment. We respectfully note that **Figure 3I** shows increased noradrenaline (NE) levels in BAT of knockout mice, which is a widely accepted indirect marker of sympathetic activity in this tissue. While we agree that direct sympathetic nerve recordings would provide more definitive evidence, we believe these data, together with the thermogenic phenotype, are consistent with increased sympathetic activation of BAT in the knockout animals. In relation to the thermoneutrality, we do not fully understand the rationale behind the request for it: by definition, BAT is switched off under these conditions, making this approach unsuitable for assessing BAT thermogenesis in the context of our study.

Reviewer#2's comment 5: *The authors never state whether the phosphoproteomics were done on fed or fasted animals, which could of course affect the findings.*

Response: We thank the Reviewer for raising this point. The phosphoproteomics experiments were performed in fed animals, and we have clarified this in the revised manuscript. We agree that feeding status is an important experimental variable, and we now state it explicitly to avoid any ambiguity.

Reviewer#2's comment 6: *Although not absolutely required, it would have been nice if the authors either did the phosphoproteomics and lipidomics analyses in females or at least measured key phosphoproteins and ceramides in females. If the female knockout mice do not show the differences seen in males, this would strengthen the contention of a sex-specific difference.*

Response: We thank the Reviewer for this suggestion. However, given the (unfounded) concerns raised regarding possible batch effects in the female experiments, we do not understand the Reviewer's point about the additional female phosphoproteomic or lipidomic analyses to be essential for the current revision. The extensive female phenotyping already included in the manuscript shows no overt metabolic phenotype under either standard or high-fat diet conditions, which is sufficient to support the sex-dependent nature of the effect.

ADDITIONAL EXPERT ADVISOR

Overall comment: *This is a revised version and I was asked to assess this study as referee advisor in the second round. Overall, it seems to me that the revision made the paper much stronger but there are still a few things that remain questionable. Obviously, I would have asked them in a first round already so please accept my apologies for any criticism, as it is meant in a constructive way. The paper is altogether well prepared but still has some weaknesses as explained below.*

Response: We thank the Additional Expert Advisor for the careful evaluation of the revised manuscript and for recognizing that the revision has made the paper substantially stronger. We also appreciate the constructive nature of the comments and understand that, as a second-round assessment, some points may have been raised now that could reasonably have been addressed earlier.

Additional expert advisor's comment 1: *This is a new study, new data, but AMPK in POMC neurons is not a new thing, which limits the novelty to a certain extent.*

Response: We respectfully disagree with the assessment that the novelty is limited. A PubMed search yields no studies specifically addressing the role of AMPKα1 in POMC neurons, and we have included the corresponding screenshot for reference.

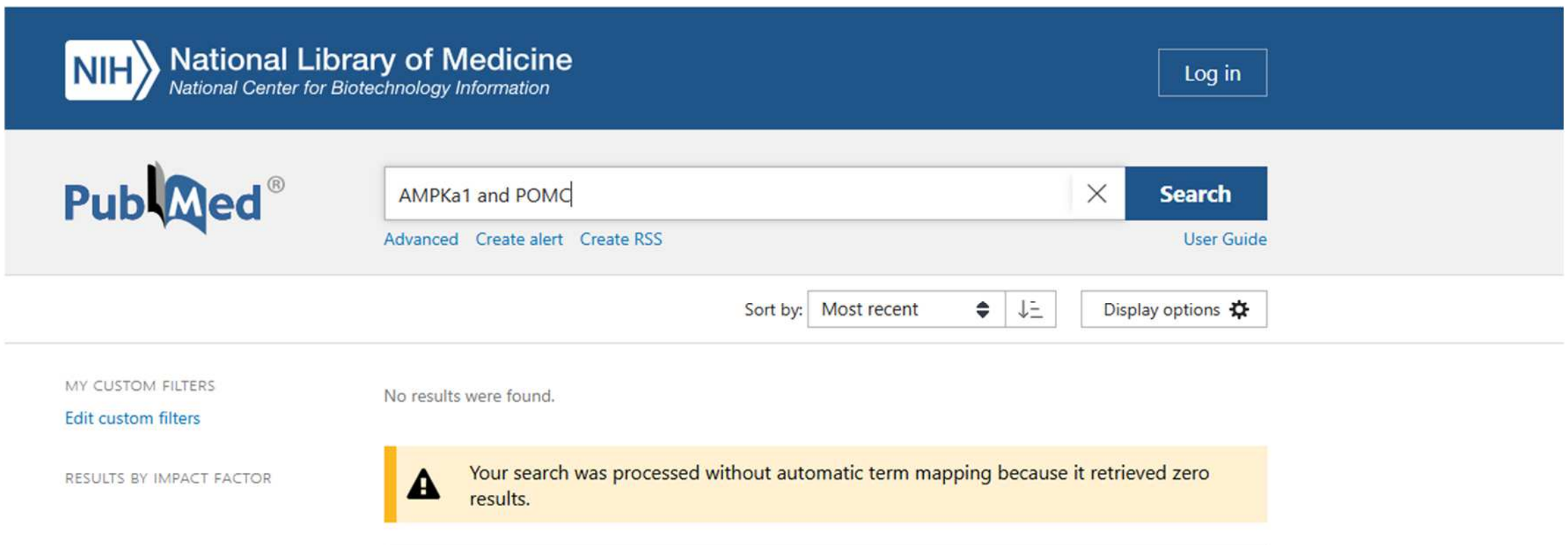

While AMPK signaling in POMC neurons has been examined in broader terms, the present work defines an isoform-specific function for AMPKα1 and identifies a previously unrecognized, sex-dependent role in the regulation of energy balance and BAT thermogenesis

Additional expert advisor's comment 2: *Pursuant to that, I don't think we now really learnt what is going on these mice and many things remain unclear: Why are there sex differences, where does the difference in weight come from? Only the males show this difference in body weight but the fat pads and lean mass are the same - confusing! So, what is it?*

Response: We thank the Additional Expert Advisor for this comment. We agree that the relationship between body weight, fat mass, and lean mass requires careful interpretation. In our study, the male phenotype is characterized by a clear reduction in body weight gain under HFD, whereas the terminal body composition data do not show large differences in fat or lean mass at the specific endpoint analyzed. We believe this apparent discrepancy reflects the fact that body weight integrates cumulative changes over time, whereas terminal composition measures capture only a single time point. In addition, the metabolic phenotype is supported by the longitudinal body weight curves and the associated thermogenic readouts, rather than by body composition alone. We have revised the text to clarify this point and to avoid overinterpreting any single parameter.

Additional expert advisor's comment 3: *Is it really BAT thermogenesis? I would have asked different experiments (Measuring NST by indirect calorimetry, thermoneutrality, yet the data are mostly robust and done well (even though the energy expenditure is neither different in figure 2 nor 3, insignificant!).*

Additional expert advisor's comment 4: *In the end, the authors claim that there is no phenotype in chow mice but then when you feed HFD you get one - nice, but they explicitly show that HFD doesn't change energy expenditure (Fig 4 k-m) - how does that add up?*

Response: We thank the Additional Expert Advisor for these comments. Indirect calorimetry and non-shivering thermogenesis were extensively assessed in the revised manuscript across multiple figures and experimental conditions **(Figures 2O–R, 3O–R, 4K–L, EV2N–Q, and EV3N–Q)**, and we have now revised the text to make these analyses easier to follow and to avoid any ambiguity in their interpretation. Regarding thermoneutrality, we respectfully note that this condition is not appropriate for evaluating BAT thermogenesis in the context of our study, because BAT is functionally inactive under thermoneutral conditions. For this reason, our conclusions are based on the integrated physiological, biochemical, and thermogenic readouts already presented in the manuscript.

Dear Prof. Lopez

Thank you for the submission of your revised manuscript to EMBO reports. We are inviting you to submit a final revision in line with the revision plan you shared with Daniel Klimmeck.

From the editorial side, there are also a few things that we need before we can proceed with the official acceptance of your study:

- As a standard procedure, we edit the abstract of manuscripts to make them more accessible to a general readership. Please find the suggested version below my signature.

- Please rename the Materials and Methods section to Methods.

- Please rename the COMPETING INTEREST section to Disclosure and Competing Interests Statement.

- Please only use DOI for preprints or datasets that have not yet been published.

- CRediT has replaced the traditional author contributions section because it offers a systematic machine readable author contributions format that allows for more effective research assessment. Please remove the Author Contributions section from the manuscript and use the free text boxes beneath each contributing author's name in our online systems to add specific details on the author's contribution. More information is available in our guide to authors.

- Please ensure that all funding is submitted in the system, including RN: ERC Synergy Grant-2019-WATCH-810331; ERC Consolidator Grant 101124230-Ghostbuster, IGG: ERC Starting Grant 101219072-HypoPause, the Xunta de Galicia (2020-2023, ED431G/05), Maria de Maetzu Excellence Accreditation (CEX2024-001463-M), Instituto de Salud Carlos III... funded by MICIN/AEI/10.13039/501100011033.

- Please adjust the in-text callouts for individual figures and figure panels: e.g. 5F appears to be missing.

- As Tabe EV1 has two sheets in the Excel file, please update it to Dataset EV1 in all places.

- Please note that EMBO press papers are accompanied online by:
A) a short (2 sentences) summary of the findings and their significance,
B) 2-5 short bullet points highlighting the key results, and
C) a synopsis image in .jpg or .png format that is exactly 550 pixels wide and 300 pixels high. Please note that the text needs to be legible at the final size. Please upload this information along with your revised manuscript (the text for A and B should be provided in one separate Word file uploaded as Cover Art/Synopsis Image).

- The resolution of the submitted figures is too low for blot and microscopy images. Although there is a decent file size for all files, this reduction in resolution is commonly caused by converting original 16-bit TIFF files to RGB format for publication. While this is not inherently problematic, it can raise concerns about image integrity for critical readers.
To avoid any misunderstanding and to meet EMBO Press standards, we kindly ask that you:
* Resubmit the complete figure set at the captured original data resolution.

- The Methods section "HPLC-based neurotransmitter quantification" displays a high similarity to published work. Please include a citation.

- Our production/data editors have asked you to clarify several points in the figure legends (see below). Please incorporate these changes in the manuscript and return the revised file with tracked changes with your final manuscript submission.
- Please note that the exact p values are not provided in the legends of figures 7D, F; 8F, G, H, I, J, K
- Please indicate the statistical test used for data analysis in the legends of figures 5C, D
- Please note that the white arrow heads are not defined in the legend of figure EV5 H. This needs to be rectified.

We look forward to seeing a final version of your manuscript as soon as possible.

Kurt Weir
Editor
EMBO Reports

Proopiomelanocortin (POMC) neurons in the arcuate nucleus of the hypothalamus (ARC) play a critical role in energy homeostasis. 5'-adenosine monophosphate-activated protein kinase (AMPK) is a serine/threonine kinase, which acts as the main energy sensor in the cell. The heterotrimeric AMPK results from the combination of a catalytic α subunit (α1, α2) with two

regulatory subunits, β (β1, β2) and γ (γ1, γ2 and γ3). Hypothalamic AMPK plays a key role in the control of energy balance, but current evidence shows that the precise combination of its heterotrimeric components determines its physiological action. Our findings show that AMPKα1 ablation in POMC neurons has a sex-dependent protective effect against diet-induced obesity because of increased thermogenesis in brown adipose tissue (BAT) in males, but not in females. At the molecular level, deletion of AMPKα1 in POMC neurons disrupts the ARC phosphoproteome and is associated with ameliorated endoplasmic reticulum (ER) stress and reduced ceramide content. These findings highlight the complex function of the different AMPK subunits in the hypothalamic regulation of energy balance.

Rebuttal EMBOR-2025-62113V2

EDITOR

Overall comment: *Thank you for the submission of your revised manuscript to EMBO reports. We are inviting you to submit a final revision in line with the revision plan you shared with Daniel Klimmeck. From the editorial side, there are also a few things that we need before we can proceed with the official acceptance of your study:*

Response: We thank the Editor for the positive evaluation of our study and for recognizing the conceptual advance and the substantial new data added in the revised manuscript, as well as the points raised in our Appeal. We are grateful for the opportunity to submit a final revision to EMBO Reports and have revised the manuscript accordingly in line with the comments received.

Editor comment 1: *As a standard procedure, we edit the abstract of manuscripts to make them more accessible to a general readership. Please find the suggested version below my signature.*

Response: We thank the Editor for the suggested revision of the abstract aimed at improving accessibility for a broader readership. We fully agree with these changes and have adopted the edited abstract as proposed in the new version.

Editor comment 2: *Please rename the Materials and Methods section to Methods.*

Response: We have renamed the "Materials and Methods" section to "Methods" as requested.

Editor comment 3: *Please rename the COMPETING INTEREST section to Disclosure and Competing Interests Statement.*

Response: We have renamed the "Competing Interest" section to "Disclosure and Competing Interests Statement" as requested

Editor comment 4: *Please only use DOI for preprints or datasets that have not yet been published.*

Response: We have revised the references so that DOIs are only used for unpublished preprints and datasets, as requested.

Editor comment 5: *CRediT has replaced the traditional author contributions section because it offers a systematic machine readable author contributions format that allows for more effective research assessment. Please remove the Author Contributions section from the manuscript and use the free text boxes beneath each contributing author's name in our online systems to add specific details on the author's contribution. More information is available in our guide to authors.*

Response: We have removed the "Author Contributions" section from the manuscript and have provided the required CRediT information for each author in the online submission system, as requested.

Editor comment 6: *Please ensure that all funding is submitted in the system, including RN: ERC Synergy Grant-2019-WATCH-810331; ERC Consolidator Grant 101124230-Ghostbuster, IGG: ERC Starting Grant 101219072-HypoPause, the Xunta de Galicia (2020-2023, ED431G/05), Maria de Maetzu Excellence Accreditation (CEX2024-001463-M), Instituto de Salud Carlos III... funded by MICIN/AEI/10.13039/501100011033.*

Response: We have verified and updated the funding information so that all listed grants and agencies, including those indicated by the Editor, are correctly entered in the online system and in the manuscript, as requested.

Editor comment 7: *Please adjust the in-text callouts for individual figures and figure panels: e.g. 5F appears to be missing.*

Response: We have carefully checked all in-text figure callouts and have corrected and completed the references to individual figures and panels, including the missing callout to Figure 5F.

Editor comment 8: *As Tabe EV1 has two sheets in the Excel file, please update it to Dataset EV1 in all places.*

Response: We have renamed "Table EV1" to "Dataset EV1" throughout the manuscript, as requested.

Editor comment 9: *Please note that EMBO press papers are accompanied online by:*
A) a short (2 sentences) summary of the findings and their significance,
B) 2-5 short bullet points highlighting the key results, and
C) a synopsis image in .jpg or .png format that is exactly 550 pixels wide and 300 pixels high. Please note that the text needs to be legible at the final size.
Please upload this information along with your revised manuscript (the text for A and B should be provided in one separate Word file uploaded as Cover Art/Synopsis Image).

Response: We have prepared and uploaded the required online materials, including **(A)** a short two-sentence summary of the findings and significance, and **(B)** 2–5 bullet points highlighting the key results, together with the synopsis image in the required format and dimensions.

Short summary: Deletion of AMPK1 in POMC neurons protects male mice against diet-induced obesity by increasing brown adipose tissue thermogenesis and energy expenditure, without affecting food intake. This effect is associated with reduced hypothalamic ER stress and ceramide content, highlighting a sex-specific and subunit-specific role of hypothalamic AMPK in energy balance.

Bullet points:

- AMPK1 ablation in POMC neurons protects male mice from diet-induced obesity.
- The metabolic phenotype is driven by increased brown adipose tissue thermogenesis and energy expenditure, not by reduced food intake.
- POMC-specific AMPK1 loss is associated with reduced hypothalamic ER stress and ceramide levels
- The effect is sexually dimorphic, as female mice do not show the same protective phenotype.

Editor comment 10: *The resolution of the submitted figures is too low for blot and microscopy images. Although there is a decent file size for all files, this reduction in resolution is commonly caused by converting original 16-bit TIFF files to RGB format for publication. While this is not inherently problematic, it can raise concerns about image integrity for critical readers. To avoid any misunderstanding and to meet EMBO Press standards, we kindly ask that you: * Resubmit the complete figure set at the captured original data resolution.**

Response: We have resubmitted the complete figure set at the original captured data resolution, ensuring that the blot and microscopy images meet EMBO Press standards.

Editor comment 11: *The Methods section "HPLC-based neurotransmitter quantification" displays a high similarity to published work. Please include a citation.*

Response: We have added an appropriate citation to the “HPLC-based neurotransmitter quantification” Methods subsection to acknowledge the published protocol on which this procedure is based.

Editor comment 12: *Our production/data editors have asked you to clarify several points in the figure legends (see below). Please incorporate these changes in the manuscript and return the revised file with tracked changes with your final manuscript submission.*

Response: We have incorporated the requested clarifications into the relevant figure legends in the manuscript and will return the revised file with tracked changes (labelled in red) together with the final submission.

- *Please note that the exact p values are not provided in the legends of figures 7D, F; 8F, G, H, I, J, K.*
  Response: We have updated the legends of Figures 7D, 7F, and 8F–K, as well as Figures 3F, H-I, to include the exact p values, as requested.

- *Please indicate the statistical test used for data analysis in the legends of figures 5C, D.*

Response: We have specified the statistical test used (Student's t-Test) for data analysis in the legends of Figures 5C and 5D, as requested.

- *Please note that the white arrow heads are not defined in the legend of figure EV5 H. This needs to be rectified.*

  Response: We have updated the legend of Figure EV5H to define the white arrowheads as indicating cells coexpressing POMC and p62.

2nd Revision - Editorial Decision 3rd Aug 2026

Prof. Miguel Lopez
University of Santiago de Compostela
Physiology
Avenida Barcelona
Spain 15782
Spain

Dear Prof. Lopez,

I am pleased to inform you that your manuscript has been accepted for publication in EMBO reports. Your manuscript will be processed for publication by EMBO Press. It will be copy edited and you will receive page proofs prior to publication. Please note that you will be contacted by Springer Nature Author Services to complete licensing and payment information.

You may qualify for financial assistance for your publication charges - either via a Springer Nature fully open access agreement or an EMBO initiative. Check your eligibility: https://link.springer.com/journal/44319/how-to-publish-with-us

Should you be planning a Press Release on your article, please notify the production team via this form in order to coordinate publication and release dates: https://forms.cloud.microsoft/e/eH4A8JVP6g

If you have any questions, please do not hesitate to contact the Editorial Office. Thank you for your contribution to EMBO Reports.

Yours sincerely,

Kurt Weir
Editor
EMBO Reports

>>> Please note that it is EMBO Reports policy for the transcript of the editorial process (containing referee reports and your response letter) to be published as an online supplement to each paper. If you do NOT want this, you will need to inform the Editorial Office via email immediately. More information is available here: https://link.springer.com/partners/embo-press/editorial-policies#Peer%20review

EMBO Press Author Checklist

Corresponding Author Name: Miguel López	
Journal Submitted to: EMBO Reports	
Manuscript Number: EMBOR-2025-62113V2	

USEFUL LINKS FOR COMPLETING THIS FORM
The EMBO Journal - Author Guidelines
EMBO Reports - Author Guidelines
Molecular Systems Biology - Author Guidelines
EMBO Molecular Medicine - Author Guidelines

Reporting Checklist for Life Science Articles (updated January
This checklist is adapted from Materials Design Analysis Reporting (MDAR) Checklist for Authors. MDAR establishes a minimum set of requirements in transparent reporting in the life sciences (see Statement of Task: 10.31222/osf.io/9sm4x). Please follow the journal's guidelines in preparing your
Please note that a copy of this checklist will be published alongside your article.

Abridged guidelines for figures
1. Data
The data shown in figures should satisfy the following conditions:
- the data were obtained and processed according to the field's best practice and are presented to reflect the results of the experiments in an accurate and unbiased manner.
- ideally, figure panels should include only measurements that are directly comparable to each other and obtained with the same assay.
- plots include clearly labeled error bars for independent experiments and sample sizes. Unless justified, error bars should not be shown for technical
- if n<5, the individual data points from each experiment should be plotted. Any statistical test employed should be justified.
- Source Data should be included to report the data underlying figures according to the guidelines set out in the authorship guidelines on Data

2. Captions
Each figure caption should contain the following information, for each panel where they are relevant:
- a specification of the experimental system investigated (eg cell line, species name).
- the assay(s) and method(s) used to carry out the reported observations and measurements.
- an explicit mention of the biological and chemical entity(ies) that are being measured.
- an explicit mention of the biological and chemical entity(ies) that are altered/varied/perturbed in a controlled manner.
- the exact sample size (n) for each experimental group/condition, given as a number, not a range;
- a description of the sample collection allowing the reader to understand whether the samples represent technical or biological replicates (including how many animals, litters, cultures, etc.).
- a statement of how many times the experiment shown was independently replicated in the laboratory.
- definitions of statistical methods and measures:
  - common tests, such as t-test (please specify whether paired vs. unpaired), simple χ2 tests, Wilcoxon and Mann-Whitney tests, can be unambiguously identified by name only, but more complex techniques should be described in the methods section;
  - are tests one-sided or two-sided?
  - are there adjustments for multiple comparisons?
  - exact statistical test results, e.g., P values = x but not P values < x;
  - definition of 'center values' as median or average;
  - definition of error bars as s.d. or s.e.m.

Please complete ALL of the questions below.
Select "Not Applicable" only when the requested information is not relevant for your study.

Materials

Newly Created Materials	Information included in the manuscript?	In which section is the information available? (Reagents and Tools Table, Materials and Methods, Figures, Data Availability Section)
New materials and reagents need to be available; do any restrictions apply?	Not Applicable	

Antibodies	Information included in the manuscript?	In which section is the information available? (Reagents and Tools Table, Materials and Methods, Figures, Data Availability Section)
For **antibodies** provide the following information:<br>- Commercial antibodies: RRID (if possible) or supplier name, catalogue number and or/clone number<br>- Non-commercial: RRID or citation	Yes	Materials and Methods

DNA and RNA sequences	Information included in the manuscript?	In which section is the information available? (Reagents and Tools Table, Materials and Methods, Figures, Data Availability Section)
**Short novel DNA or RNA including primers, probes**: provide the sequences.	Yes	Materials and Methods, Reagents and Tools Table

Cell materials	Information included in the manuscript?	In which section is the information available? (Reagents and Tools Table, Materials and Methods, Figures, Data Availability Section)
**Cell lines:** Provide species information, strain. Provide accession number in repository **OR** supplier name, catalog number, clone number, and/**OR** RRID.	Not Applicable	
**Primary cultures:** Provide species, strain, sex of origin, genetic modification status.	Not Applicable	
Report if the cell lines were recently **authenticated** (e.g., by STR profiling) and tested for mycoplasma contamination.	Not Applicable	

Experimental animals	Information included in the manuscript?	In which section is the information available? (Reagents and Tools Table, Materials and Methods, Figures, Data Availability Section)
**Laboratory animals or Model organisms:** Provide species, strain, sex, age, genetic modification status. Provide accession number in repository **OR** supplier name, catalog number, clone number, **OR** RRID.	Yes	Materials and Methods
**Animal observed in or captured from the field:** Provide species, sex, and age where possible**.**	Not Applicable	
Please detail **housing and husbandry conditions**.	Yes	Materials and Methods

Plants and microbes	Information included in the manuscript?	In which section is the information available? (Reagents and Tools Table, Materials and Methods, Figures, Data Availability Section)
**Plants:** provide species and strain, ecotype and cultivar where relevant, unique accession number if available, and source (including location for collected wild specimens).	Not Applicable	
**Microbes:** provide species and strain, unique accession number if available, and source.	Not Applicable	

Human research participants	Information included in the manuscript?	In which section is the information available? (Reagents and Tools Table, Materials and Methods, Figures, Data Availability Section)
If collected and within the bounds of privacy constraints report on age, sex and gender or ethnicity for all study participants.	Not Applicable	

Core facilities	Information included in the manuscript?	In which section is the information available? (Reagents and Tools Table, Materials and Methods, Figures, Data Availability Section)
If your work benefited from core facilities, was their service mentioned in the acknowledgments section?	Not Applicable	

Design

Study protocol	Information included in the manuscript?	In which section is the information available? (Reagents and Tools Table, Materials and Methods, Figures, Data Availability Section)
If study protocol has been **pre-registered, provide DOI in the manuscript**. For clinical trials, provide the trial registration number **OR** cite DOI.	Not Applicable	
Report the **clinical trial registration number** (at ClinicalTrials.gov or equivalent), where applicable.	Not Applicable	

Laboratory protocol	Information included in the manuscript?	In which section is the information available? (Reagents and Tools Table, Materials and Methods, Figures, Data Availability Section)
Provide DOI OR other citation details if **external detailed step-by-step protocols** are available.	Not Applicable	

Experimental study design and statistics	Information included in the manuscript?	In which section is the information available? (Reagents and Tools Table, Materials and Methods, Figures, Data Availability Section)
Include a statement about **sample size** estimate even if no statistical methods were used.	Yes	Materials and Methods, Figure Legends
Were any steps taken to minimize the effects of subjective bias when allocating animals/samples to treatment (e.g. **randomization procedure**)? If yes, have they been described?	Not Applicable	
Include a statement about **blinding** even if no blinding was done.	Yes	Materials and Methods
Describe **inclusion/exclusion criteria** if samples or animals were excluded from the analysis. Were the criteria pre-established?<br><br>If sample or data points were omitted from analysis, report if this was due to attrition or intentional exclusion and provide justification.	Yes	Materials and Methods
For every figure, are **statistical tests** justified as appropriate? Do the data meet the assumptions of the tests (e.g., normal distribution)? Describe any methods used to assess it. Is there an estimate of variation within each group of data? Is the variance similar between the groups that are being statistically compared?	Yes	Materials and Methods

Sample definition and in-laboratory replication	Information included in the manuscript?	In which section is the information available? (Reagents and Tools Table, Materials and Methods, Figures, Data Availability Section)
In the figure legends: state number of times the experiment was **replicated** in laboratory.	Yes	Figure legends
In the figure legends: define whether data describe **technical or biological replicates**.	Yes	Figure legends

Ethics

Ethics	Information included in the manuscript?	In which section is the information available? (Reagents and Tools Table, Materials and Methods, Figures, Data Availability Section)
Studies involving **human participants**: State details of **authority granting ethics approval** (IRB or equivalent committee(s), provide reference number for approval.	Not Applicable	
Studies involving **human participants**: Include a statement confirming that **informed consent** was obtained from all subjects and that the experiments conformed to the principles set out in the WMA Declaration of Helsinki and the Department of Health and Human Services Belmont Report.	Not Applicable	
Studies involving **human participants:** For publication of **patient photos**, include a statement confirming that consent to publish was obtained.	Not Applicable	
Studies involving experimental **animals**: State details of **authority granting ethics approval** (IRB or equivalent committee(s), provide reference number for approval. Include a statement of compliance with ethical regulations.	Yes	Materials and Methods
Studies involving **specimen and field samples:** State if relevant **permits** obtained, provide details of authority approving study; if none were required, explain why.	Yes	Materials and Methods

Dual Use Research of Concern (DURC)	Information included in the manuscript?	In which section is the information available? (Reagents and Tools Table, Materials and Methods, Figures, Data Availability Section)
Could your study fall under dual use research restrictions? Please check biosecurity documents and list of **select agents and toxins** (CDC): https://www.selectagents.gov/sat/list.htm	Not Applicable	
If you used a select agent, is the security level of the lab appropriate and reported in the manuscript?	Not Applicable	
If a study is subject to dual use research of concern regulations, is the name of the **authority granting approval and reference number** for the regulatory approval provided in the manuscript?	Not Applicable	

Reporting

The MDAR framework recommends adoption of discipline-specific guidelines, established and endorsed through community initiatives. Journals have their own policy about requiring specific guidelines and recommendations to complement MDAR.

Adherence to community standards	Information included in the manuscript?	In which section is the information available? (Reagents and Tools Table, Materials and Methods, Figures, Data Availability Section)
State if relevant guidelines or checklists (e.g., **ICMJE, MIBBI, ARRIVE, PRISMA**) have been followed or provided.	Yes	Materials and Methods
For **tumor marker prognostic studies,** we recommend that you follow the **REMARK** reporting guidelines (see link list at top right). See author guidelines, under 'Reporting Guidelines'. Please confirm you have followed these guidelines.	Not Applicable	
For **phase II and III randomized controlled trials**, please refer to the **CONSORT** flow diagram (see link list at top right) and submit the CONSORT checklist (see link list at top right) with your submission. See author guidelines, under 'Reporting Guidelines'. Please confirm you have submitted this list.	Not Applicable	

Data Availability

Data availability	Information included in the manuscript?	In which section is the information available? (Reagents and Tools Table, Materials and Methods, Figures, Data Availability Section)
Have **primary datasets** been deposited according to the journal's guidelines (see 'Data Deposition' section) and the respective accession numbers provided in the Data Availability Section?	Yes	Materials and Methods
Were **human clinical and genomic datasets** deposited in a public access-controlled repository in accordance to ethical obligations to the patients and to the applicable consent agreement?	Not Applicable	
Are **computational models** that are central and integral to a study available without restrictions in a machine-readable form? Were the relevant accession numbers or links provided?	Not Applicable	
If publicly available data were reused, provide the respective **data citations in the reference list**.	Not Applicable	
