# Supplementary material for: POMC AMPKα1 deletion drives male-specific BAT thermogenesis and obesity resistance
Source: EMBO Rep. 2026 Sep 1;27(20):5779–806. doi: 10.1038/s44319-026-00915-0 (PMC13694704; doi:10.1038/s44319-026-00915-0)
Supplement: Supplementary file 5 [file 44319_2026_915_MOESM4_ESM.zip › SD Figure 2-R2/2E/Male BAT Photos - 665 WT (f) - 660 KO (g).pdf]

Parameters

Parameters	
Emissivity	0.95
Refl. temp.	20 °C

31/08/2018 17:43:54

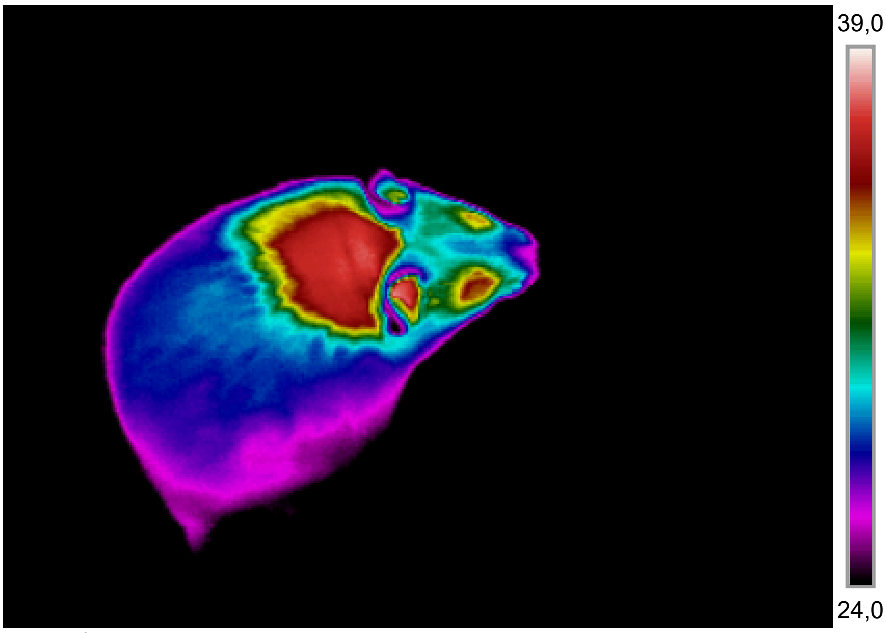

wt 665 f.jpg FLIR E60bx 64518219

31/08/2018 17:43:54

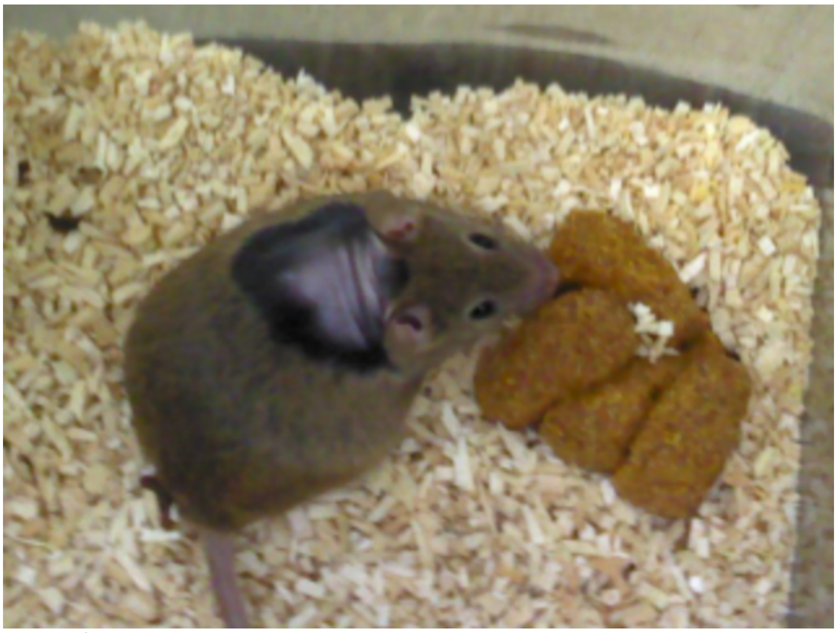

wt 665 f.jpg FLIR E60bx 64518219

Parameters

Emissivity	0.95
Refl. temp.	20 °C

31/08/2018 17:39:10

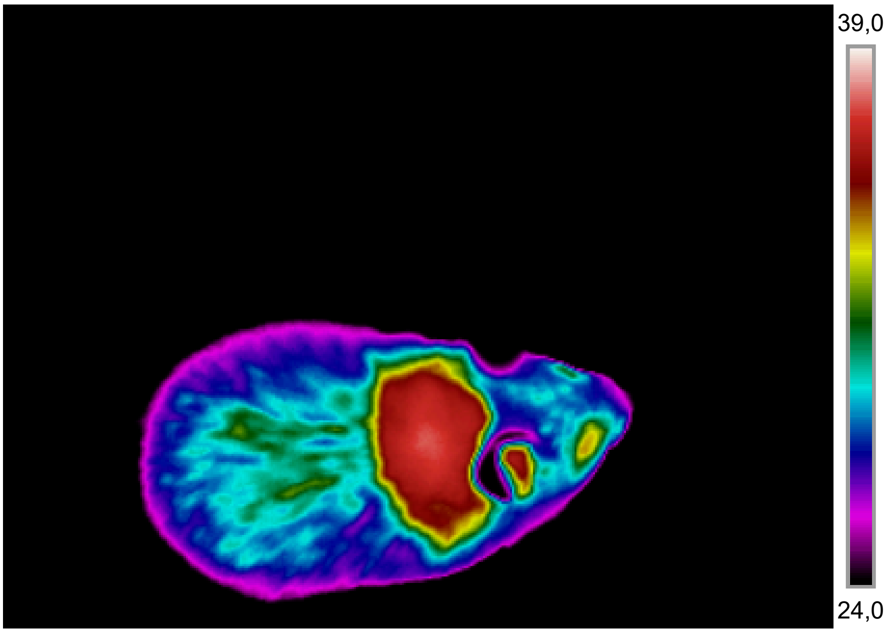

ko 660 g.jpg FLIR E60bx 64518219

31/08/2018 17:39:10

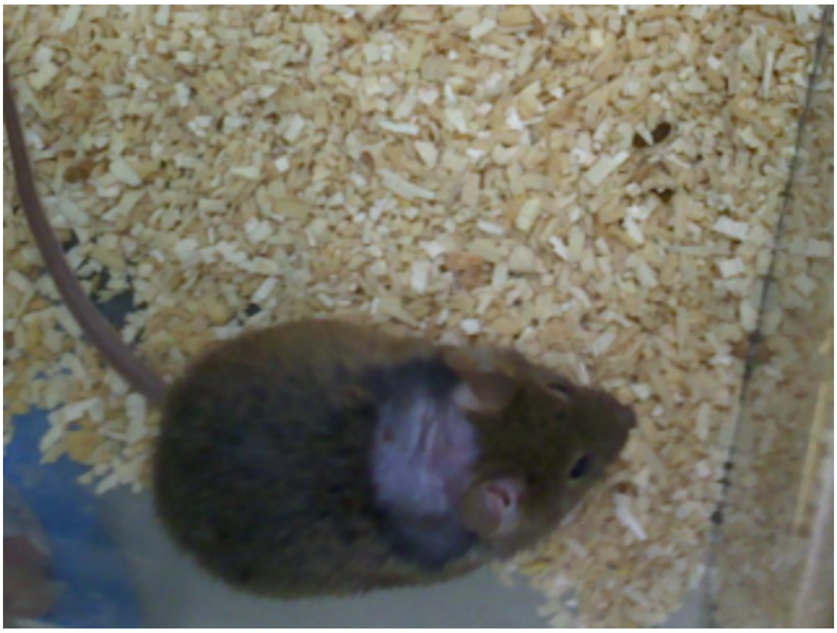

ko 660 g.jpg FLIR E60bx 64518219
