# Supplementary material for: POMC AMPKα1 deletion drives male-specific BAT thermogenesis and obesity resistance
Source: EMBO Rep. 2026 Sep 1;27(20):5779–806. doi: 10.1038/s44319-026-00915-0 (PMC13694704; doi:10.1038/s44319-026-00915-0)
Supplement: Supplementary file 6 [file 44319_2026_915_MOESM5_ESM.zip › SD Figure 3-R2/3E/HFD KO male 381a.pdf]

FLIR

03/08/2016 11:56:12 °C

39,0

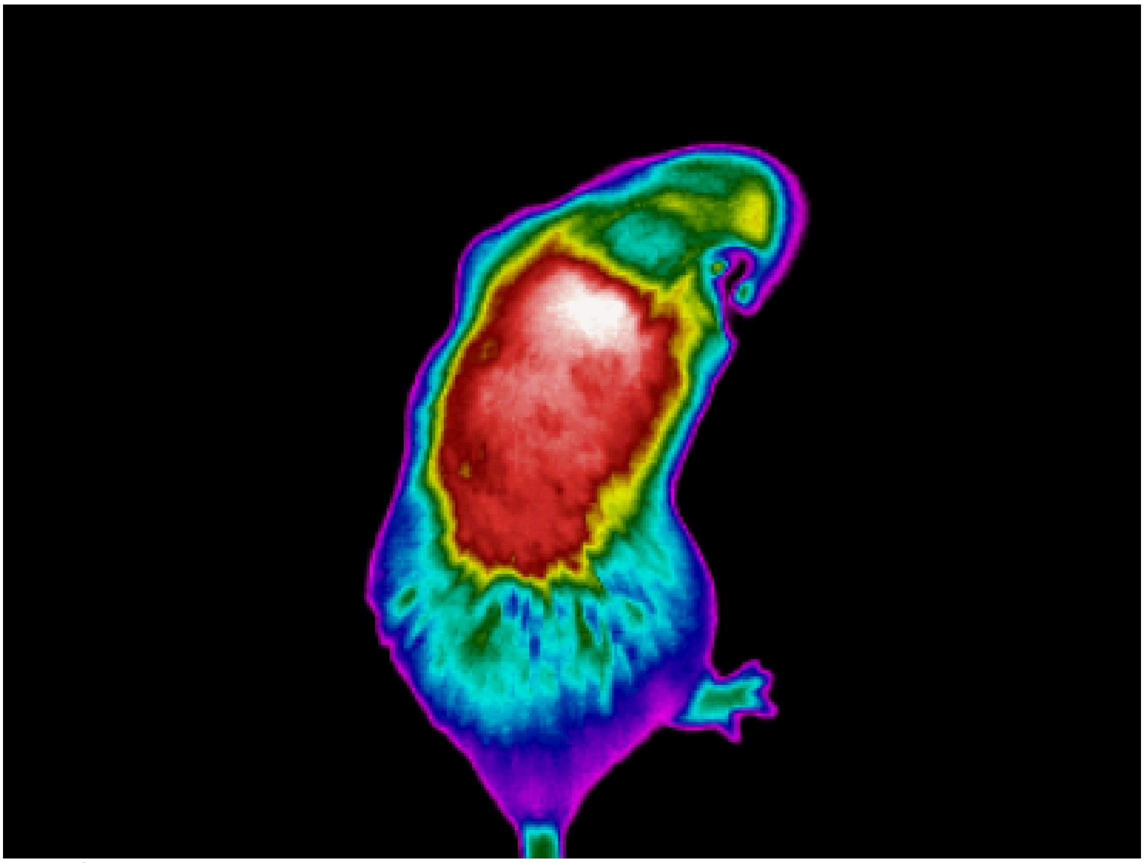

26,0

HFD KO male 381a.jpg FLIR B335 456004064

Parameters

Emissivity	0.95
Refl. temp.	20 °C
