# Supplementary material for: POMC AMPKα1 deletion drives male-specific BAT thermogenesis and obesity resistance
Source: EMBO Rep. 2026 Sep 1;27(20):5779–806. doi: 10.1038/s44319-026-00915-0 (PMC13694704; doi:10.1038/s44319-026-00915-0)

3G: UCP1 BAT

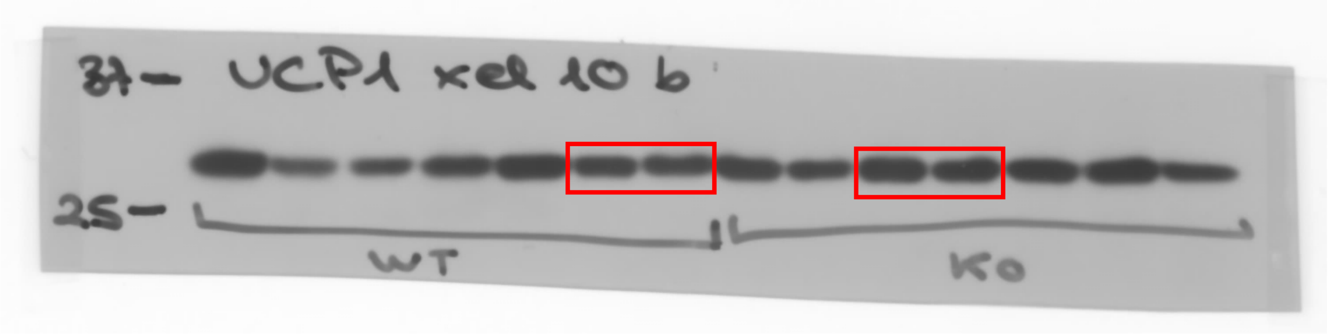

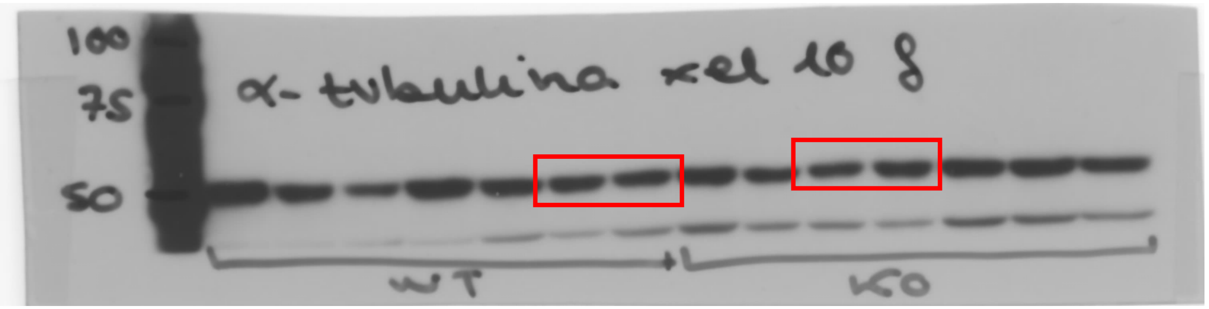

Supplement: Supplementary file 6 [file 44319_2026_915_MOESM5_ESM.zip › SD Figure 3-R2/3G/3G.pdf]
