# Supplementary material for: POMC AMPKα1 deletion drives male-specific BAT thermogenesis and obesity resistance
Source: EMBO Rep. 2026 Sep 1;27(20):5779–806. doi: 10.1038/s44319-026-00915-0 (PMC13694704; doi:10.1038/s44319-026-00915-0)
Supplement: Supplementary file 8 [file 44319_2026_915_MOESM7_ESM.zip › SD Figure 5-R2/5B/Heatmap 5B.pdf]

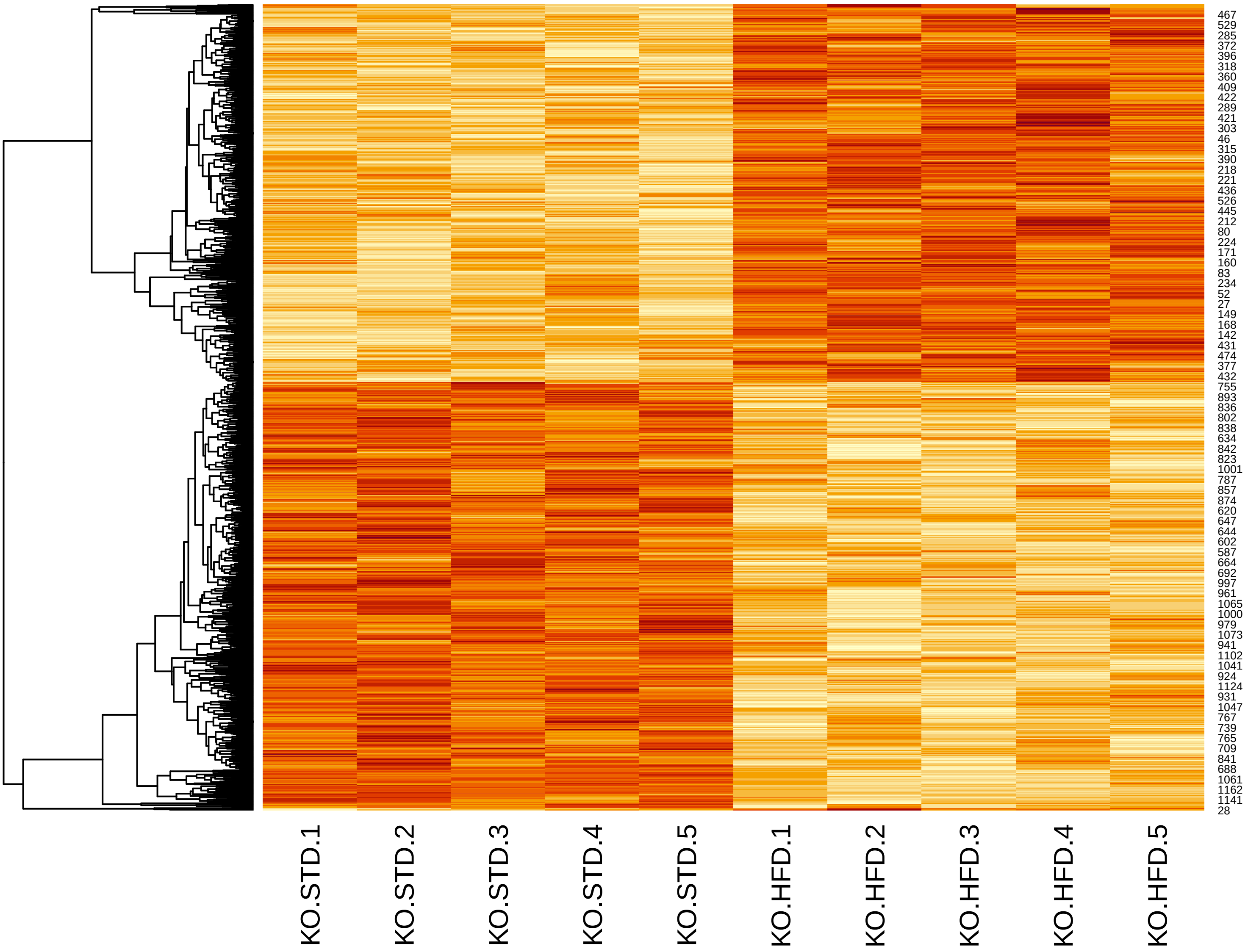
467
529
285
372
396
318
360
409
422
289
421
303
46
315
390
218
221
436
526
445
212
80
224
171
160
83
234
52
27
149
168
142
431
474
377
432
755
893
836
802
838
634
842
823
1001
787
857
874
620
647
644
602
587
664
692
997
961
1065
1000
979
1073
941
1102
1041
924
1124
931
1047
767
739
765
709
841
688
1061
1162
1141
28
KO.STD.1
KO.STD.2
KO.STD.3
KO.STD.4
KO.STD.5
KO.HFD.1
KO.HFD.2
KO.HFD.3
KO.HFD.4
KO.HFD.5
