# Supplementary material for: POMC AMPKα1 deletion drives male-specific BAT thermogenesis and obesity resistance
Source: EMBO Rep. 2026 Sep 1;27(20):5779–806. doi: 10.1038/s44319-026-00915-0 (PMC13694704; doi:10.1038/s44319-026-00915-0)

7E: ATF4 ARC

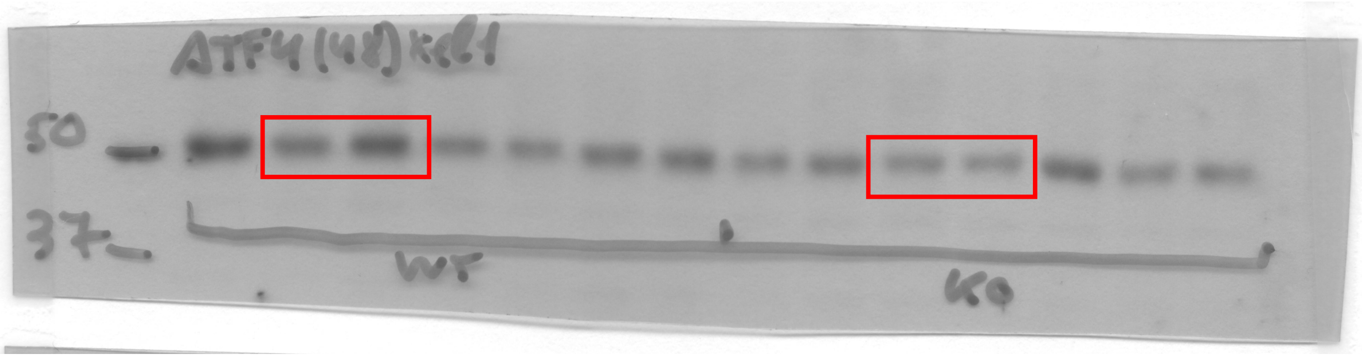

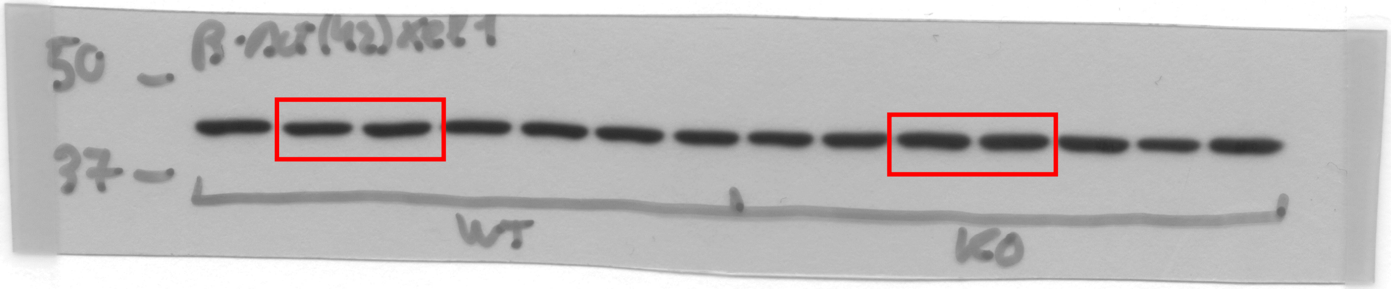

7E: CHOP_GRP78 ARC

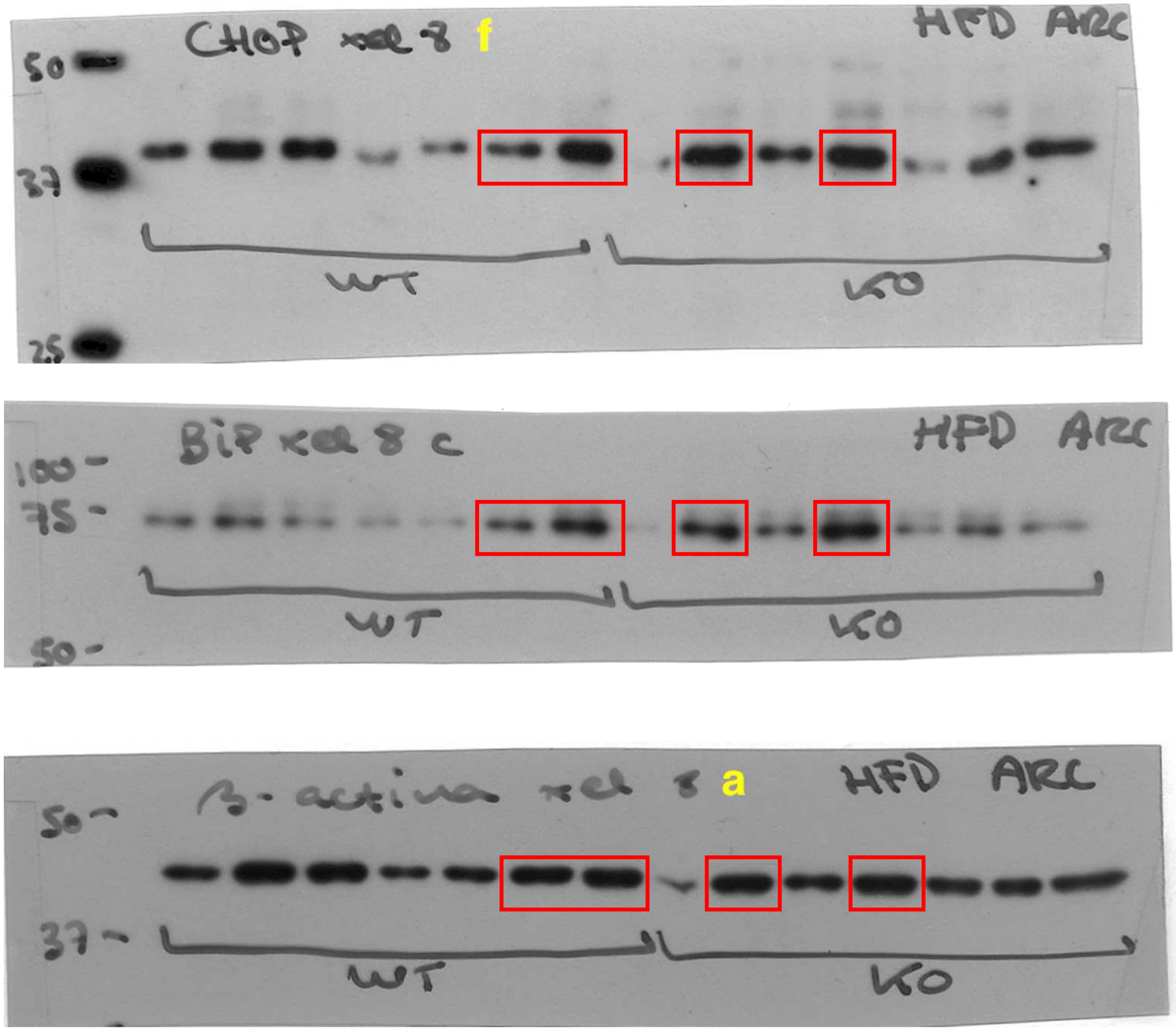

7E: IRE1α ARC

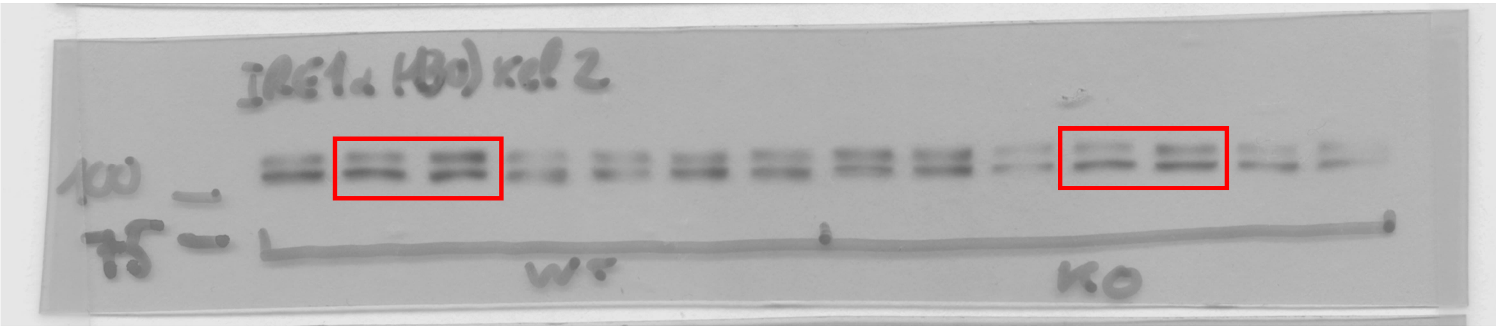

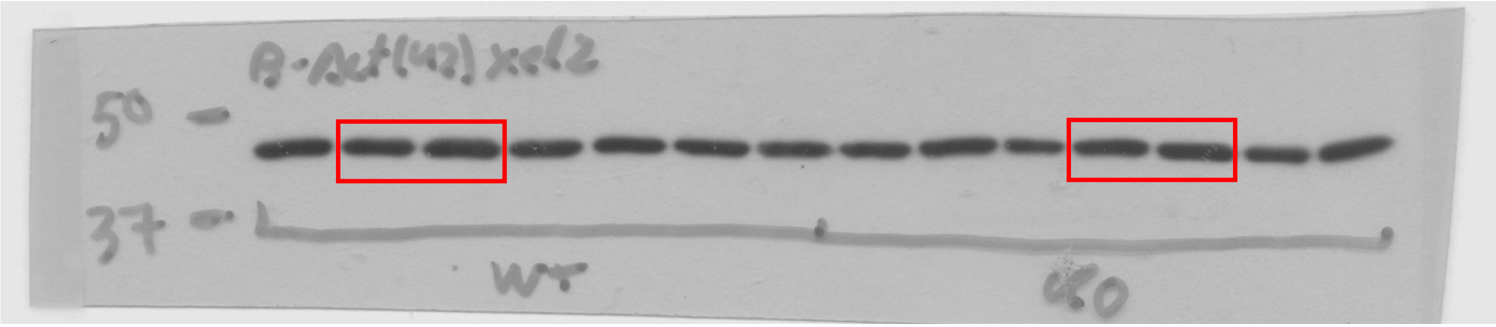

7E: pEIF2α ARC

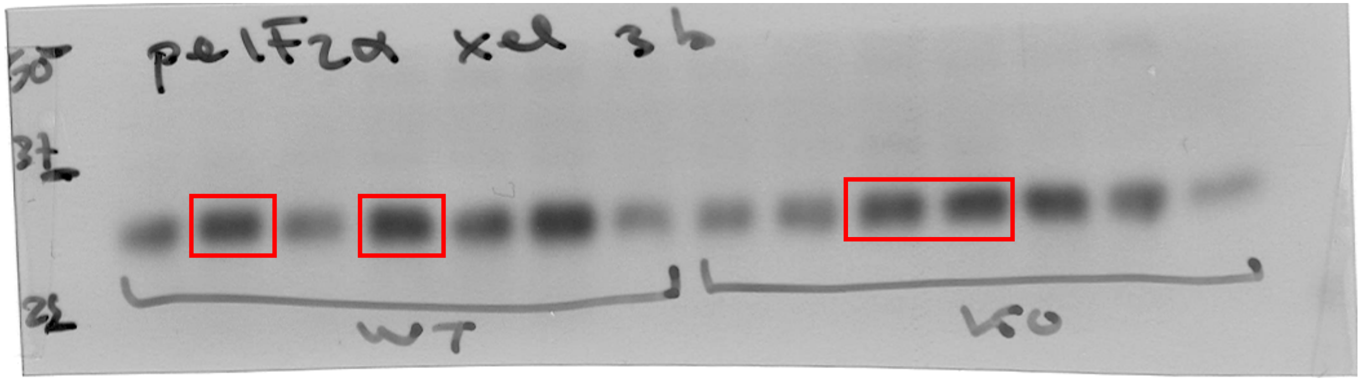

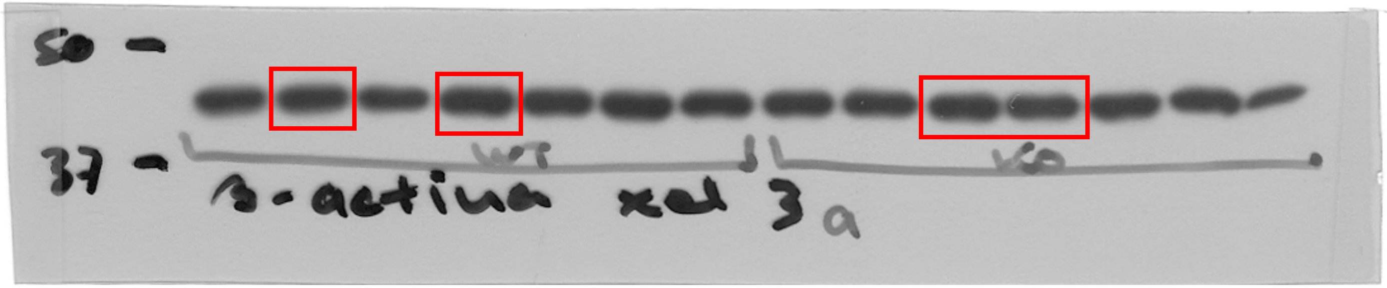

Supplement: Supplementary file 10 [file 44319_2026_915_MOESM9_ESM.zip › SD Figure 7-R2/7E/7E.pdf]
